# *qMAP* decodes RNA fragmentation dynamics in development and disease

**DOI:** 10.1038/s44320-026-00237-2

**Published:** 2026-07-20

**Authors:** Hukam C Rawal, Jiancheng Yu, Xudong Zhang, Chen Cai, Qi Chen, Tong Zhou

**Affiliations:** 1 Department of Physiology and Cell Biology, University of Nevada, Reno School of Medicine, Reno, NV USA; 2 Department of Human Genetics, University of Utah School of Medicine, Salt Lake City, UT USA; 3 Molecular Medicine Program, Division of Urology, Department of Surgery, University of Utah School of Medicine, Salt Lake City, UT USA

**Keywords:** Chromatin, Transcription & Genomics, Computational Biology, RNA Biology

## Abstract

Noncanonical small RNAs, such as tRNA-derived (tsRNAs) and rRNA-derived (rsRNAs) fragments, are more abundant than microRNAs and arise from selective cleavage events rather than random degradation. While fragmentation of parental RNAs produces functionally diverse small RNAs, current analytical approaches are limited to abundance measures and cannot systematically quantify differential cleavage signals. Here, we present *qMAP*, a computational framework profiling differential fragmentation of parental RNAs from small RNA sequencing data. *qMAP* integrates two complementary models to identify condition-specific fragmentation patterns and includes a dedicated module to pinpoint the small RNA species driving these differences. Using *qMAP*, we uncover dynamic tRNA and rRNA fragmentation during mouse cell reprogramming, demonstrate the classification power of RNA fragmentation in human ulcerative colitis, develop and validate a blood-based RNA fragmentation signature of recurrent implantation failure, and identify aging-associated RNA fragmentation in sperm, which supports RNA fragmentation as a distinct regulatory dimension beyond expression/abundance information. *qMAP* enables systematic exploration of the regulatory “RNA fragmentome”, providing a foundational tool for both mechanistic discovery and translational applications of noncanonical small RNAs.

## Synopsis

*qMAP* is a computational framework quantifying differential fragmentation of parental RNAs, which reveals “RNA Fragmentome” as a distinct regulatory layer. Across cell development, diseases, and sperm aging, *qMAP* uncovers RNA fragmentation dynamics with mechanistic and translational relevance.

- *qMAP* advances small RNA analysis beyond abundance by quantifying fragmentation patterns along parental RNAs and pinpointing the small RNA species that drive these shifts.
- During mouse somatic cell reprogramming, *qMAP* reveals widespread remodeling of tRNA and rRNA fragmentation that is largely uncoupled from overall small RNA abundance.
- RNA fragmentation patterns outperform traditional abundance-based profiles in classification of ulcerative colitis disease states and define a non-invasive, blood-based signature of recurrent implantation failure.
- *qMAP* pinpoints sperm aging-associated rsRNAs underlying differential fragmentation patterns, enabling functional exploration of candidate regulatory fragments.

## Abstract (web summary)

*qMAP* is a computational framework quantifying differential fragmentation of parental RNAs, which reveals “RNA Fragmentome” as a distinct regulatory layer. Across cell development, diseases, and sperm aging, *qMAP* uncovers RNA fragmentation dynamics with mechanistic and translational relevance.

## Graphical abstract

**Figure:**
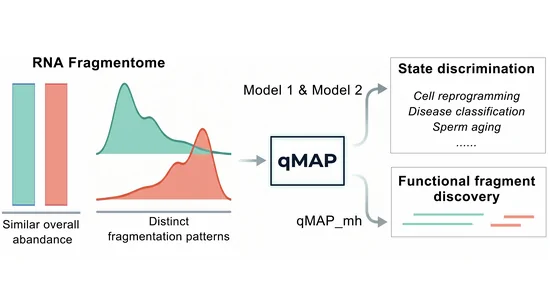

**Figure:**
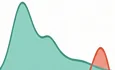

## Introduction

In recent years, our understanding of small noncoding RNAs (sncRNAs) has fundamentally shifted (Shi et al, 2022). Improved sncRNA sequencing methods such as PANDORA-seq (Shi et al, 2021), which overcome RNA modification barriers, revealed that classical microRNAs (miRNAs) constitute a very limited portion of the total sncRNA pool. This exposed a vast, previously hidden small RNA world dominated by tRNA- and rRNA-derived small RNAs (tsRNAs and rsRNAs), along with diverse sncRNAs derived from various RNAs such as Y RNAs (ysRNAs), snoRNAs (snosRNAs), snRNAs (snsRNAs), and Vault RNAs (vtsRNAs) (Shi et al, 2022). Far from being products of random degradation, these noncanonical sncRNAs originate from highly selective and regulated fragmentation of their parental RNAs (Chen et al, 2021; Jouravleva and Zamore, 2025), generating reproducible profiles across tissue/cell types, which respond dynamically to various stresses and are involved in diverse biological processes and disease conditions including cancer progression (Balatti et al, 2017; Goodarzi et al, 2015; Liu et al, 2022; Su et al, 2022), hypoxia (Li et al, 2025a), immune modulation (Cambier et al, 2017; Pawar et al, 2024; Pawar et al, 2020; Yue et al, 2021), neurological diseases (Zhang et al, 2025; Zhang et al, 2020), cardiovascular diseases (Hernandez et al, 2023; Li et al, 2025b), sperm quality and epigenetic inheritance (Alata Jimenez et al, 2023; Cai and Chen, 2024; Chen et al, 2016a; Chen et al, 2016b; Isacson et al, 2025; Natt et al, 2019; Sharma et al, 2016; Shi et al, 2026; Tomar et al, 2024; Zhang et al, 2018), and symbiosis (Ren et al, 2019).

Beyond their emerging biological roles in stress and disease responses, growing evidence shows that this regulated fragmentation process generates functionally distinct molecules, representing a process of evolutionary refinement vividly described as RNA “*renovatio*” (from Latin, meaning renewal, renovation, and rebirth) (Kuhle et al, 2023). Fragments cleaved from the same parental RNA can differ in structural motifs, protein interactors, and subcellular localization, endowing them with unique functions (Kuhle et al, 2023). For example, 5’-halves of tRNAs can have distinct roles compared with 3’-halves or internal fragments due to obvious sequence differences; the functional roles of tsRNAs are strongly influenced by the specific cleavage sites on tRNAs or pre-tRNAs from which they are derived (Cho et al, 2019; Su et al, 2019); and even slight length variations from 3’-end of a same tRNA can lead to distinct modes of action (Schorn et al, 2017). These fragmentation patterns are highly regulated, stress-responsive, and tissue-specific, and their dysregulation is increasingly linked to metabolic disorders, cancer, and autoimmune diseases (Chen and Zhou, 2023; Yu et al, 2025). This complex fragmentation creates a new, unappreciated layer of biological regulation analogous to alternative splicing in mRNAs, i.e., different mRNA isoforms from one gene yield distinct proteins, while different sncRNA fragments from one parental RNA represent functionally diverse molecules. We term this vast regulatory layer the “RNA fragmentome”, defined as the collection of condition-specific fragmentation patterns of parental RNAs.

However, a fundamental obstacle has prevented systematic investigation of the concept of RNA fragmentome: there is currently no robust method to quantify differential fragmentation of parental RNAs from sncRNA sequencing (sncRNA-seq) data. Detecting the presence of fragments does not reveal the fragmentation pattern—analogous to identifying exons without measuring splicing. Thus, the field lacks the tools required to define the RNA fragmentome as a regulatory dimension. To address this gap, we developed *qMAP* (quantitative mapping for differential fragmentation, available at https://github.com/cozyrna/qMAP), a computational framework to systematically detect and quantify condition-specific changes in parental RNA fragmentation from sncRNA-seq data. *qMAP* integrates two complementary models to identify differentially fragmented parental RNAs and includes a dedicated module, *qMAP_mh*, to pinpoint the specific sncRNA species driving the pattern shift. By moving beyond simple abundance to decode fragmentation signatures, we show that *qMAP* reveals dynamic fragmentation patterns of sncRNAs (using tsRNAs and rsRNAs as examples) during cell reprogramming, improves disease classification compared to abundance-based methods, and identifies a non-invasive fragmentation signature for reproductive disorders. These findings demonstrate that RNA fragmentation is a biologically meaningful and clinically actionable regulatory layer, thereby establishing *qMAP* as a foundational tool for systematic exploration of the RNA fragmentome.

## Results

### *qMAP*: a computational framework to explore RNA fragmentome

**Figure 1:**
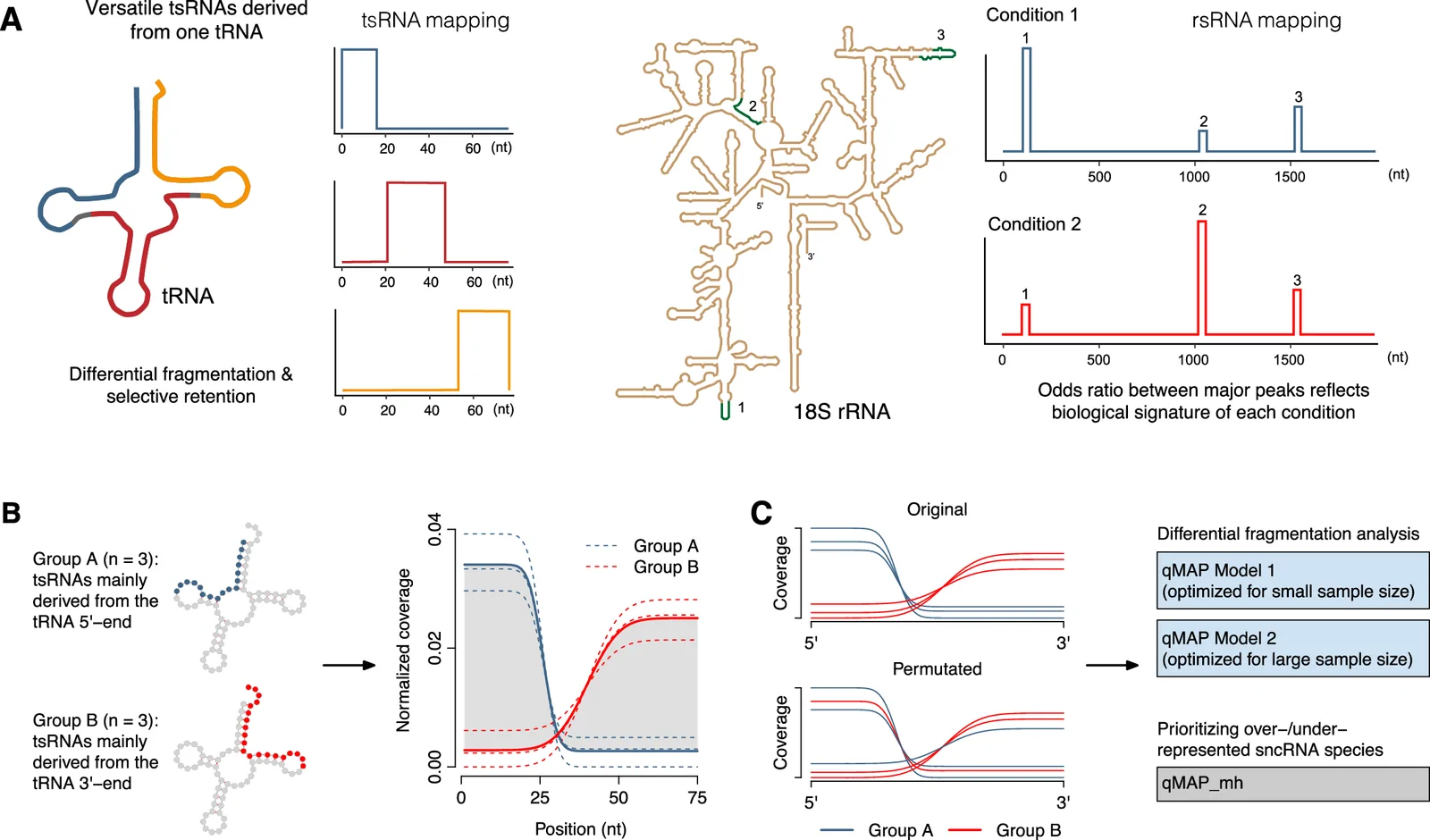
Overview of *qMAP.* (**A**) Schematic diagram showing RNA differential fragmentation of tRNA (left panel) and rRNA (right panel). A single parental RNA can undergo differential fragmentation to generate distinct sncRNA species, and the differential cleavage ratios (e.g., of rsRNAs derived from 18S rRNA) reflect distinct biological conditions. The secondary structure template of 18S rRNA was obtained from RNAcentral (https://rnacentral.org). (**B**) Normalized coverage and coverage difference. We assume the fragmentation pattern of a given tRNA (75 nt long) is compared between Group A and Group B. The tsRNAs of Group A and Group B are mainly derived from the 5’-end and 3’-end of the parental tRNA, respectively. The normalized coverage of the tsRNAs is subsequently plotted along the parental tRNA for the individual samples within the two groups, illustrated as the blue/red dash curves. The solid blue and red curves indicate the mean coverage pattern for Group A and Group B, respectively. The gray area surrounded by the two solid curves represents the coverage difference between the two groups. (**C**) Computational strategy of *qMAP*. *qMAP* Model 1 and Model 2 rely on permutating samples between the groups to infer the statistical significance of the observed differential fragmentation, and *qMAP_mh* applies the Mantel–Haenszel procedure to identify the sncRNA species contributing to the observed differential fragmentation.

Over the years, we have developed a series of tools to explore the expanding universe of sncRNAs, including PANDORA-seq (Shi et al, 2021) to reveal hidden sncRNAs (e.g., tsRNAs and rsRNAs) that were previously masked by RNA modifications, *SPORTS* (Shi et al, 2018) for accurate annotation and profiling of noncanonical sncRNAs, and *FUSION* (Rawal et al, 2025) to improve the robustness of differential abundance analysis by integrating signals across related fragment families. Yet despite these advances, one major regulatory dimension has remained unquantified: the fragmentation pattern itself (Fig. 1A). Even though these cleavage patterns reflect distinct biogenesis pathways and functional potential, their summed abundance may appear similar, thus abundance-based traditional pipelines would overlook biologically critical fragmentation shifts. Moreover, abundance measurements are sensitive to sequencing depth and normalization strategies, complicating cross-dataset comparisons (Shi et al, 2022). By contrast, we reasoned that the internal distribution of fragments along a parental RNA—the relative proportion of fragments at each base position—should be more stable and condition-specific. Such “normalized coverage” could reflect intrinsic fragmentation signatures independent of total abundance, enabling quantitative comparisons across samples and platforms (Fig. 1A).

Guided by these principles, we developed *qMAP* to quantify RNA fragmentome. Normalized coverage along a parental RNA is calculated by quantifying, at each base position, the normalized amount of sequencing reads (i.e., sncRNAs) mapped to that parental RNA. This approach allows for comparison of the relative coverage at each position, independent of the overall abundance of the sncRNA family, and ensures that the area under the coverage curve equals one (Fig. 1B). The *qMAP* framework consists of three computational elements: *qMAP* Model 1, *qMAP* Model 2, and *qMAP_mh*. *qMAP* Models 1 and 2 are designed to identify differentially fragmented parental RNAs by assessing the difference in normalized coverage between biological conditions. Permutation tests are applied in both models: Model 1 relies on a linear model to determine whether differences in normalized coverage exist across positions between groups, which is suitable for a study with limited samples (e.g., 3 *vs*. 3), while Model 2 is a faster, non-parametric strategy for larger datasets (see “Methods” for details) (Fig. 1C). To further identify the specific sncRNA species driving the observed fragmentation changes, we developed the *qMAP_mh* module. This module applies the Mantel–Haenszel procedure (Mantel and Haenszel, 1959) to prioritize sncRNA species that are significantly over- or under-represented between conditions, pinpointing the fragments contributing to the differential fragmentation (see “Methods” for details) (Fig. 1C). Together, these modules enable high-resolution dissection of parental RNA fragmentation dynamics as well as the underlying regulatory small RNAs.

### *qMAP* reveals a dynamic RNA fragmentome during cell reprogramming

**Figure 2:**
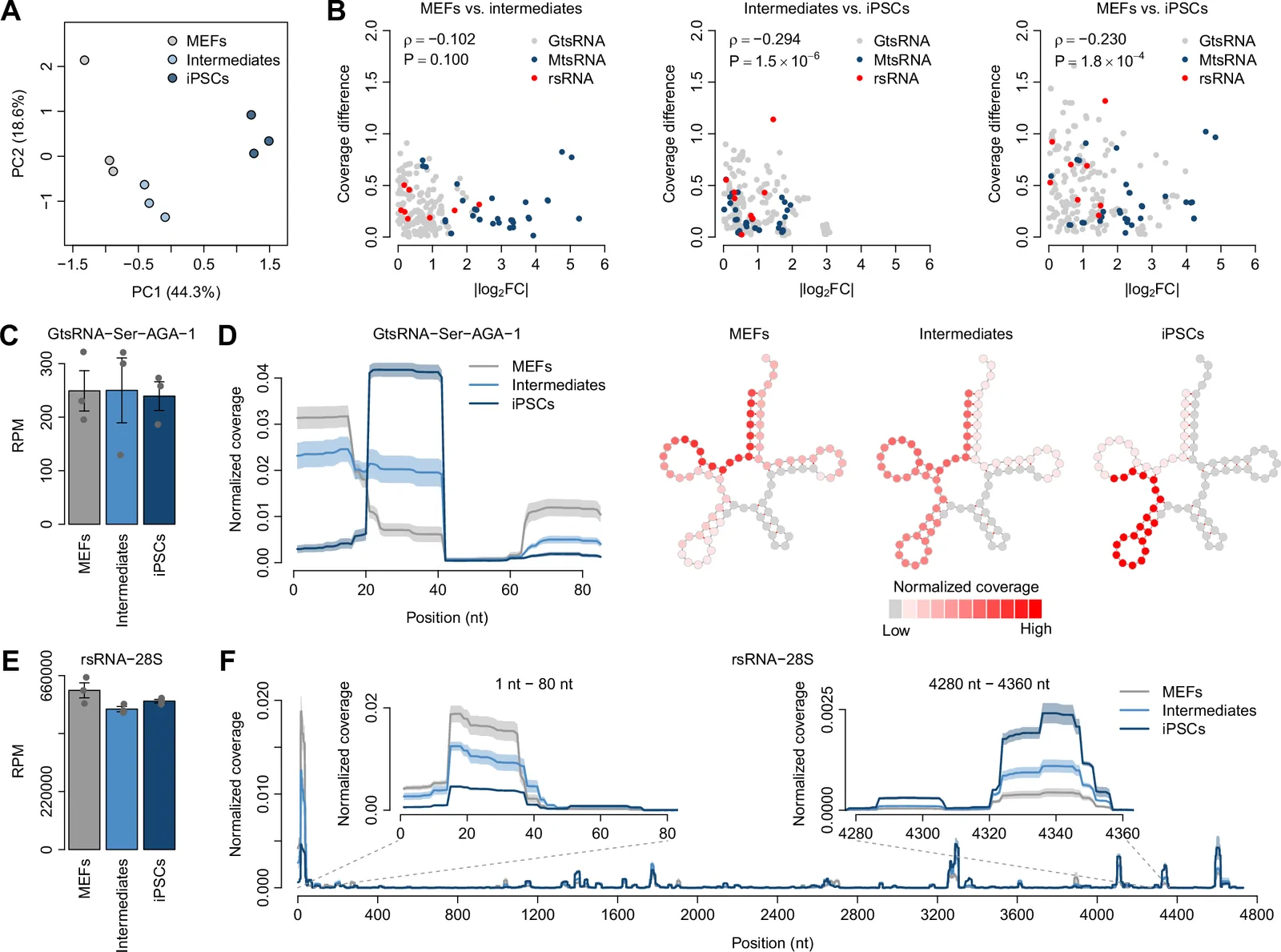
Dynamic sncRNA fragmentation during iPSC induction. (**A**) PCA on the tsRNA/rsRNA coverage pattern of MEFs, intermediates, and iPSCs. PC1: the first principal component; PC2: the second principal component. (**B**) Relationship between differential abundance and differential fragmentation. Each dot represents one tsRNA/rsRNA subfamily. *X* axis denotes the absolute value of log_2_-transformed fold change ( | log_2_*FC* | ) in abundance of the individual sncRNA subfamilies; *Y* axis denotes the corresponding coverage difference. The correlation coefficient (*ρ*) and *P* value were calculated using *Spearman*’s rank correlation test. (**C**) Comparison in abundance (*RPM*) of GtsRNA-Ser-AGA-1 between MEFs, intermediates, and iPSCs (*n* = 3 per group). The bars and the error bars represent the mean ± SEM. No significant difference was observed between the individual cell stages (*t-*test: *P* > 0.05). (**D**) Comparison in coverage pattern of GtsRNA-Ser-AGA-1 between MEFs, intermediates, and iPSCs. The solid curves indicate the mean normalized coverage along the parental tRNA, and the bands represent the SEM. The colored nucleotides within the tRNA secondary structures highlight the tsRNA coverage level. Significant differences in normalized coverage were observed between the individual cell stages (*qMAP* Model 1: coverage difference [*CD*] = 0.549 and adjusted *P* < 10^−10^ between MEFs and intermediates; *CD* = 0.899 and adjusted *P* < 10^−10^ between intermediates and iPSCs; *CD* = 1.439 and adjusted *P* < 10^−10^ between MEFs and iPSCs). (**E**) Comparison in abundance (*RPM*) of rsRNA-28S between MEFs, intermediates, and iPSCs (*n* = 3 per group). The bars and the error bars represent the mean ± SEM. No significant difference was observed between the individual cell stages (*t-*test: *P* > 0.05). (**F**) Comparison in coverage pattern of rsRNA-28S between MEFs, intermediates, and iPSCs. The solid curves indicate the mean normalized coverage along the parental rRNA, and the bands represent the SEM. The inner panels provide a zoom-in view of two exemplary rRNA regions. Significant differences in normalized coverage were observed between the individual cell stages (*qMAP* Model 1: *CD* = 0.503 and adjusted *P* < 10^−10^ between MEFs and intermediates; *CD* = 0.554 and adjusted *P* < 10^−10^ between intermediates and iPSCs; *CD* = 0.922 and adjusted *P* < 10^−10^ between MEFs and iPSCs). Source data are available online for this figure.

To test whether RNA fragmentation represents an independent regulatory layer, we first applied *qMAP* to a well-controlled PANDORA-seq dataset, which we generated to capture the dynamic reprogramming process from mouse embryonic fibroblasts (MEFs) to reprogramming intermediate cells (day 3, intermediates), and finally to induced pluripotent stem cells (iPSCs) (Shi et al, 2021). We focused on the two dominant noncanonical sncRNA classes: tsRNAs (including genomic tsRNAs [GtsRNAs] and mitochondrial tsRNAs [MtsRNAs]) and rsRNAs. A principal component analysis (PCA) based on the fragmentation patterns revealed a clear, stage-specific separation of all three cell types (Fig. 2A), indicating that the RNA fragmentome is indeed distinct across the individual developmental stages. Using *qMAP* Model 1 (optimal for *n* = 3 per group), we identified 111, 127, and 162 parental RNAs showing differential fragmentation (see “Methods” for details) between MEFs and intermediates, between intermediates and iPSCs, and between MEFs and iPSCs, respectively (Table EV1), which suggests the broad rewiring of RNA cleavage during cell fate transition.

**Figure EV1:**
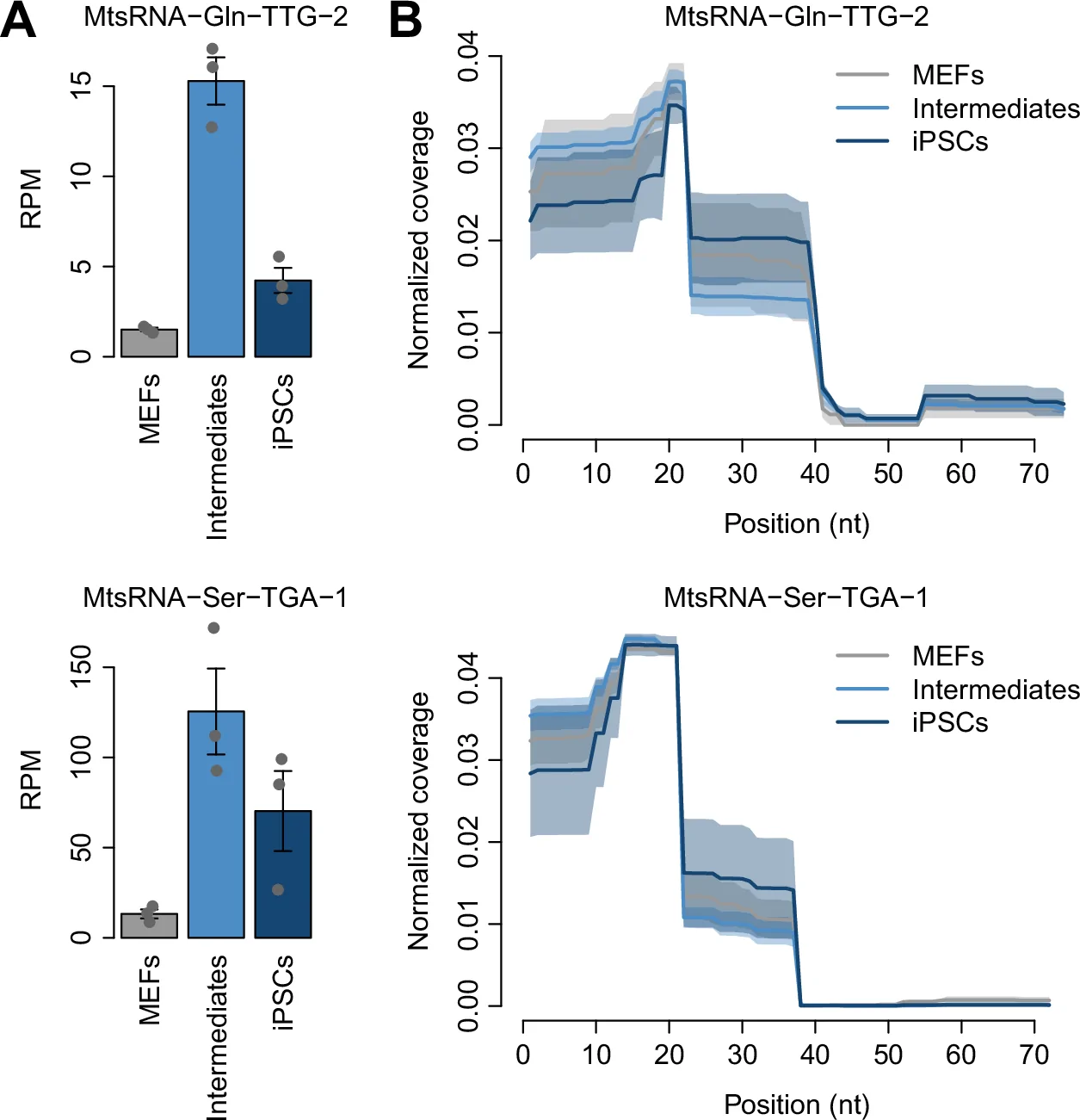
Dynamic sncRNA abundance during iPSC induction. (**A**) Comparison in abundance (*RPM*) of MtsRNA-Gln-TTG-2 and MtsRNA-Ser-TGA-1 between MEFs, intermediates, and iPSCs (*n* = 3 per group). The bars and the error bars represent the mean ± SEM. *t-*test was used to compare the difference in *RPM*: for MtsRNA-Gln-TTG-2, *P* = 8.7 × 10^−3^ between MEFs and intermediates, *P* = 4.8×10^−3^ between intermediates and iPSCs, and *P* = 0.056 between MEFs and iPSCs; for MtsRNA-Ser-TGA-1, *P* = 0.041 between MEFs and intermediates, *P* = 0.165 between intermediates and iPSCs, and *P* = 0.122 between MEFs and iPSCs. (**B**) Comparison in coverage pattern of MtsRNA-Gln-TTG-2 and MtsRNA-Ser-TGA-1 between MEFs, intermediates, and iPSCs. The solid curves indicate the mean normalized coverage along the parental tRNAs, and the bands represent the SEM. No significant difference in normalized coverage was observed between the individual cell stages (*qMAP* Model 1: *P* > 0.05).

**Figure EV2:**
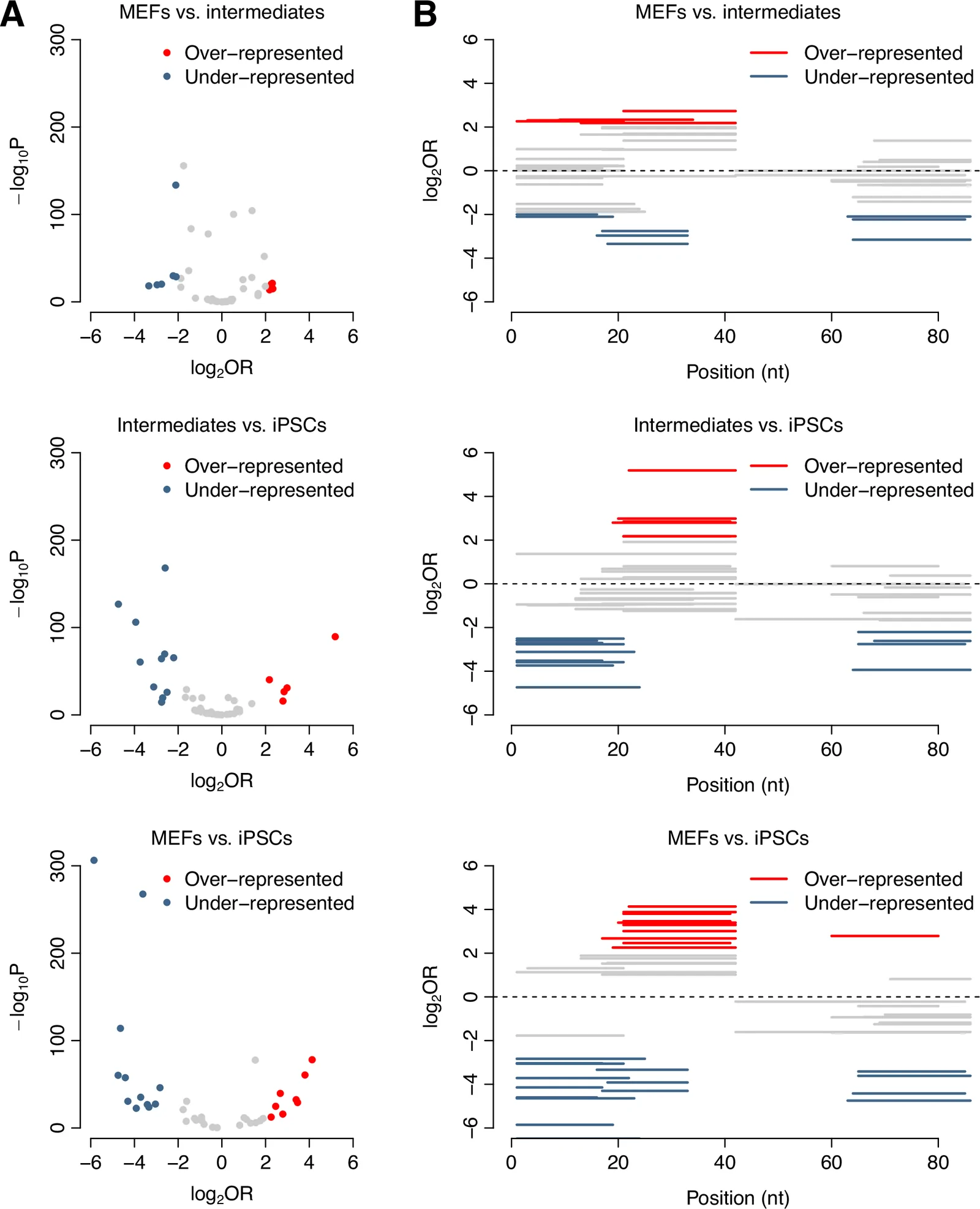
GtsRNA-Ser-AGA-1 species that are involved in differential fragmentation during iPSC induction. (**A**) Volcano plots showing the over-/under-represented GtsRNA-Ser-AGA-1 species (*n* = 3 per group). *X* axis: log_2_-transformed odds ratio (log_2_*OR*); *Y* axis: log_10_-transformed *P* value. The odds ratios and *P* values were calculated using the *qMAP_mh* module. The data points with *P* value = 0 were excluded from the volcano plots. The tsRNA species with adjusted *P* < 0.05 and log_2_*OR* > 2 were considered as over-represented, while the tsRNA species with adjusted *P* < 0.05 and log_2_*OR* < -2 were considered as under-represented. The baselines of the individual comparisons are MEFs, intermediates, and MEFs for the upper, middle, and lower panels, respectively. (**B**) Mapping patterns of the over-/under-represented tsRNA species along the parental tRNA.

**Figure EV3:**
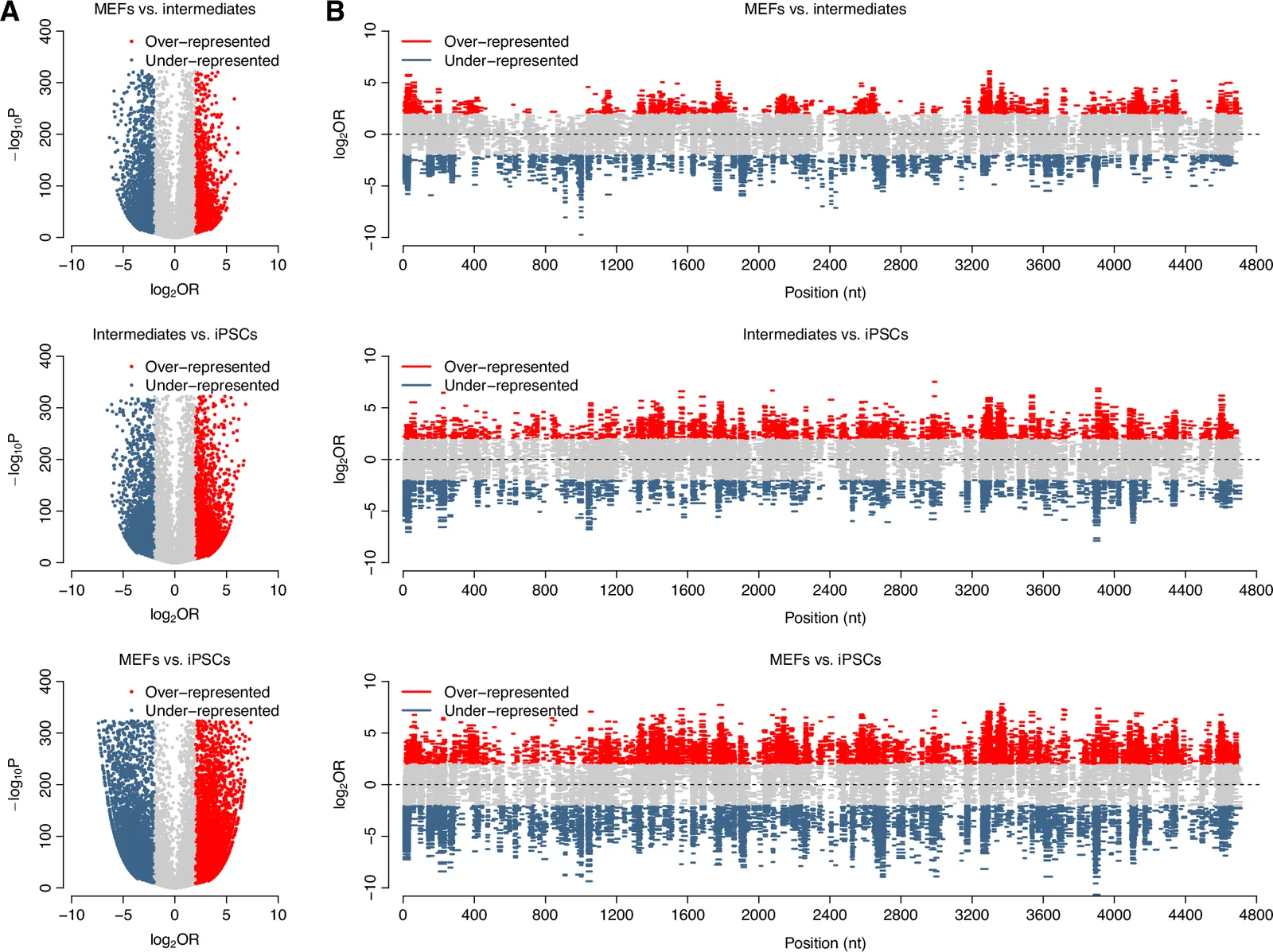
rsRNA-28S species that are involved in differential fragmentation during iPSC induction. (**A**) Volcano plots showing the over-/under-represented rsRNA-28S species (*n* = 3 per group). *X* axis: log_2_-transformed odds ratio (log_2_*OR*); *Y* axis: log_10_-transformed *P* value. The odds ratios and *P* values were calculated using the *qMAP_mh* module. The data points with *P* value = 0 were excluded from the volcano plots. The rsRNA species with adjusted *P* < 0.05 and log_2_*OR* > 2 were considered as over-represented, while the rsRNA species with adjusted *P* < 0.05 and log_2_*OR* < -2 were considered as under-represented. The baselines of the individual comparisons are MEFs, intermediates, and MEFs for the upper, middle, and lower panels, respectively. (**B**) Mapping patterns of the over-/under-represented rsRNA species along the parental rRNA.

We next examined whether RNA fragmentation dynamics simply mirrored abundance changes. Intriguingly, we did not observe a positive correlation between abundance change and coverage difference (i.e., difference in fragmentation) during cell reprogramming. In fact, no correlation or even weak negative correlation was observed (Fig. 2B). This finding provides strong evidence that dynamic RNA fragmentation is a process largely independent of abundance-based regulation and encodes information not captured by abundance levels alone. This distinction is striking at the level of individual sncRNA subfamilies. For instance, GtsRNA-Ser-AGA-1 subfamily showed comparable abundance levels (in reads per million [*RPM*]) across all three cell stages (Fig. 2C), a finding that may be dismissed in a standard abundance-based analysis. However, *qMAP* revealed a profound and progressive shift in its coverage pattern along the parental tRNA (Fig. 2D). Similar dynamic fragmentation was also observed for longer parental RNAs like 28S rRNA. We didn’t observe a significant difference in abundance of rsRNA-28S between the cell stages (Fig. 2E), while the coverage pattern of rsRNA-28S differed dramatically across distant regions (Fig. 2F). These examples indicate that cleavage sites are reprogrammed even when overall sncRNA abundance from the parental RNA remains constant. In contrast, some sncRNA subfamilies, such as MtsRNA-Gln-TTG-2 and MtsRNA-Ser-TGA-1, showed variable abundance profiles during cell reprogramming (Fig. EV1A) while maintaining a highly stable and similar coverage pattern across all three stages (Fig. EV1B). For the parental RNAs undergone differential fragmentation, e.g., genomic tRNA-Ser-AGA-1 (GtRNA-Ser-AGA-1) and 28S rRNA, we further employed the *qMAP_mh* module to screen the sncRNA species driving the observed shift in parental RNA fragmentation, which yielded a spectrum of over-/under-represented tsRNA/rsRNA species (Figs. EV2A,B and EV3A,B). These prioritized tsRNA/rsRNA species represent candidate functional fragments for future mechanistic studies. Taken together, these results demonstrate that *qMAP* uncovers a biologically meaningful dimension of sncRNA regulation that is hidden from traditional abundance (or expression) based analyses. The RNA fragmentome is actively remodeled during cell fate transitions, suggesting a specialized role for cleavage selectivity in developmental reprogramming.

### RNA fragmentation in ulcerative colitis

**Figure 3:**
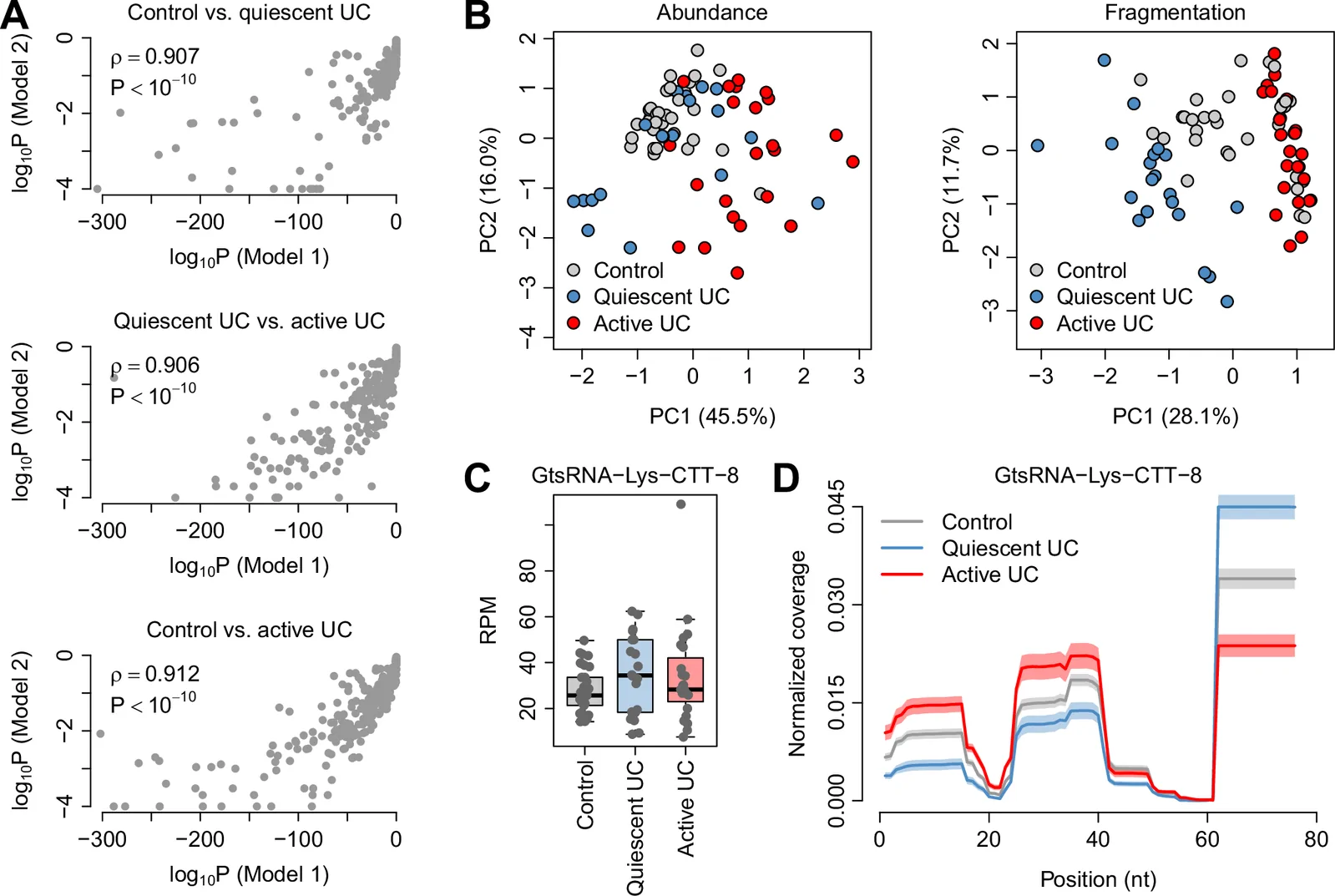
Distinct sncRNA fragmentation in UC. (**A**) Correlation in *qMAP P* value between Model 1 and Model 2. Each dot represents one tRNA/rRNA subfamily. The data points with *qMAP P* value = 0 for either Model 1 or Model 2 were excluded from the scatter plots. The correlation coefficient (*ρ*) and *P* value were calculated using *Spearman*’s rank correlation test (*ρ* = 0.907 and *P* = 1.2 × 10^−107^ for control *vs*. quiescent UC, *ρ* = 0.906 and *P* = 3.5 × 10^−107^ for quiescent UC *vs*. active UC, and *ρ* = 0.912 and *P* = 6.5 × 10^−111^ for control *vs*. active UC). (**B**) PCA on tsRNA/rsRNA abundance and fragmentation. PC1 the first principal component, PC2 the second principal component. (**C**) Comparison in abundance (*RPM*) of GtsRNA-Lys-CTT-8 between controls (*n* = 33), patients with quiescent UC (*n* = 20), and patients with active UC (*n* = 23). In each box, the upper and lower bounds represent the 75th and 25th percentiles (*Q*_*3*_ and *Q*_*1*_), respectively. The upper and lower whiskers stand for the most extreme data points within *Q*_*3*_ + 1.5×(*Q*_*3*_–*Q*_1_) and *Q*_1_–1.5×(*Q*_3_–*Q*_1_), respectively. The median of the data points is indicated by the horizontal line inside the box. No significant difference was observed between the individual groups (*t-*test: *P* > 0.05). (**D**) Comparison in coverage pattern of GtsRNA-Lys-CTT-8 between controls, patients with quiescent UC, and patients with active UC. The solid curves indicate the mean normalized coverage along the parental tRNA, and the bands represent the SEM. Significant differences in normalized coverage were observed between the individual groups (*qMAP* Model 1: coverage difference [*CD*] = 0.329 and *P* < 10^−10^ between controls and patients with quiescent UC; *CD* = 0.637 and *P* < 10^−10^ between patients with quiescent and active UC; *CD* = 0.320 and *P* < 10^−10^ between controls and patients with active UC). Source data are available online for this figure.

Having established *qMAP* in a controlled mouse system, we next tested whether fragmentation patterns carry clinically relevant information in complex human tissues. Here, we investigated a sncRNA-seq dataset from the Gene Expression Omnibus (GEO) database (Edgar et al, 2002) (GEO accession: GSE185102), which comprises colon tissue data from 33 controls, 20 patients with quiescent ulcerative colitis (UC), and 23 patients with active UC (Inciuraite et al, 2024). Both *qMAP* Model 1 and Model 2 were applied to this dataset (Table EV2), revealing a strong positive correlation in statistical output (Fig. 3A), which suggests a high robustness between the two computational strategies. PCA on the fragmentation pattern, i.e., the coverage along parental RNAs, indicates a clear separation between the quiescent and active UC patients with the controls in between, whereas PCA on the abundance of tsRNA/rsRNA subfamilies failed to do so (Fig. 3B). This contrast indicates that RNA fragmentation encodes biological variation not captured by RNA abundance/expression levels. For example, GtsRNA-Lys-CTT-8 subfamily shows comparable abundance between the controls and patients (Fig. 3C) but exhibits a clear shift in coverage patterns (Fig. 3D), which supports our above-mentioned perspective that RNA fragmentation comprises an additional layer of biological information beyond RNA abundance/expression.

**Figure 4:**
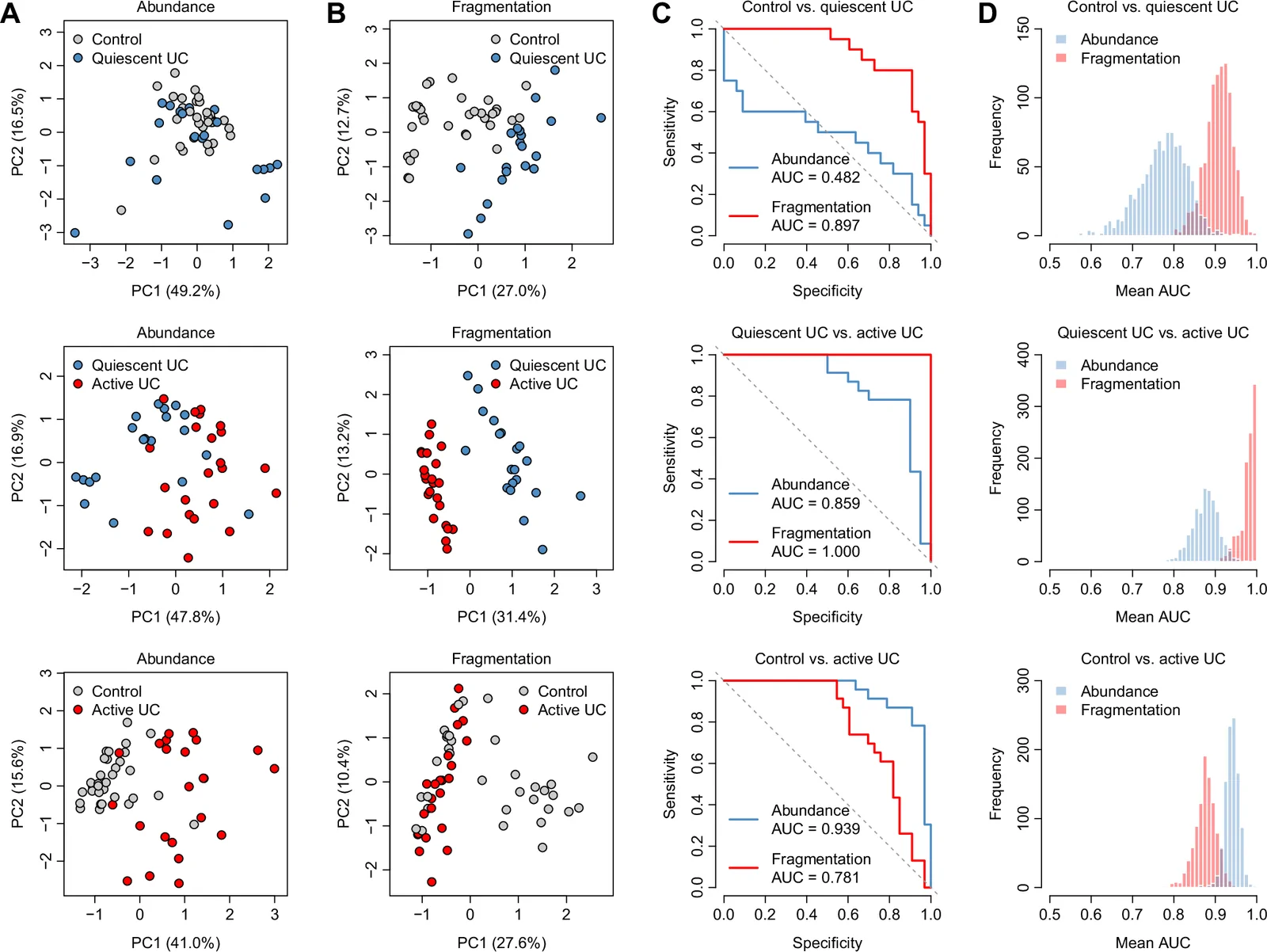
Classification power of sncRNA fragmentation in UC. (**A**) PCA on abundance of tsRNA/rsRNA subfamilies. (**B**) PCA on coverage pattern of tsRNA/rsRNA subfamilies. PC1 the first principal component, PC2 the second principal component. (**C**) ROC curves for the classification performance of PC1 between the individual groups. The blue curves indicate the classification performance of PC1 based on the abundance of tsRNA/rsRNA subfamilies, while the red ones indicate the classification performance of PC1 based on tRNA/rRNA fragmentation pattern. *DeLong*’s test was used to evaluate the difference in *AUC* between the blue and red ROC curves (*P* = 1.0 × 10^−10^ for classification of control *vs*. quiescent UC; *P* = 0.020 for classification of quiescent UC *vs*. active UC; *P* = 4.7 × 10^−3^ for classification of control *vs*. active UC). (**D**) Distribution of the mean *AUC* for the classification between individual groups. In total, 1000 rounds of fivefold cross-validation were performed. The blue histograms are the distributions of the mean *AUC* based on the abundance index, while the red ones are for the mean *AUC* based on the fragmentation index. Significant difference between mean *AUC* based on abundance index and mean *AUC* based on fragmentation index was observed for each classification (*Wilcoxon* rank-sum test: *P* = 2.8 × 10^−306^ for control *vs*. quiescent UC, *P* = 0 for quiescent UC *vs*. active UC, and *P* = 2.1 × 10^−305^ for control *vs*. active UC). Source data are available online for this figure.

**Figure EV4:**
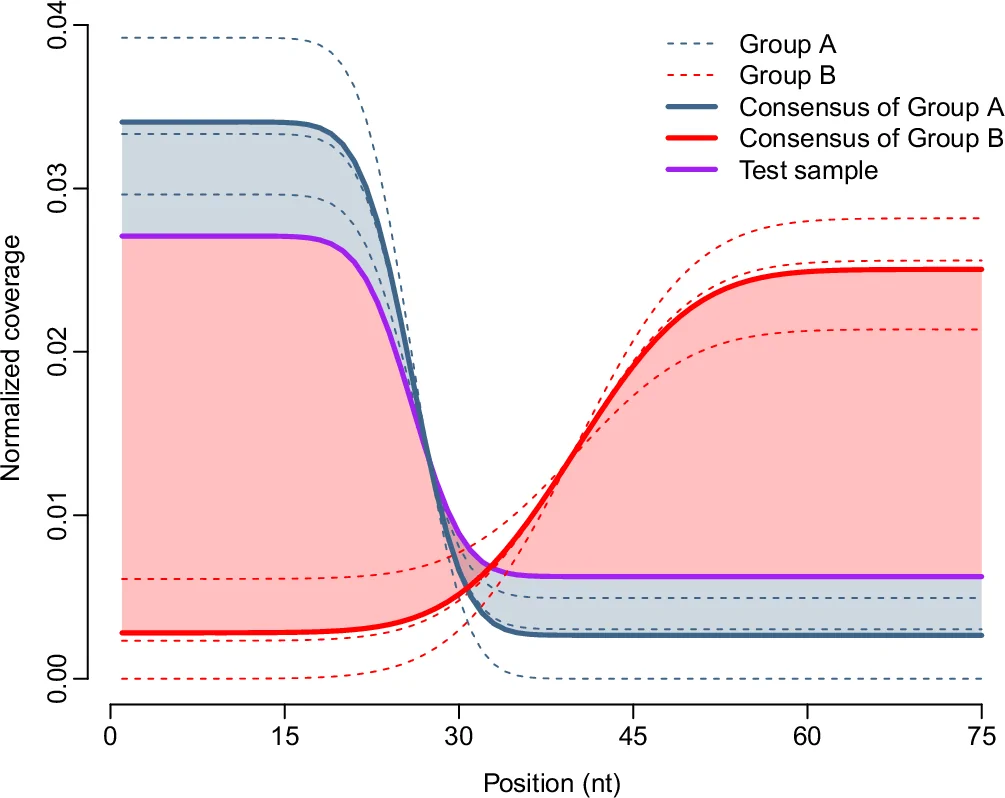
Schematic diagram of consensus coverage. We assume the fragmentation pattern of a given tRNA (with 75 nts in length) is analyzed for Group A (*n* = 3) and Group B (*n* = 3). The normalized coverage of tsRNAs is plotted along the parental tRNA for the individual samples within the two groups, illustrated as the blue/red dash curves. The solid blue and red curves represent the mean coverage pattern, i.e., “consensus coverage”, for Group A and Group B, respectively. For a given test sample with unknown classification information, the normalized coverage of tsRNAs is plotted along the parental tRNA as well, illustrated as the purple solid curve. The light blue area surrounded by the solid blue and solid purple curves represents the deviation between the consensus coverage of Group A and the coverage pattern of the test sample. Meanwhile, the pink area surrounded by the solid red and solid purple curves represents the deviation between the consensus coverage of Group B and the coverage pattern of the test sample.

We then systematically compared the classification power of these two regulatory layers. Relative to PCA on the abundance profile of tsRNA/rsRNA subfamilies, PCA on the fragmentation pattern indicates a better separation between controls and patients with quiescent UC and between quiescent and active UC patients, but not between controls and patients with active UC (Fig. 4A,B). The classification power of the first principal component (PC1) in Fig. 4A,B was evaluated using a receiver operating characteristic (ROC) curve. The area under the ROC curve (*AUC*) of fragmentation-based PC1 is significantly higher than that of abundance-based PC1 for the classifications of control *vs*. quiescent UC and quiescent UC *vs*. active UC, despite an inverse observation for the classification of control *vs*. active UC (Fig. 4C). To rigorously measure the classification performance of RNA abundance and RNA fragmentation, 1000 rounds of five-fold cross-validation were performed. During each round of cross-validation, “consensus abundance” and “consensus coverage” of the tsRNA/rsRNA subfamilies were inferred from the individual classification groups in the training data (see Methods for details). Subsequently, an abundance index and a fragmentation index were calculated for each sample within the test data based on the “consensus abundance” and “consensus coverage” (Fig. EV4), respectively (see “Methods” for details). Accordingly, a mean *AUC* was computed for each round of fivefold cross-validation. Fig. 4D demonstrated that the mean *AUC* based on fragmentation index was significantly higher than the mean *AUC* based on abundance index for the classifications of control *vs*. quiescent UC and quiescent UC *vs*. active UC, but not for the classification of control *vs*. active UC, which largely mirrors the findings of Fig. 4C. These results suggest that RNA fragmentation is not only an independent layer of information, but a more informative and sensitive indicator for disease classification. Fragmentation profiles therefore may provide complementary and superior diagnostic power relative to conventional abundance measures.

### A non-invasive RNA fragmentation signature identifies recurrent implantation failure

**Figure 5:**
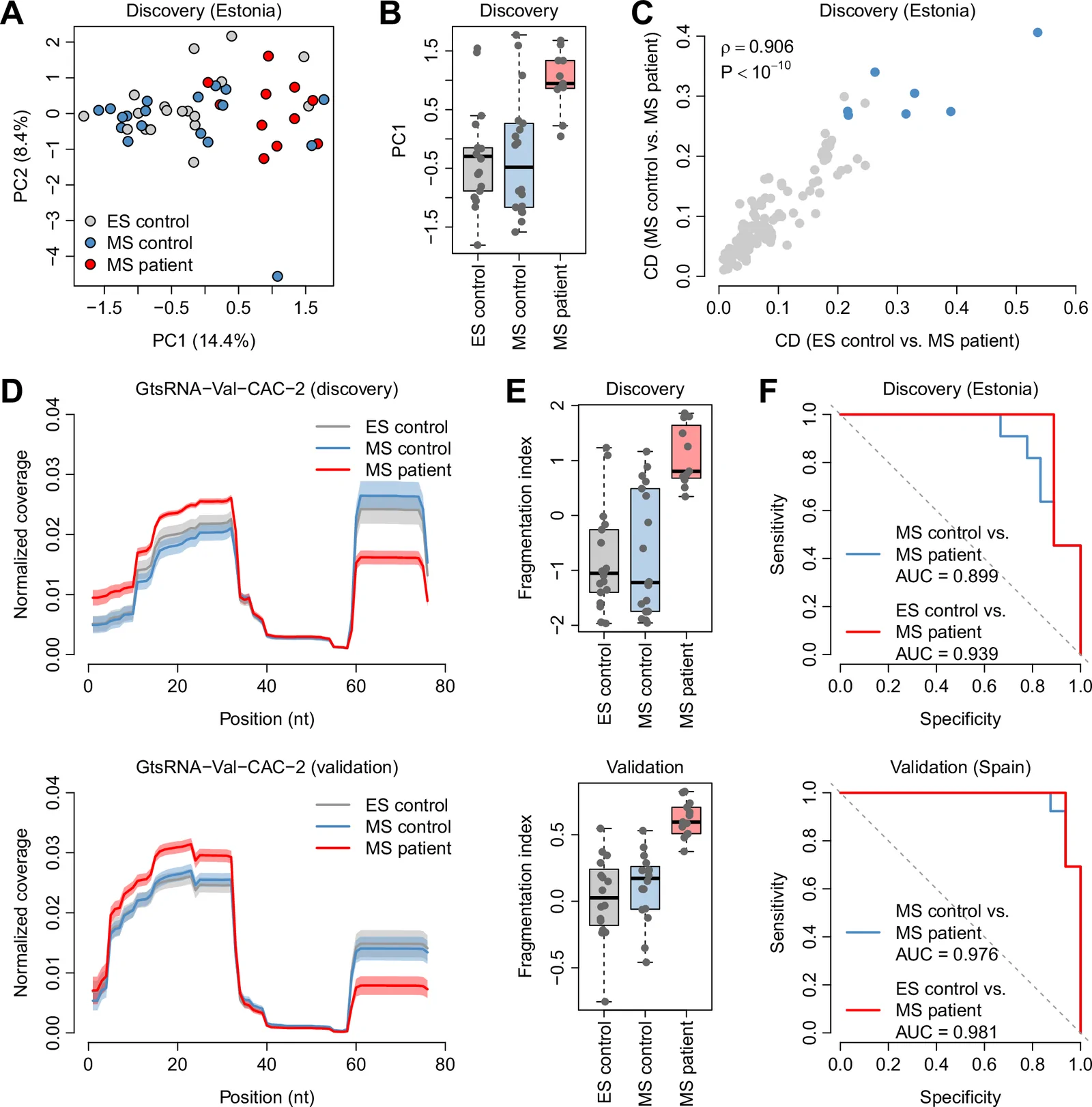
Blood RNA fragmentation signature of RIF. (**A**) PCA on tsRNA/rsRNA fragmentation in the ES controls (*n* = 18), MS controls (*n* = 18), and MS RIF patients (*n* = 11) from the discovery (Estonia) cohort. PC1: the first principal component; PC2: the second principal component. (**B**) Comparison in PC1 between the individual groups. In each box, the upper and lower bounds represent the 75th and 25th percentiles (*Q*_*3*_ and *Q*_*1*_), respectively. The upper and lower whiskers stand for the most extreme data points within *Q*_*3*_ + 1.5×(*Q*_*3*_–*Q*_1_) and *Q*_1_–1.5×(*Q*_3_–*Q*_1_), respectively. The median of the data points is indicated by the horizontal line inside the box. Significant difference in PC1 was observed between the ES controls and MS patients (*t-*test: *P* = 2.3 × 10^−5^) as well as between the MS controls and MS patients (*t-*test: *P* = 1.3 × 10^−4^). No significant difference in PC1 was observed between the ES and MS controls (*t-*test: *P* > 0.05). (**C**) Correlation in coverage difference (*CD*) in the discovery cohort. Each dot represents one parental tRNA/rRNA. The blue dots denote the RNA fragmentation signature. *X* axis: *CD* between the ES controls and MS RIF patients; *Y* axis: *CD* between the MS controls and MS RIF patients. The correlation coefficient (*ρ*) and *P* value were calculated using *Spearman*’s rank correlation test (*ρ* = 0.906 and *P* = 2.0 × 10^−63^). (**D**) Comparison in coverage pattern of GtsRNA-Val-CAC-2 between the ES controls, MS controls, and MS RIF patients in the discovery and validation (Spain) cohorts. (*n* = 16, 16, and 13 for the ES controls, MS controls, and MS patients in the validation cohort, respectively.) The solid curves indicate the mean normalized coverage along the parental tRNA, and the bands represent the SEM. (**E**) Comparison of the fragmentation index based on the RNA fragmentation signature between the individual groups in the discovery and validation cohorts. The definition of the box is the same as that of (**B**). A significant difference in fragmentation index was observed between the ES controls and MS patients (*t-*test: *P* = 1.7 × 10^−7^ for the discovery cohort; *P* = 3.0 × 10^−3^ for the validation cohort) as well as between the MS controls and MS patients (*t-*test: *P* = 5.8 × 10^−6^ for the discovery cohort; *P* = 7.3 × 10^−4^ for the validation cohort). (**F**) ROC curves for the classification performance of the fragmentation index between the individual groups in the discovery and validation cohorts. Source data are available online for this figure.

To assess whether RNA fragmentation profile could serve as a non-invasive biomarker for human diseases, we investigated a sncRNA-seq dataset (GEO accession: GSE108966) (Rekker et al, 2018), which includes peripheral whole blood samples from fertile women (controls) at early secretory (ES) and mid-secretory (MS) phases and patients with recurrent implantation failure (RIF) at MS phase. The RIF patients were characterized by having undergone at least three unsuccessful in vitro fertilization cycles with embryo transfers (Rekker et al, 2018). There were two independent cohorts in this dataset: the discovery cohort was from Estonia with 18 ES and 18 MS fertile controls and 11 MS RIF patients; the validation cohort was from Spain with 16 ES and 16 MS controls, and 13 MS patients. This two-cohort setting provided an opportunity to discover and then independently validate an RNA fragmentation-based biomarker. To avoid potential noise and improve statistical sensitivity, we focused on the tsRNA/rsRNA subfamilies with a mean *RPM* > 5. In the discovery cohort, PCA on sncRNA coverage pattern demonstrated a clear and significant separation between the control and RIF samples, whereas there was no significant difference in PC1 between the ES and MS control samples (Fig. 5A,B). This finding strongly suggests that the RNA fragmentation signature is specific to the RIF status, not simply a reflection of menstrual cycle timing.

**Figure EV5:**
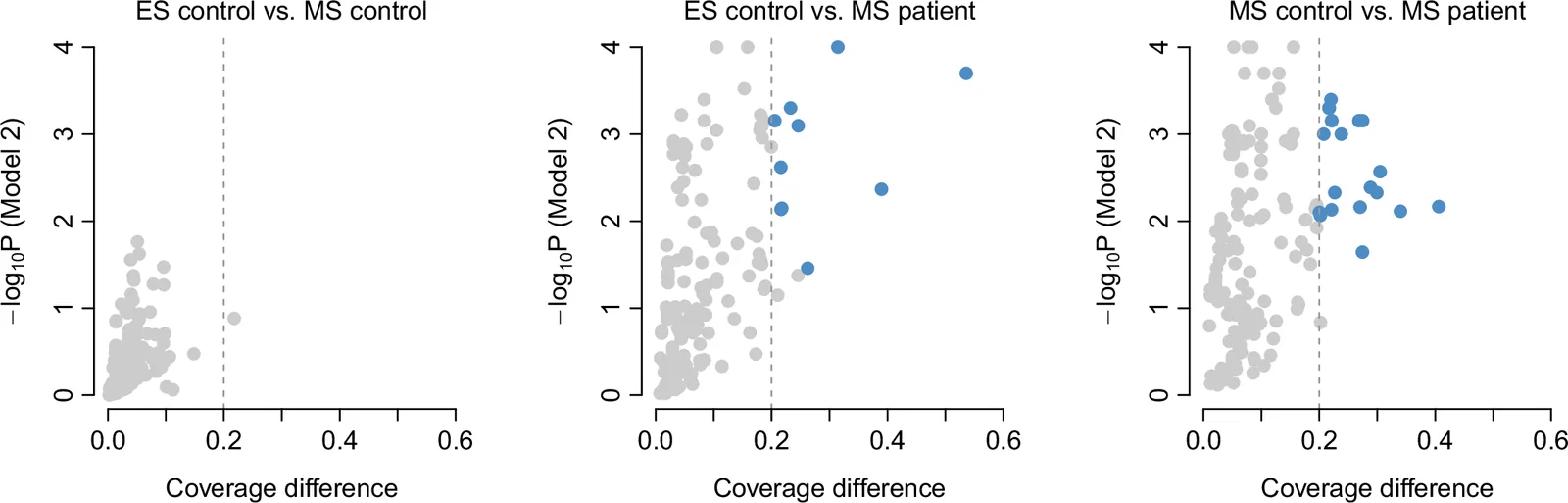
Relationship between coverage difference and statistical significance in RIF. Each dot represents one parental tRNA/rRNA. The blue dots denote the differentially fragmented parental RNAs. The *P* values illustrated by *Y* axis were calculated using *qMAP* Model 2. The data points with *P* = 0 were excluded from the scatter plots. The vertical line indicates the coverage difference level of 0.2.

To develop a diagnostic signature based on blood RNA fragmentation profile, we employed *qMAP* Model 2 to compare the sncRNA coverage pattern between the individual sample groups in the discovery cohort. In total, 0, 11, and 22 parental RNAs were found to be differentially fragmented between the ES and MS controls, between the ES controls and MS patients, and between MS controls and MS patients, respectively (Fig. EV5; Table EV3). A strong positive correlation in coverage difference was observed between the two control-patient comparisons (i.e., ES controls *vs*. MS patients and MS controls *vs*. MS patients) with seven parental RNAs (six tRNAs and one rRNA) showing a common significant differential fragmentation in both comparisons (Fig. 5C). These seven parental RNAs along with their fragmentation profiles were defined as a blood RNA fragmentation signature of RIF (Table EV4). Fig. 5D provides an exemplary coverage pattern of a sncRNA subfamily (GtsRNA-Val-CAC-2) within the signature, which indicates a clear deviation between the ES/MS controls and RIF patients as well as a consistent landscape shared by the ES and MS controls in both the discovery and validation cohorts. To assess the classification performance of the fragmentation signature, a fragmentation index was calculated for each sample within both the discovery and validation cohorts, which was based on the “consensus coverage” patterns of the seven signature sncRNA subfamilies “trained” among the MS controls and MS patients within the discovery cohort (see “Methods” for details). A higher fragmentation index indicates a higher RIF risk as well as a lower fertility likelihood. We found that the fragmentation index was significantly increased for the MS patients compared with the ES/MS controls in both the discovery and validation cohorts (Fig. 5E). Comparable ES and MS controls in both cohorts suggest menstrual phase does not confound the signature. Furthermore, the ROC curves plotted based on fragmentation index demonstrated an excellent classification performance in the two cohorts: *AUC* was 0.939 and 0.981 between the ES controls and MS patients in the discovery and validation cohorts, respectively; *AUC* was 0.899 and 0.976 between the MS controls and MS patients in the discovery and validation cohorts, respectively (Fig. 5F). These results suggest that blood RNA fragmentation patterns are stable, reproducible across cohorts, and can serve as high-performance, non-invasive biomarkers for RIF, which potentially opens the avenue of decoding RNA fragmentome for future precision medicine. Prospective studies with larger cohorts, standardized sample processing, and predefined decision thresholds will be required before diagnostic application.

### Aging-associated RNA fragmentation in sperm

We recently demonstrated an age-associated remodeling of tsRNA/rsRNA composition in mouse sperm and an aging-associated shift toward longer rsRNAs in mouse sperm heads (Shi et al, 2026).

**Figure 6:**
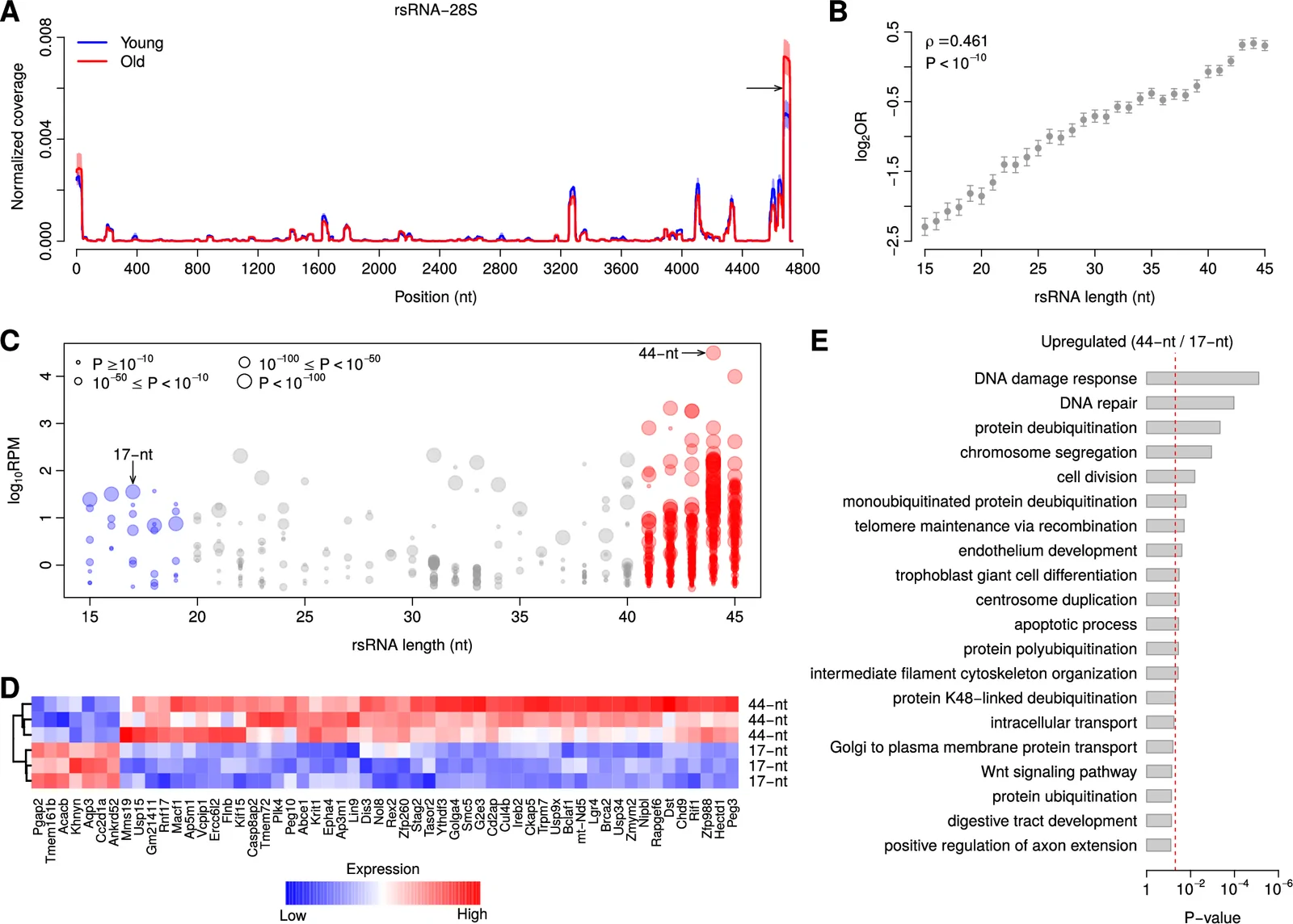
Aging-associated RNA fragmentation in mouse sperm heads. (**A**) Comparison in coverage pattern of rsRNA-28S between the young and old mice. The solid curves indicate the mean normalized coverage along 28S rRNA, and the bands represent the SEM. The arrow marks the dominant coverage peak, from which the candidate rsRNAs were prioritized. (**B**) Relationship between the length of rsRNA-28S and corresponding log_2_-transformed odds ratio (log_2_*OR*). The odds ratios were calculated using the *qMAP_mh* module, indicating the over-/under-representation status of the individual rsRNA species in old mice relative to young mice. Positive log₂*OR* indicates over-representation in aged mice relative to young mice, whereas negative log₂*OR* indicates under-representation. The dots and error bars stand for the means and 95% confidence intervals of log_2_*OR*, respectively (*n* = 658 ~ 1383 per category). The correlation coefficient (*ρ*) and *P* value were computed using *Spearman*’s rank correlation test (*ρ* = 0.461 and *P* = 0). (**C**) rsRNA species within the peak region highlighted in (**A**). Each dot represents one rsRNA species. The *Y* axis indicates mean abundance of the individual rsRNAs measured as log₁₀*RPM*. The dot size represents the statistical significance computed using *qMAP_mh*. The two arrows indicate the selected short (17-nt) and long (44-nt) rsRNA candidates used for the transfection experiment. (**D**) Expression heatmap of genes differentially expressed between mESCs transfected with the 17-nt and 44-nt rsRNA candidates. Three biological replicates were included for each transfection group. (**E**) Gene Ontology Biological Process terms associated with the genes upregulated in the 44-nt rsRNA-transfected mESCs relative to the 17-nt rsRNA-transfected mESCs. The *P* values were computed using *David* tool (modified one-tailed *Fisher*’s exact test). The vertical dashed line marks the significance level of *α* = 0.05. Source data are available online for this figure.

**Figure EV6:**
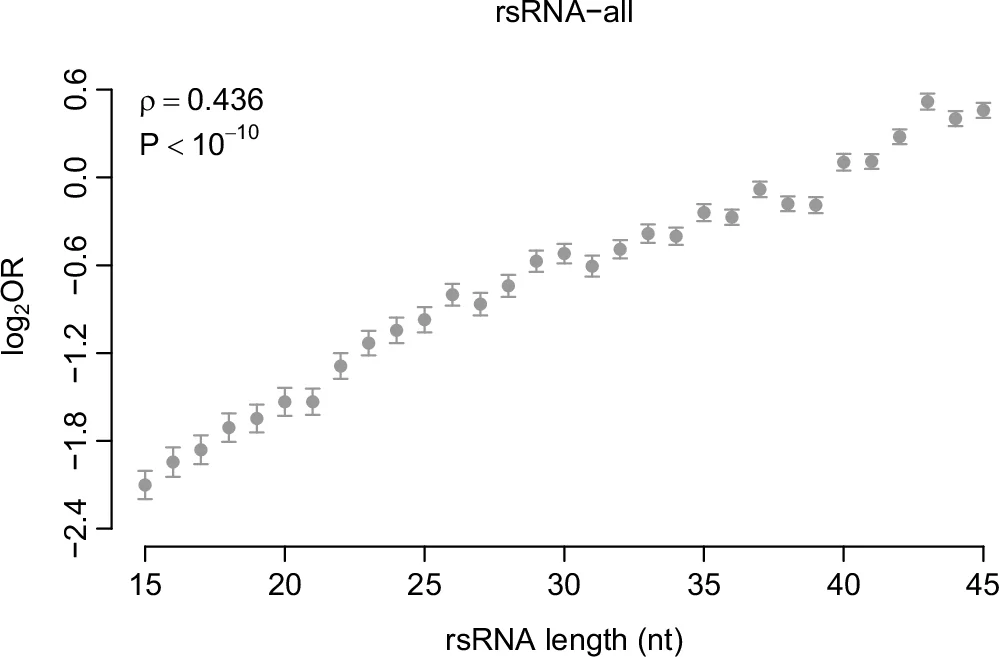
Relationship between the length of rsRNAs in mouse sperm heads and log_2_-transformed odds ratio (log_2_*OR*). The odds ratios were calculated using the *qMAP_mh* module, indicating the over-/under-representation status of the individual rsRNA species in old mice relative to young mice. The dots and error bars stand for the means and 95% confidence intervals of log_2_*OR*, respectively (*n* = 1070–2603 per category). The correlation coefficient (*ρ*) and *P* value were computed using *Spearman*’s rank correlation test (*ρ* = 0.436 and *P* = 0).

This dataset provided an opportunity to test whether *qMAP* can resolve a global rsRNA length shift into parental RNA-specific fragmentation events and prioritize the individual fragments responsible for these changes. To this end, we reanalyzed the sperm head PANDORA-seq data comparing young (10-week) and old (90-week) mice using *qMAP* Model 1. This analysis identified 116 differentially fragmented parental RNAs, with 28S rRNA ranking as the most significantly differentially fragmented parental RNA (Table EV5; Fig. 6A). We next applied the *qMAP_mh* module to screen the rsRNA species driving the observed differential fragmentation of 28S rRNA. Interestingly, the over-/under-representation level (relative to young mice) of the individual rsRNA-28S species, quantified by log₂-transformed odds ratio, was significantly and positively correlated with the length of these rsRNAs (Fig. 6B), which resonates with our previous finding that longer 28S-derived rsRNAs were preferentially enriched in aged sperm heads, whereas shorter species tended to be under-represented (Shi et al, 2026). A similar length-dependent pattern was also observed across all the rsRNA species within mouse sperm heads (Fig. EV6).

To test whether age-associated short and long rsRNAs could produce distinct cellular responses, we focused on the dominant coverage peak of rsRNA-28S highlighted in Fig. 6A to prioritize the candidate rsRNA species. The abundance of the individual rsRNA species falling into this peak region was shown in Fig. 6C. Among these rsRNAs, one short (17-nt) and one long (44-nt) rsRNA species (see Methods for details) were selected based on their statistical significance and relatively higher abundance (Fig. 6C). Notably, the 44-nt candidate contains the 17-nt sequence at its 3’ end, allowing comparison of nested fragments from the same 28S rRNA locus and thereby focusing on length-dependent effects. After transfection of these two rsRNAs into mouse embryonic stem cells (mESCs) respectively, poly(A)-enriched mRNA sequencing revealed distinct transcriptomic responses between the 17-nt and 44-nt rsRNA treatments (Fig. 6D). Gene ontology analysis of genes upregulated in the mESCs transfected with the 44-nt rsRNA suggests that longer rsRNAs induced a transcriptional program enriched for DNA damage response, cell division, telomere maintenance, and apoptosis-related biological processes in mESCs (Fig. 6E). Together, these results show that *qMAP* is able to resolve sperm-aging-associated rsRNA length shifts into specific fragment species and identify prominent individual rsRNAs whose length differences may lead to functional consequences, as supported by our proof-of-principle in vitro assay. Future work should further test whether endogenous RNA modifications and physiological stoichiometry impact such effects in vivo.

## Discussion

In the past few years, small RNA biology has undergone a rapid conceptual evolution (Shi et al, 2022). Advances in sequencing methods such as PANDORA-seq (Shi et al, 2025; Shi et al, 2021) and other related methods (Ferguson et al, 2025; Shi et al, 2022; Tosar et al, 2024; Watkins et al, 2022) by overcoming terminal and internal RNA modifications have revealed that various noncanonical sncRNAs, such as recently increasingly studied tsRNAs and rsRNAs, outnumber miRNAs and arise from regulated cleavage rather than random decay (Chen and Zhou, 2023; Jouravleva and Zamore, 2025). The observation that distinct fragments from the same parental RNA can exert different structural and regulatory effects implies that where an RNA is cleaved is as important as whether it is expressed (Kuhle et al, 2023), which motivates recognition of RNA cleavage patterns as a previously unquantified regulatory layer. Here, with our development of *qMAP*, we provided a computational solution to decode this “RNA fragmentome”. By quantifying normalized cleavage distributions along parental RNAs and identifying condition-specific shifts, *qMAP* measures a dimension of gene regulation that traditional abundance-based analyses overlook.

Across distinct biological and clinical contexts, *qMAP* revealed the breadth and significance of RNA fragmentation as a regulatory dimension. During cell reprogramming, RNA fragmentation patterns were dynamically remodeled with minimal changes in overall abundance (Fig. 2), indicating dedicated regulation during developmental transitions. In UC, RNA fragmentation patterns are more sensitive to pathological states than RNA abundance profiles (Figs. 3 and 4). Also, a compact fragmentation signature in circulation accurately identified RIF across independent human cohorts (Fig. 5), demonstrating stability, reproducibility, and translational potential. In sperm aging, *qMAP* recapitulated the previously observed rsRNA length shift from the perspective of rRNA-centered fragmentation remodeling and prioritized short and long rsRNAs with distinct effects on programming mESC gene expression (Fig. 6). These examples show that the “RNA fragmentome” can provide both molecular signatures and mechanistic candidates across cells, tissues, biofluids, and germ cells.

Beyond classification potential, biologically, selective RNA fragmentation represents a rapid post-transcriptional response system. Parental RNAs such as tRNAs and rRNAs exist in extraordinarily high copy numbers and are essential for translation and ribosome function. Cleaving these abundant RNAs into functional fragments offers a fast adaptive mechanism that does not require new transcription, enabling cells to quickly rewire regulatory pathways during stress, differentiation, or immune activation (Kuhle et al, 2023). Many tsRNAs and rsRNAs function through structure- and modification-dependent interactions—often independent of Argonaute—suggesting evolutionary origins predating RNA interference (RNAi) (Chen et al, 2021). In fact, beyond the canonical complementarity-based targeting mode of RNAi, many tsRNAs/rsRNAs can function in an aptamer-like fashion, utilizing their specific 3D structures—often modulated by RNA modifications—to bind proteins or other molecules, independent of linear sequence complementarity (Chen and Zhou, 2023; Kuhle et al, 2023). The specific cleavage sites generating a fragment directly influence its potential structure, length, and modification retention, thereby dictating its functional potential—whether it binds AGO for RNAi, interacts with ribosomes, sequesters proteins like cytochrome c, or forms higher-order structures like G-quadruplexes (Chen and Zhou, 2023; Kuhle et al, 2023). These fragments may even participate in RNA/ribonucleoprotein-based structural memory propagation to convey cell phenotype when properly incorporated in biomolecular condensates, as recently proposed (Cai et al, 2025). In other words, selective fragmentation determines functional fate.

*qMAP* provides a gateway to study pathological RNA cleavage as well as providing mechanistic insights to probe sncRNA distinct functions. In autoimmunity, for example, aberrant RNA processing may generate fragments that preferentially activate TLR7/8, amplifying inflammatory cycles (Yu et al, 2025). Distinguishing the pathogenic fragments from benign species requires exactly the type of high-resolution differential fragmentation quantification enabled by *qMAP*. Looking ahead, *qMAP* establishes a foundation for systematic exploration of the RNA fragmentome. Future developments could incorporate single-cell sncRNA coverage profiling (Hagemann-Jensen et al, 2018), cleavage motifs and secondary structure models (Holmes et al, 2023), site-specific RNA modification maps (Lucas et al, 2024; Yuan et al, 2024), and machine learning to predict functional fragment classes (Kuhle et al, 2023) along with integrating related databases (Guan and Grigoriev, 2023; Guan et al, 2025; Lei et al, 2023; Sordyl et al, 2025; Wang et al, 2022), leading to clinical-scale pipelines for non-invasive diagnostics. Eventually, an RNA Fragmentome Atlas across tissues, developmental stages, and disease states would enable the research community to define conserved and context-specific fragmentation codes.

Methodologically, *qMAP* offers flexibility via two core models. Model 1 uses a linear model suitable for low-replicate studies (e.g., 3 *vs*. 3), while the faster, non-parametric Model 2 is better suited for larger datasets. Both models rely on permutations, which can be computationally intensive for large analyses or long parental RNAs. Users can mitigate this by applying read count thresholds to filter out low-abundance species to reduce computational load. The downstream *qMAP_mh* module then aids in identifying the specific fragments driving differential patterns, prioritizing candidates for functional validation.

**Figure EV7:**
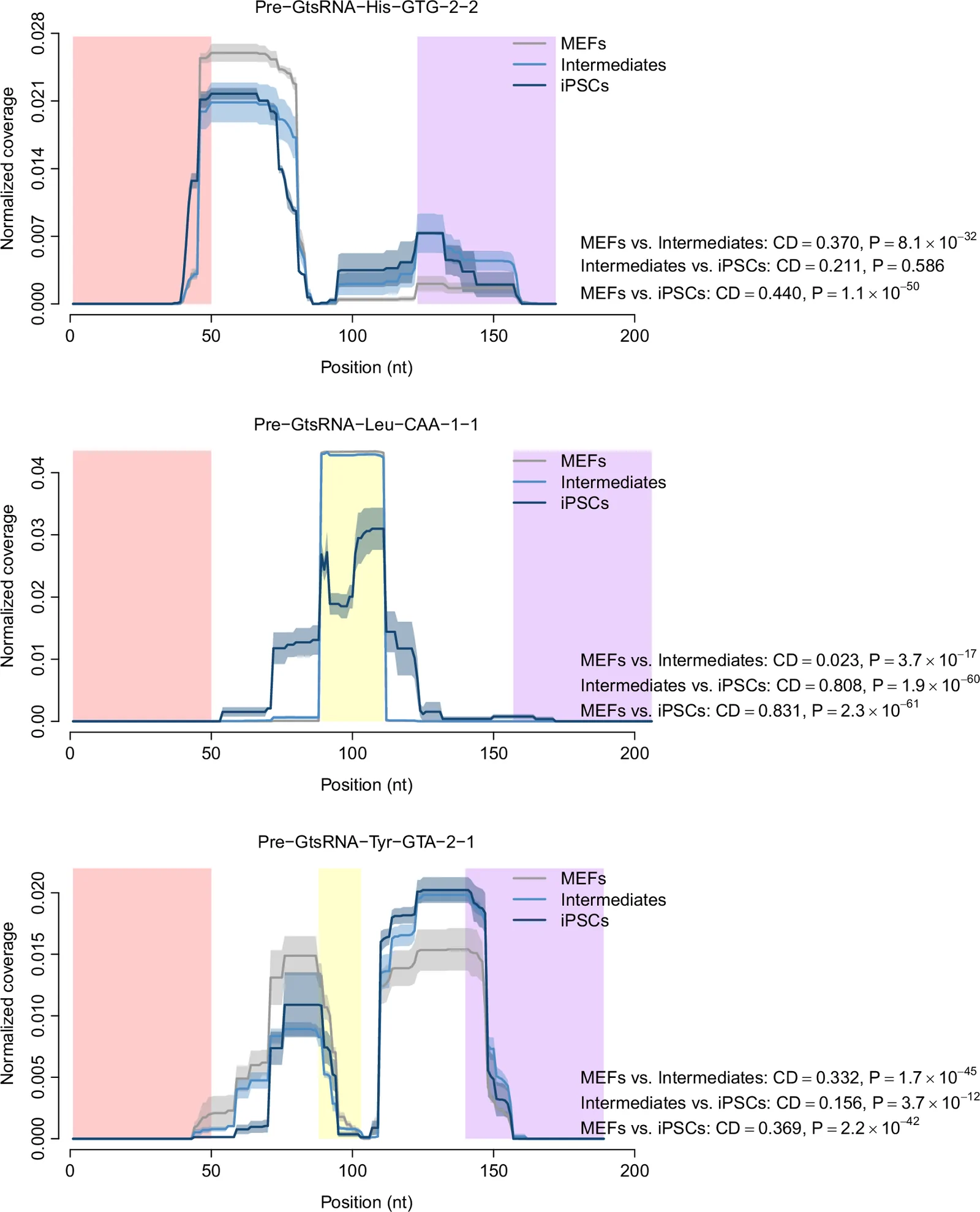
Comparison in coverage pattern of pre-GtsRNA-His-GTG-2-2, pre-GtsRNA-Leu-CAA-1-1, and pre-GtsRNA-Tyr-GTA-2-1 between MEFs, intermediates, and iPSCs. The solid curves indicate the mean normalized coverage along the pre-tRNAs, and the bands represent the SEM. The vertical pink, yellow, and purple bars indicate the 5’ leader, intron, and 3’ trailer of pre-tRNAs, respectively. The coverage difference (*CD*) and *P* value between the cell stages were calculated using *qMAP* Model 1.

The power of *qMAP* extends far beyond the tsRNA and rsRNA examples highlighted in this manuscript. The tool inherently supports fragmentation pattern analysis for any annotated RNA biotype, including snoRNAs, snRNAs, mRNAs, and Y RNAs, as long as the annotation information (e.g., from *SPORTS* (Shi et al, 2018)) is available. Because *qMAP* operates on user-defined parental RNA templates, it is also able to identify the difference in fragmentation for the reads derived from the precursors of parental RNAs. Some tsRNAs (named as tRFs or fitRNAs in these original studies) have been reported to be derived from 3’ trailers and introns of pre-tRNAs with function (Lee et al, 2009; Nostramo et al, 2025). As an example, *qMAP* detected differential fragmentation of pre-GtsRNA-His-GTG-2-2, pre-GtsRNA-Leu-CAA-1-1, and pre-GtsRNA-Tyr-GTA-2-1 during cell reprogramming from MEFs to intermediates and iPSCs (Fig. EV7).

One limitation shared by *qMAP* and other short-read sncRNA-seq analyses is the ambiguity of parental RNA assignment for fragments derived from highly conserved or repetitive RNA families, especially tRNAs. Many tRNA-derived fragments are identical across multiple tRNA genes or isodecoders, making their true parental origin impossible to determine from the fragment sequence alone. *qMAP* does not attempt to assign such fragments to a single unique parental origin. Instead, for each parental RNA template, normalized coverage was calculated using all annotated sncRNA species compatible with that template, thus these profiles should be interpreted as template-compatible fragmentation patterns rather than definitive locus- or isodecoder-specific cleavage maps. Alternative strategies, such as discarding multi-mapped reads, randomly assigning them, or fractionally distributing them across compatible templates, also introduce distinct biases. This ambiguity is therefore an inherent limitation of short-read fragment mapping, not specific to *qMAP*. Future integration of longer reads, genetic variants, tRNA modification profiles, precursor-specific features, or orthogonal perturbation experiments may help resolve parental origin in selected cases.

In summary, *qMAP* fills a critical methodological gap and reveals the RNA fragmentome as a dynamic and functional dimension of post-transcriptional regulation. Decoding this layer offers a path toward mechanistic insights into sncRNA biology and accelerates the development of RNA fragmentation-based diagnostics in human health care.

## Methods

**Table:** Reagents and tools table

Reagent/resource	Reference or source	Identifier or catalog number
**Experimental models**		
Mouse embryonic stem cell (v6.5)	Dr. Sihem Cheloufi Lab	
**Recombinant DNA**		
**Antibodies**		
**Oligonucleotides and other sequence-based reagents**		
rsRNA-28S 17-nt: 5’/Phos/rCrUrCrGrArCrArCrArArGrGrGrUrUrUrG-2'3 c-Phos	ChemGene	Customer service, Lot#: L193598-3
rsRNA-28S 44-nt: 5’/Phos/rCrUrCrGrCrUrGrCrGrArUrCrUrArUrUrGrArArArGrUrCrArGrCrCrCrUrCrGrArCrArCrArArGrGrGrUrU rUrG-2'3 c-Phos	ChemGene	Customer service, Lot#: L193598-2
**Chemicals, enzymes, and other reagents**		
TRIzol Reagent	Invitrogen	15596018
Isopropanol	Sigma-Aldrich	34863-1 L
Chloroform	Sigma-Aldrich	319988-1 L
Ethyl alcohol	Sigma-Aldrich	E7023-1L
RNA Clean & Concentrator Kit	ZYMO Research	R1017
Lipofectamine™ Stem	Invitrogen	STEM00003
Knock-out DMEM	Gibco	10829
Fetal Bovine Serum (FBS)	Gibco	10437; Lot-2190737RP
GlutaMAX Supplement	Gibco	35050061
100 X Streptomycin solution	Gibco	15140
Non-essential amino acids	Gibco	11140
2-Mercaptoethanol	Gibco	21985
ESGRO® Recombinant Mouse LIF Protein	Millipore	ESG1106
**Software**		
*Cutadapt*	https://cutadapt.readthedocs.io	
*SPORTS*	https://github.com/junchaoshi/sports1.1	
*kallisto*	https://github.com/pachterlab/kallisto	
*edgeR*	https://bioconductor.org/packages/release/bioc/html/edgeR.html	
*DAVID*	https://davidbioinformatics.nih.gov	
R	https://www.r-project.org	
Illustrator	Adobe Inc.	
**Other**		

### Normalized coverage

For a given parental RNA with a length of *l* nts, we collect all the sequencing reads (i.e., sncRNAs) that can be mapped to this parental RNA. The coverage level at base position *i* (*x*_*i*_) of the parental RNA can be expressed as the total count of reads covering this specific locus. Accordingly, the normalized coverage level (*e*_*i*_) can be calculated as:

$${e}_{i}={x}_{i}/{\sum }_{k=1}^{l}{x}_{k}$$

This normalized coverage is independent of the abundance level of the individual sncRNAs, and subsequently the area under the coverage curve is one:

$${\sum }_{i=1}^{l}{e}_{i}=1$$

### *qMAP* model 1

Model 1 is designed to identify differentially fragmented parental RNAs between groups using a linear model and random permutations. We first assume we have a sncRNA-seq dataset containing *m* controls (*a*_*1*_, *a*_*2*_, …, *a*_*m*_) without a specific condition (Group A) and *n* cases (*b*_*1*_, *b*_*2*_, …, *b*_*n*_) with the condition (Group B). For a parental RNA with *l* nts, we first calculate the normalized coverage for all the loci. For a given base position *i*, we have the normalized coverage across all the samples, which can be expressed as ${E}_{i}^{A}=\{{e}_{i}^{{a}_{1}},\,{e}_{i}^{{a}_{2}},\ldots ,{e}_{i}^{{a}_{m}}\}$ for Group A and ${E}_{i}^{B}=\{{e}_{i}^{{b}_{1}},\,{e}_{i}^{{b}_{2}},\ldots ,{e}_{i}^{{b}_{n}}\}$ for Group B. The statistical difference of normalized coverage at this position between the two groups can be measured by the *t*-statistic as:

$${t}_{i}=(\bar{{E}_{i}^{A}}-\bar{{E}_{i}^{B}})/\sqrt{\frac{{S}_{A}^{2}}{m}+\frac{{S}_{B}^{2}}{n}}$$

where $\bar{{E}_{i}^{A}}$ and $\bar{{E}_{i}^{B}}$ are the mean of ${E}_{i}^{A}$ and ${E}_{i}^{B}$, respectively, while *S*_*A*_ and *S*_*B*_ are the standard deviation of ${E}_{i}^{A}$ and ${E}_{i}^{B}$, respectively. Accordingly, a vector containing the absolute values of the *t*-statistic across all the loci along the parental RNA can be composed as ${T}^{R}=\{\left|{t}_{1}^{R}\right|,\,\left|{t}_{2}^{R}\right|,\ldots ,\left|{t}_{l}^{R}\right|\}$. Here, the superscript “*R*” represents that the *t*-statistic values are calculated based on the real sample grouping. We next perform *z* rounds of random permutations by shuffling the samples between Group A and Group B. The absolute values of the *t*-statistic based on the permutated grouping can be expressed as ${T}^{P}=\{\left|{t}_{1}^{{P}_{1}}\right|,\,\left|{t}_{2}^{{P}_{1}}\right|,\ldots ,\left|{t}_{l}^{{P}_{1}}\right|,\ldots ,\left|{t}_{1}^{{P}_{z}}\right|,\,\left|{t}_{2}^{{P}_{z}}\right|,\ldots ,\left|{t}_{l}^{{P}_{z}}\right|\}$. Accordingly, a larger vector combining *T*^*R*^ and *T*^*P*^ is composed as $T={T}^{R}\cup {T}^{P}$. A linear model comparing the normalized coverage between the two groups can be constructed as $T={{\beta }_{0}+\beta }_{1}C+{\beta }_{2}{L}_{2}+{\beta }_{3}{L}_{3}+\ldots +{\beta }_{l}{L}_{l}$, where *C* is a binary vector containing the categorical information for each data point in *T* (i.e., real *t*-statistic based on original grouping *vs*. permutated *t*-statistic based on shuffled grouping), while *L*_*i*_ is a binary vector defining locus *i* in the parental RNA for each data point in *T*. Therefore, the differential fragmentation of the given parental RNA can be statistically evaluated by the one-sided *P* value of the variable *C*, which measures the probability that the observed difference arises by chance. The overall difference in normalized coverage between Group A and Group B can be calculated as:

$$d={\sum }_{i=1}^{l}\left|\bar{{e}_{i}^{A}}-\bar{{e}_{i}^{B}}\right|$$

where *d* is the overall difference in normalized coverage and $\left|\bar{{e}_{i}^{A}}-\bar{{e}_{i}^{B}}\right|$ is the absolute value of the difference in mean normalized coverage at base position *i* between the two groups. Subsequently, differential fragmentation of the parental RNA can be determined based on the computed *P* value and coverage difference, which explores whether the fragmentation pattern is significantly changed between Group A and Group B, and the alteration is large enough in magnitude to be biologically meaningful.

### *qMAP* model 2

Model 2 is based on a permutation test. Similar to Model 1, we assume there are two groups (Group A and Group B) in a sncRNA-seq dataset. For a parental RNA, we calculate the overall difference in normalized coverage between the two groups as described above and save the value as *d*^*R*^. We next perform *z* rounds of random permutations by shuffling the samples between Group A and Group B. The overall difference in normalized coverage based on the permutated grouping can be expressed as ${D}^{P}=\{{d}_{1}^{P},{d}_{2}^{P},\ldots ,{d}_{z}^{P}\}$. Our alternative hypothesis is that *d*^*R*^ is larger than expected by chance if there is a significant difference in RNA fragmentation pattern between Group A and Group B. An empirical *P* value can be calculated as: P=y/z, where *y* is the cardinality of $\left\{d\in {D}^{P}{|d}\ge {d}^{R}\right\}$.

### *qMAP_mh* module

*qMAP_mh* is designed to identify the sncRNA species that are involved in the observed differential fragmentation in a parental RNA. We assume we have a sncRNA-seq dataset containing *m* controls (Group A) and *n* cases (Group B) and we have already observed a differential fragmentation in a given parental RNA. The sncRNAs over- or under-represented in either the controls or the cases are considered to contribute to the differential fragmentation. For a given sncRNA species that can be mapped to the parental RNA, we construct a 2×2 contingency matrix based on the sequencing reads from a selected control sample and a selected case sample as $$\left[\begin{array}{cc}{f}^{B} & {g}^{B}\\ {f}^{A} & {g}^{A}\end{array}\right]$$, where ${f}^{A}$ is the count of reads of this sncRNA in Group A, ${g}^{A}$ is the count of reads of the other sncRNAs that can be mapped to the parental RNA in Group A, ${f}^{B}$ is the count of reads of this sncRNA in Group B, and ${g}^{B}$ is the count of reads of the other sncRNAs that can be mapped to the parental RNA in Group B. As we have *m* controls and *n* cases, we can construct *m*×*n* contingency matrices. We next combine all these contingency matrices using the Mantel–Haenszel procedure with continuity correction (Mantel and Haenszel, 1959). A Mantel–Haenszel statistic can be calculated as:

$${X}^{2}=\frac{{\left\{\left|{\sum }_{i=1}^{w}\left[{f}_{i}^{B}-({f}_{i}^{B}+{g}_{i}^{B})({f}_{i}^{B}+{f}_{i}^{A})/{s}_{i}\right]\right|-0.5\right\}}^{2}}{{\sum }_{i=1}^{w}({f}_{i}^{A}+{g}_{i}^{A})({f}_{i}^{B}+{g}_{i}^{B})({f}_{i}^{A}+{f}_{i}^{B})({g}_{i}^{A}+{g}_{i}^{B})/[{s}_{i}^{2}({s}_{i}-1)]}$$

where *X*^2^ is the Mantel–Haenszel statistic, *w* = *m*×*n*, and *s*_*i*_ is the sum of all the elements in contingency matrix *i*. A *P* value can be inferred from the Mantel–Haenszel statistic on a chi-square distribution. An overall odds ratio can be calculated as:

$${OR}=\frac{\mathop{\sum }_{i=1}^{w}{f}_{i}^{B}{g}_{i}^{A}/{s}_{i}}{\mathop{\sum }_{i=1}^{w}{f}_{i}^{A}{g}_{i}^{B}/{s}_{i}}$$

where *OR* is the overall odds ratio. *OR* > 1 suggests an over-representation of the sncRNA in the cases, while *OR* < 1 suggests an over-representation of the sncRNA in the controls.

### Abundance index

Abundance index (*I*_*abun*_) is computed based on the abundance data of the selected sncRNA subfamilies. For a given sncRNA subfamily, the abundance is normalized by the mean and standard deviation of *RPM* of this sncRNA subfamily across all the samples as:

$$a=({RPM}-\bar{{RPM}})/{S}_{{RPM}}$$

where *a* is the normalized abundance, and $\bar{{RPM}}$ and *S*_*PRM*_ are the mean and standard deviation of *RPM*, respectively. For a classification between two groups (Group A and Group B), the mean normalized abundance (named “consensus abundance” in this study) can be calculated for Group A (*a*^*A*^) and Group B (*a*^*B*^), respectively. The abundance index based on the selected sncRNA subfamilies can be expressed as:

$${I}_{{abun}}=\mathop{\sum }_{i=1}^{q}(\left|{a}_{i}-{a}_{i}^{A}\right|-\left|{a}_{i}-{a}_{i}^{B}\right|)$$

where *a*_*i*_ is the normalized abundance of sncRNA subfamily *i*, ${a}_{i}^{A}$ and ${a}_{i}^{B}$ are the consensus abundance of sncRNA subfamily *i* of Group A and Group B, respectively, and *q* is the number of selected sncRNA subfamilies. A higher abundance index suggests a higher classification likelihood of Group B as well as a lower likelihood of Group A.

### Fragmentation index

Fragmentation index (*I*_*frag*_) is computed based on the normalized coverage of the selected sncRNA subfamilies. For a given sncRNA subfamily, we assume the length of the parental RNA is *l* nts. For a classification between two groups (Group A and Group B), the mean coverage along the parental RNA (named “consensus coverage” in this study) can be expressed as ${E}^{A}=\{{e}_{1}^{A},\,{e}_{2}^{A},\ldots ,{e}_{l}^{A}\}$ for Group A and ${E}^{B}=\{{e}_{1}^{B},\,{e}_{2}^{B},\ldots ,{e}_{l}^{B}\}$ for Group B. For a given sample with a normalized coverage $E=\{{e}_{1},{e}_{2},\ldots ,{e}_{l}\}$, the deviation from the consensus coverage of Group A (*D*^*A*^) and Group B (*D*^*B*^) of this sample (Fig. EV4) can be calculated as:

$${D}^{A}=\left|E-{E}^{A}\right|=\mathop{\sum }_{k=1}^{l}\left|{e}_{k}-{e}_{k}^{A}\right|$$

$${D}^{B}=\left|E-{E}^{B}\right|={\sum }_{k=1}^{l}\left|{e}_{k}-{e}_{k}^{B}\right|$$

The fragmentation index based on the selected sncRNA subfamilies can be expressed as:

$${I}_{{frag}}={\sum }_{i=1}^{q}\left({D}_{i}^{A}-{D}_{i}^{B}\right)$$

where *q* is the number of selected sncRNA subfamilies. A higher fragmentation index suggests a higher classification likelihood of Group B as well as a lower likelihood of Group A.

### Public sncRNA-seq datasets

The performance of *qMAP* was evaluated using publicly available sncRNA-seq datasets from the GEO database (Edgar et al, 2002). The GEO dataset GSE144666 contains sncRNA-seq data (PANDORA-seq) from three distinct mouse cell types: mouse embryonic fibroblasts (day 0, MEFs), reprogramming intermediate cells (day 3, intermediates), and stably derived induced pluripotent stem cells (iPSCs) (Shi et al, 2021; Data ref: Shi et al, 2021). This dataset was used to investigate the dynamic change in sncRNAs during transcription factor-mediated somatic cell reprogramming to pluripotency. For each of the three stages (MEFs, Intermediates, and iPSCs), three biological replicates were included. The sequencing libraries were constructed using the NEBNext® Multiplex Small RNA Library Prep Set for Illumina. Another sncRNA-seq dataset, GSE185102, was derived from colon tissues of controls (*n* = 33) and patients with active ulcerative colitis (UC) (*n* = 23) or quiescent UC (*n* = 20) (Inciuraite et al, 2024; Data ref: Skieceviciene et al, 2023). The sequencing libraries were constructed using the TruSeq Small RNA Library Preparation Kit. In the GEO dataset GSE108966, sncRNA profiles in blood were compared between the early secretory (ES) and mid-secretory (MS) phases in fertile women, as well as the MS phase in patients with recurrent implantation failure (RIF) (Rekker et al, 2018; Data ref: Rekker et al, 2019), to identify sncRNA subfamilies exhibiting differential coverage patterns. There are two cohorts from Estonia and Spain, respectively, within this dataset. The Estonia cohort, comprising 18 ES and 18 MS control samples from fertile women and 11 case samples (MS phase from RIF patients), was designated as the discovery cohort. The Spain cohort, consisting of 16 ES control samples, 16 MS control samples, and 13 MS patient samples, served as the validation cohort. The sequencing libraries were prepared from blood samples using the TruSeq Small RNA Library Preparation Kit. Aging-associated sperm RNA fragmentation was tested using the GEO dataset GSE256182 (Shi et al, 2026; Data ref: Shi et al, 2025). Here, we investigated the sperm head PANDORA-seq data from four 10-week-old and three 90-week-old mice. These sequencing libraries were constructed using the NEBNext® Multiplex Small RNA Library Prep Set for Illumina.

### Processing of sncRNA-seq data

Adapter-trimming of the raw sequencing reads was performed using *Cutadapt* (Martin, 2011). The *SPORTS* tool was applied to perform sncRNA annotation and quantification (Shi et al, 2018), allowing a maximum mismatch of one. For downstream comparative analysis, a matrix containing the read counts for each annotated sncRNA species across all samples was generated and used.

### Pre-tRNA analysis

Pre-tRNA templates were generated by extending the corresponding tRNA gene loci with 50-nt of upstream genomic sequence and 50-nt of downstream genomic sequence to represent the 5’ leader and 3’ trailer, respectively. Annotated introns were retained where present. To avoid ambiguity between mature tRNA-derived and pre-tRNA-derived fragments, only sncRNA species that can be mapped to the pre-tRNA templates but not to the corresponding mature tRNA sequences were included in the pre-tRNA analysis.

### Differential fragmentation analysis using *qMAP*

The details regarding the sncRNA families/subfamilies and parental RNAs investigated in this study are listed in Tables EV1–EV3 and EV5. The performance of *qMAP* was tested using the public datasets mentioned above, allowing a maximum mismatch of one. For each parental RNA, the coverage pattern was calculated based on all the sncRNA species that can be mapped to the given RNA template. For each comparison, only the parental RNAs with mean mapped read counts across samples ≥10 were analyzed. Since there were more than two groups in most of our sncRNA-seq datasets, only the parental RNAs that showed up in all the possible comparisons were retained. For *qMAP* Model 1, 100 rounds of permutations were applied to identify differentially fragmented parental RNAs, while for *qMAP* Model 2, we applied 10,000 rounds of permutations. The *P* values calculated by both models were adjusted for multiple testing using the *Benjamini–Hochberg* procedure. The parental RNAs with adjusted *P* < 0.1 and coverage difference >0.2 were deemed differentially fragmented. To be noted, *qMAP* quantifies fragmentation patterns from the sncRNA species detected and annotated in each sequencing dataset. It does not model or correct for library-preparation biases caused by RNA modifications, adapter-ligation efficiency, reverse-transcription arrest, or amplification efficiency. Thus, reported fragmentation profiles represent the detectable sncRNA population under the sequencing protocol being used.

### Identifying over-/under-represented sncRNA species using *qMAP_mh*

*qMAP_mh* was applied to identify the sncRNA species that are involved in the observed differential fragmentation of the selected parental RNAs (i.e., GtRNA-Ser-AGA-1 and rRNA-28S) during iPSC induction, allowing a maximum mismatch of one. Only the sncRNA species with mean read counts across samples ≥5 were retained. The *P* values calculated by *qMAP_mh* were adjusted for multiple testing using the Benjamini–Hochberg procedure. The sncRNA species with adjusted *P* < 0.05 and log_2_-transformed odds ratio (log_2_*OR*) > 2 were considered as over-represented, while the sncRNA species with adjusted *P* < 0.05 and log_2_*OR* < -2 were considered as under-represented.

### tRNA secondary structure

We obtained the secondary structure information (e.g., GtRNA-Ser-AGA-1) from GtRNAdb (Chan and Lowe, 2009, 2016) and visualized the secondary structure using the *forna* tool (Kerpedjiev et al, 2015).

### mESC transcriptomic analysis

The transfection of 17-nt rsRNA (5’-CUCGACACAAGGGUUUG-3’) and 44-nt rsRNA (5’-CUCGCUGCGAUCUAUUGAAAGUCAGCCCUCGACACAAGGGUUUG-3’) in V6.5 mESCs was performed using the same protocol as described in our previous publication (Shi et al, 2026). The mESCs were routinely tested for mycoplasma contamination, which have been confirmed to be negative. The 17-nt and 44-nt rsRNAs were transfected at the same molar concentration of 200 nM. Upon 24 h after rsRNA transfection, total RNA from the individual mESC samples was extracted using TRIzol and subjected to poly(A)-enriched library construction and sequencing. The sample size was determined based on our previous publication (*n* = 3 for each transfection group) (Shi et al, 2021). The *kallisto* tool (Bray et al, 2016) was applied to summarize the genome-wide gene expression based on GRCm39 genome assembly. The *edgeR* tool (Robinson et al, 2010) was used to identify the differentially expressed genes with the *TMM* method for data normalization and the likelihood ratio test for differential expression estimation. The genes with a false discovery rate <0.1 were deemed differentially expressed. Gene ontology analysis on the differentially expressed genes was performed using the *DAVID* tool (Sherman et al, 2022). The mRNA sequencing data have been deposited in the GEO database (GSE330870).

### Statistical analysis

We performed statistical analysis in this study using R programming. In brief, *Spearman*’s rank correlation test, *t-*test, *Wilcoxon* rank-sum test, *P* value adjustment, PCA were conducted using the “cor.test”, “t.test”, “wilcox.test”, “p.adjust”, and “dudi.pca” (*ade4* package) functions, respectively. The 95% confidence intervals were computed using the “CI” function within the *Rmisc* package. We applied the “roc” function (*pROC* package) to obtain the ROC curves and corresponding *AUC* levels for the classification analysis. The “roc.test” function (*pROC* package) was used to perform *DeLong*’s test between ROC curves. The “shapiro.test” function was used to check data normality. No blinding was performed in this study.

## Supplementary information

Table EV1

Table EV2

Table EV3

Table EV4

Table EV5

Peer Review File

Source data Fig. 2

Source data Fig. 3

Source data Fig. 4

Source data Fig. 5

Source data Fig. 6

Expanded View Figures

## Data Availability

The sequencing data and source code produced in this study are available in these repositories: The mESC mRNA sequencing data: Gene Expression Omnibus GSE330870. The source code of *qMAP*: GitHub (https://github.com/cozyrna/qMAP). The source data of this paper are collected in the following database record: biostudies:S-SCDT-10_1038-S44320-026-00237-2.
