# Supplementary material for: qMAP decodes RNA fragmentation dynamics in development and disease
Source: Mol Syst Biol. 2026 Jul 20;22(10):1626–49. doi: 10.1038/s44320-026-00237-2 (PMC13638987; doi:10.1038/s44320-026-00237-2)
Supplement: Supplementary file 1 [file 44320_2026_237_MOESM12_ESM.pdf]

Expanded View Figures

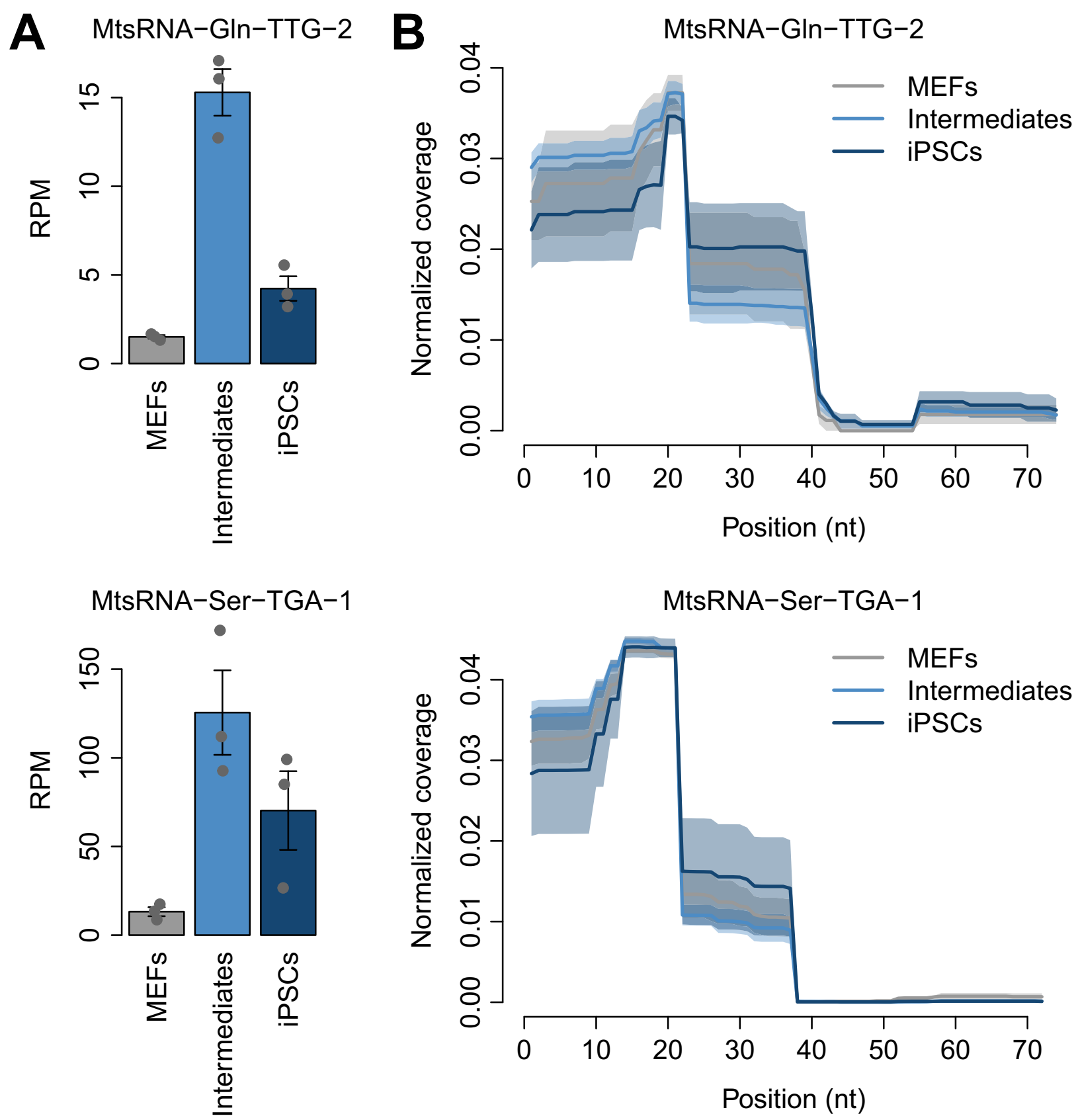

Figure EV1. Dynamic sncRNA abundance during iPSC induction.

(**A**) Comparison in abundance (*RPM*) of MtsRNA-Gln-TTG-2 and MtsRNA-Ser-TGA-1 between MEFs, intermediates, and iPSCs ($n = 3$ per group). The bars and the error bars represent the mean ± SEM. *t*-test was used to compare the difference in *RPM*: for MtsRNA-Gln-TTG-2, $P = 8.7 \times 10^{-3}$ between MEFs and intermediates, $P = 4.8 \times 10^{-3}$ between intermediates and iPSCs, and $P = 0.056$ between MEFs and iPSCs; for MtsRNA-Ser-TGA-1, $P = 0.041$ between MEFs and intermediates, $P = 0.165$ between intermediates and iPSCs, and $P = 0.122$ between MEFs and iPSCs. (**B**) Comparison in coverage pattern of MtsRNA-Gln-TTG-2 and MtsRNA-Ser-TGA-1 between MEFs, intermediates, and iPSCs. The solid curves indicate the mean normalized coverage along the parental tRNAs, and the bands represent the SEM. No significant difference in normalized coverage was observed between the individual cell stages (*qMAP* Model 1: $P > 0.05$).

© The Author(s)

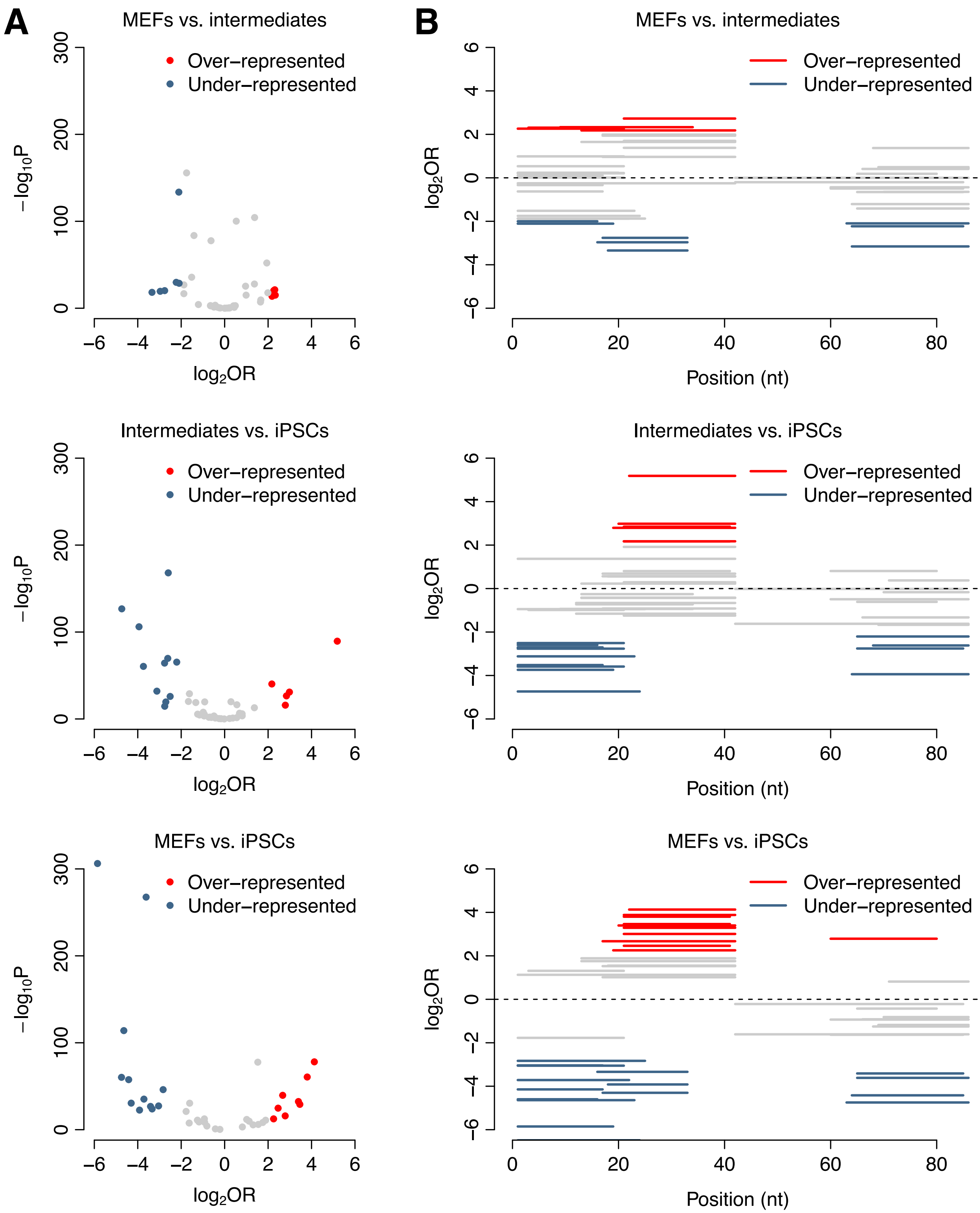
A
B
MEFs vs. intermediates
Over–represented
Under–represented
−log10P
log2OR
Position (nt)
Intermediates vs. iPSCs
MEFs vs. iPSCs

 © The Author(s)

Figure EV2. GtsRNA-Ser-AGA-1 species that are involved in differential fragmentation during iPSC induction.

(**A**) Volcano plots showing the over-/under-represented GtsRNA-Ser-AGA-1 species ($n = 3$ per group). *X* axis: $\log_2$-transformed odds ratio ($\log_2 OR$); *Y* axis: $\log_{10}$-transformed *P* value. The odds ratios and *P* values were calculated using the *qMAP_mh* module. The data points with *P* value = 0 were excluded from the volcano plots. The tsRNA species with adjusted $P < 0.05$ and $\log_2 OR > 2$ were considered as over-represented, while the tsRNA species with adjusted $P < 0.05$ and $\log_2 OR < -2$ were considered as under-represented. The baselines of the individual comparisons are MEFs, intermediates, and MEFs for the upper, middle, and lower panels, respectively. (**B**) Mapping patterns of the over-/under-represented tsRNA species along the parental tRNA.

© The Author(s)

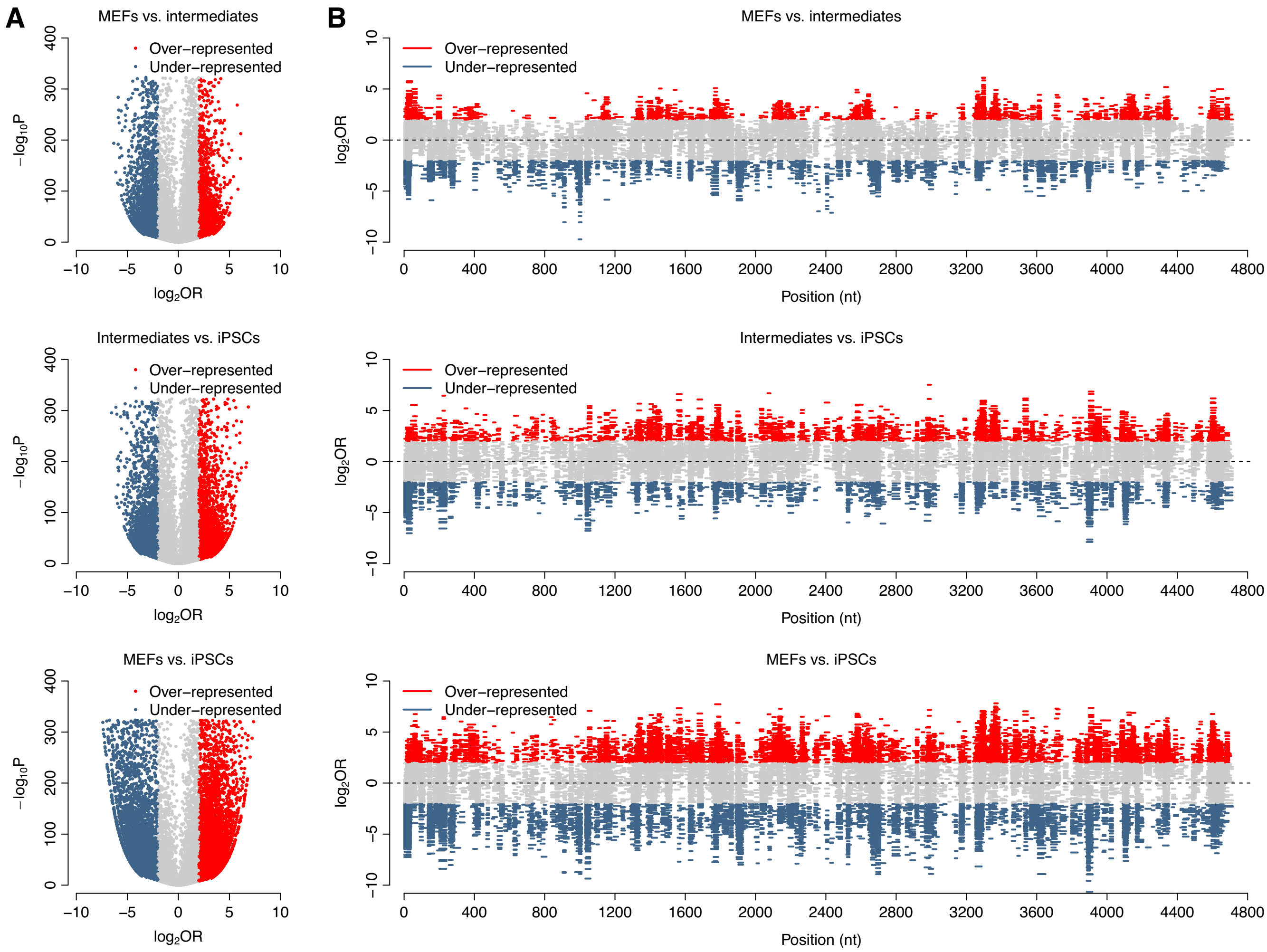

Figure EV3. rsRNA-28S species that are involved in differential fragmentation during iPSC induction.

(**A**) Volcano plots showing the over-/under-represented rsRNA-28S species ($n = 3$ per group). *X* axis: $\log_2$-transformed odds ratio ($\log_2 OR$); *Y* axis: $\log_{10}$-transformed *P* value. The odds ratios and *P* values were calculated using the *qMAP_mh* module. The data points with *P* value = 0 were excluded from the volcano plots. The rsRNA species with adjusted $P < 0.05$ and $\log_2 OR > 2$ were considered as over-represented, while the rsRNA species with adjusted $P < 0.05$ and $\log_2 OR < -2$ were considered as under-represented. The baselines of the individual comparisons are MEFs, intermediates, and MEFs for the upper, middle, and lower panels, respectively. (**B**) Mapping patterns of the over-/under-represented rsRNA species along the parental rRNA.

 © The Author(s)

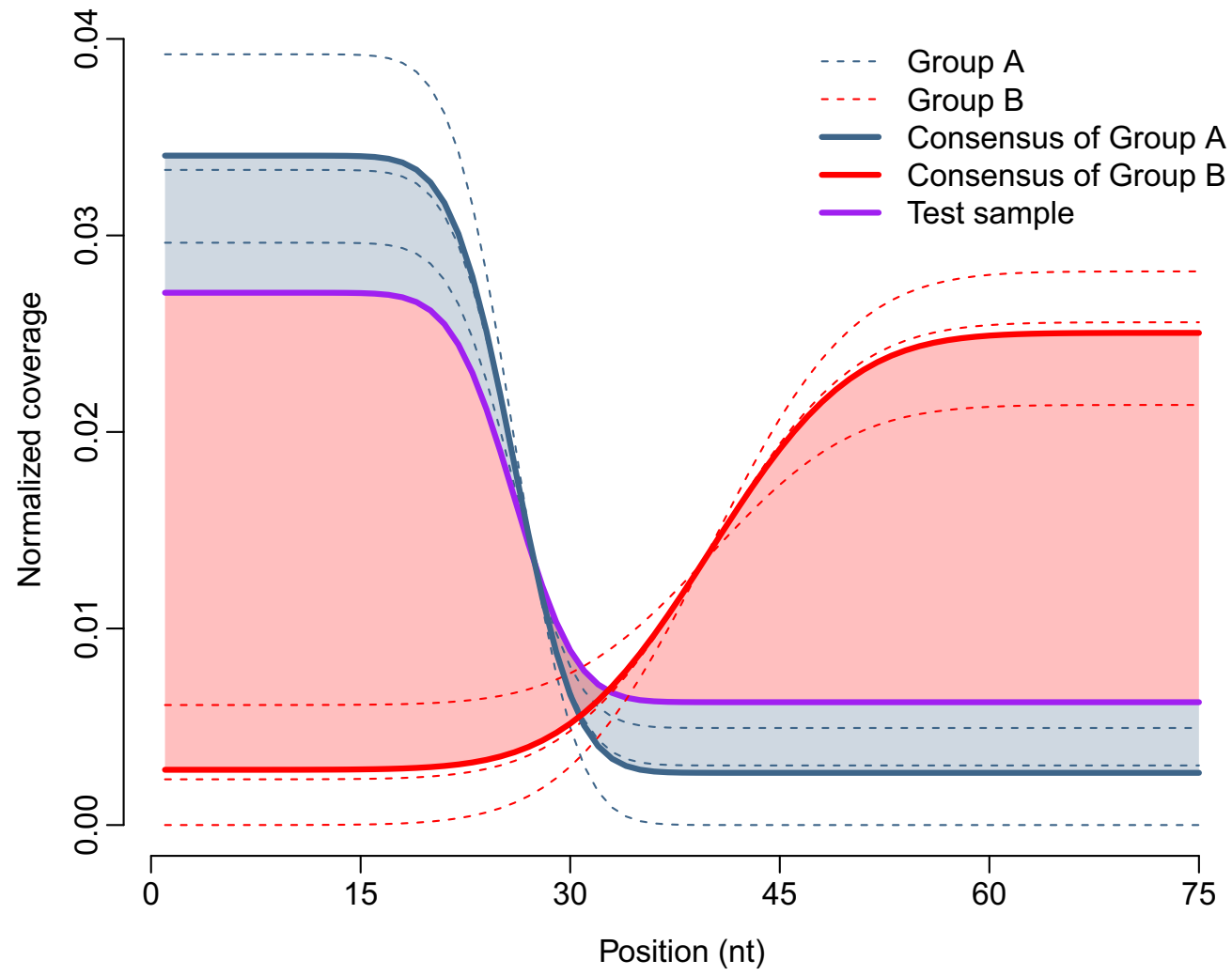

Figure EV4. Schematic diagram of consensus coverage.

We assume the fragmentation pattern of a given tRNA (with 75 nts in length) is analyzed for Group A ($n = 3$) and Group B ($n = 3$). The normalized coverage of tsRNAs is plotted along the parental tRNA for the individual samples within the two groups, illustrated as the blue/red dash curves. The solid blue and red curves represent the mean coverage pattern, i.e., "consensus coverage", for Group A and Group B, respectively. For a given test sample with unknown classification information, the normalized coverage of tsRNAs is plotted along the parental tRNA as well, illustrated as the purple solid curve. The light blue area surrounded by the solid blue and solid purple curves represents the deviation between the consensus coverage of Group A and the coverage pattern of the test sample. Meanwhile, the pink area surrounded by the solid red and solid purple curves represents the deviation between the consensus coverage of Group B and the coverage pattern of the test sample.

© The Author(s)

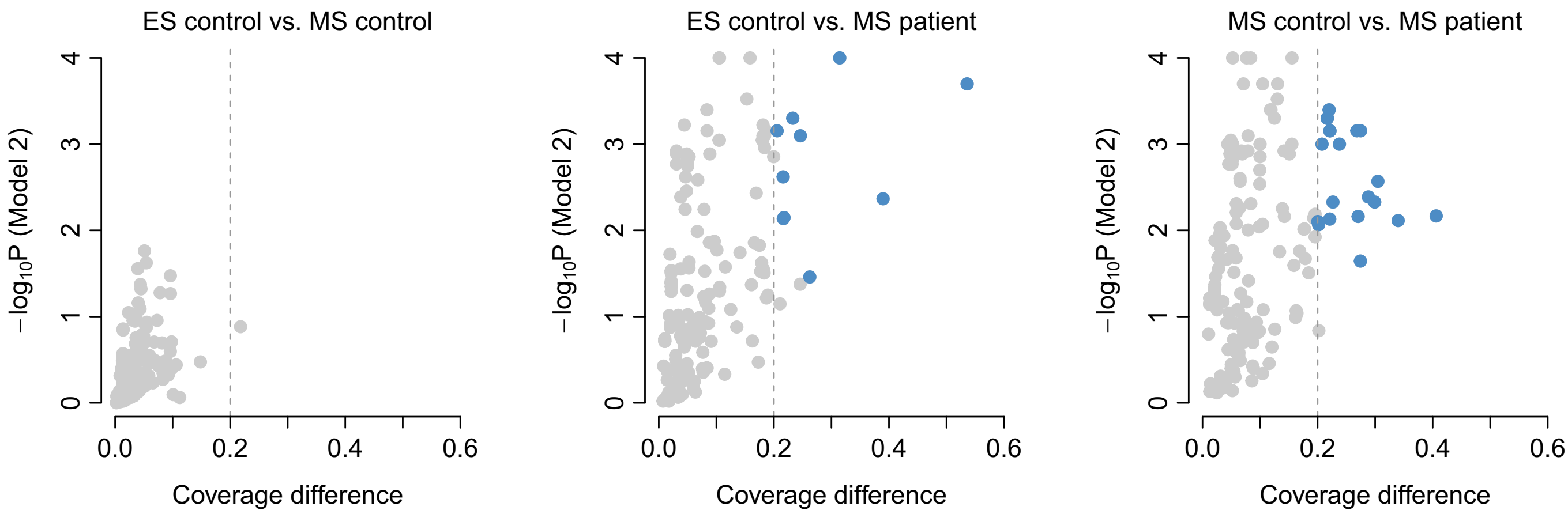

Figure EV5. Relationship between coverage difference and statistical significance in RIF. Each dot represents one parental tRNA/rRNA.

The blue dots denote the differentially fragmented parental RNAs. The *P* values illustrated by *Y* axis were calculated using *qMAP* Model 2. The data points with $P = 0$ were excluded from the scatter plots. The vertical line indicates the coverage difference level of 0.2.

 © The Author(s)

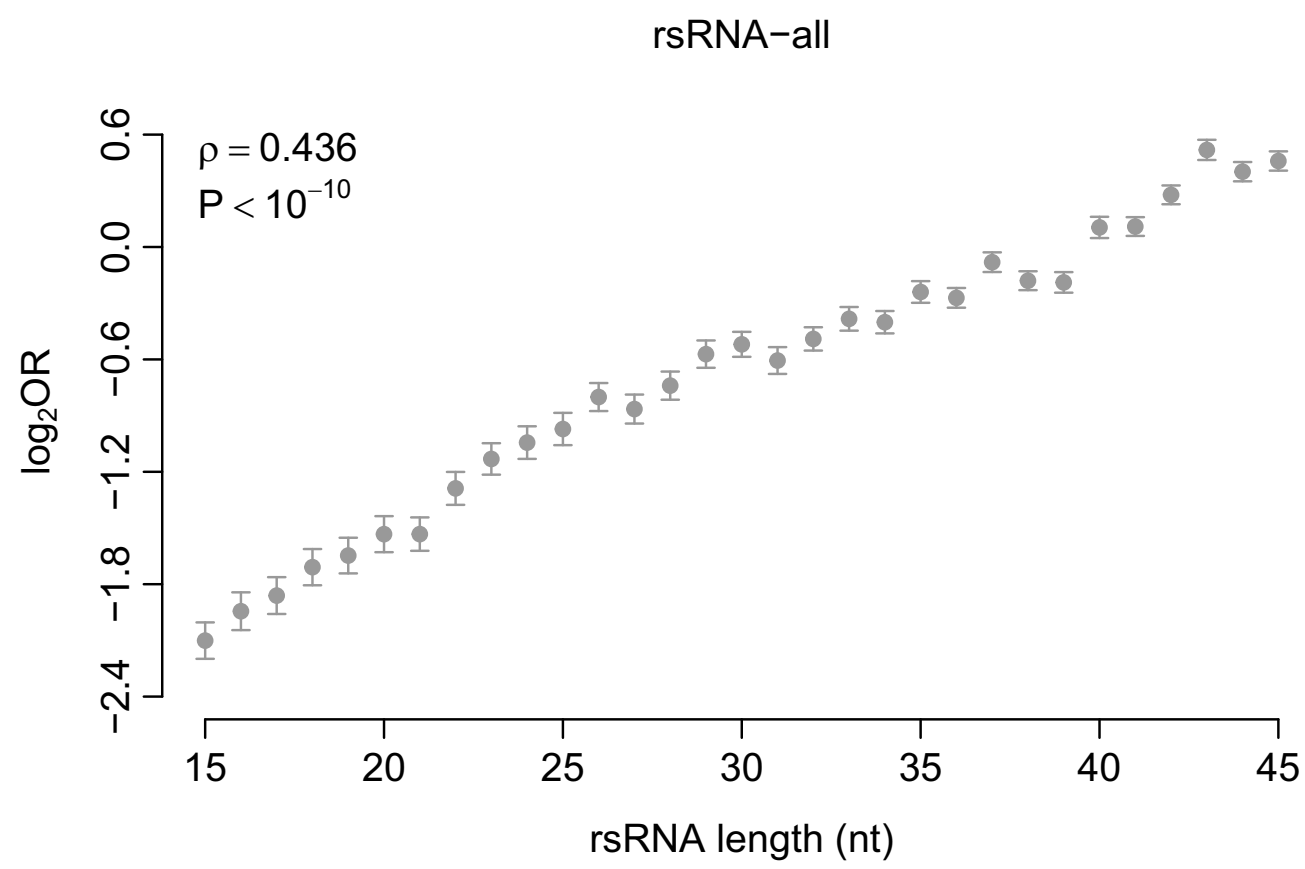

Figure EV6. Relationship between the length of rsRNAs in mouse sperm heads and $\log_2$-transformed odds ratio ($\log_2$*OR*).

The odds ratios were calculated using the *qMAP_mh* module, indicating the over-/under-representation status of the individual rsRNA species in old mice relative to young mice. The dots and error bars stand for the means and 95% confidence intervals of $\log_2 OR$, respectively ($n = 1070$–2603 per category). The correlation coefficient (ρ) and *P* value were computed using *Spearman*'s rank correlation test ($\rho = 0.436$ and $P = 0$).

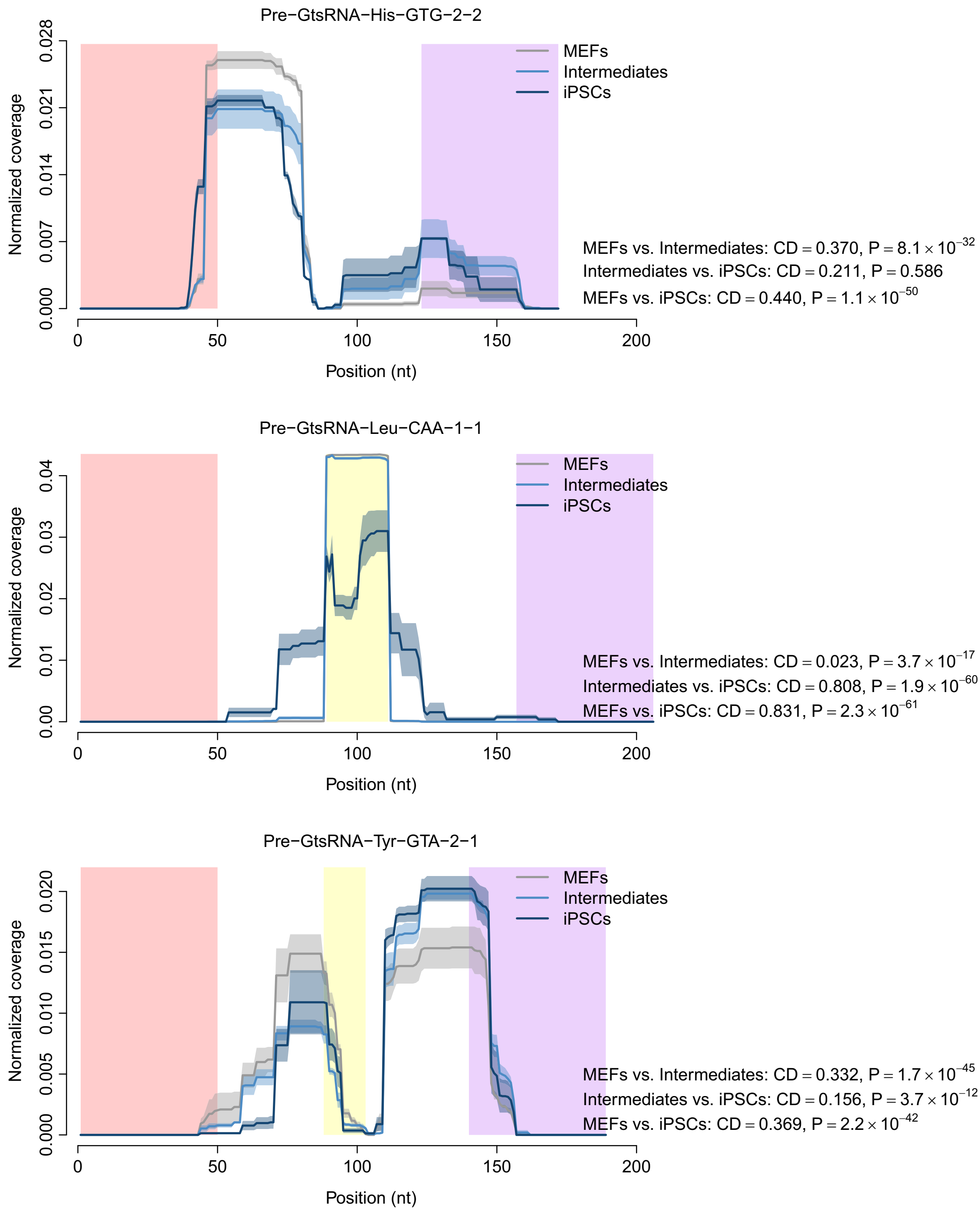

Figure EV7. Comparison in coverage pattern of pre-GtsRNA-His-GTG-2-2, pre-GtsRNA-Leu-CAA-1-1, and pre-GtsRNA-Tyr-GTA-2-1 between MEFs, intermediates, and iPSCs.

The solid curves indicate the mean normalized coverage along the pre-tRNAs, and the bands represent the SEM. The vertical pink, yellow, and purple bars indicate the 5′ leader, intron, and 3′ trailer of pre-tRNAs, respectively. The coverage difference (*CD*) and *P* value between the cell stages were calculated using *qMAP* Model 1.

© The Author(s)
