# Supplementary material for: qMAP decodes RNA fragmentation dynamics in development and disease
Source: Mol Syst Biol. 2026 Jul 20;22(10):1626–49. doi: 10.1038/s44320-026-00237-2 (PMC13638987; doi:10.1038/s44320-026-00237-2)
Supplement: Supplementary file 2 [file 44320_2026_237_MOESM1_ESM.pdf]

Table EV1. Differential fragmentation analysis between MEFs, intermediates, and iPSCs

			MEFs *vs.* intermediates			Intermediates *vs.* iPSCs			MEFs *vs.* iPSCs		
sncRNA family	sncRNA subfamily	Parental RNA	*CD*	Adjusted *P*		*CD*	Adjusted *P*		*CD*	Adjusted *P*	
GtsRNA-Ala-AGC	GtsRNA-Ala-AGC-1	Genomic tRNA-Ala-AGC-1	0.012	2.7E-04		0.083	4.6E-69		0.081	1.9E-82	
GtsRNA-Ala-AGC	GtsRNA-Ala-AGC-10	Genomic tRNA-Ala-AGC-10	0.429	1.4E-01		0.438	9.4E-06	*	0.338	2.8E-01	
GtsRNA-Ala-AGC	GtsRNA-Ala-AGC-2	Genomic tRNA-Ala-AGC-2	0.011	5.2E-02		0.108	1.6E-68		0.107	1.9E-83	
GtsRNA-Ala-AGC	GtsRNA-Ala-AGC-3	Genomic tRNA-Ala-AGC-3	0.041	5.8E-02		0.061	3.2E-59		0.033	3.6E-07	
GtsRNA-Ala-AGC	GtsRNA-Ala-AGC-4	Genomic tRNA-Ala-AGC-4	0.658	2.9E-20	*	0.332	2.2E-02	*	0.688	5.3E-15	*
GtsRNA-Ala-AGC	GtsRNA-Ala-AGC-5	Genomic tRNA-Ala-AGC-5	0.619	2.6E-07	*	0.399	1.1E-07	*	0.665	1.4E-10	*
GtsRNA-Ala-AGC	GtsRNA-Ala-AGC-6	Genomic tRNA-Ala-AGC-6	0.646	2.0E-18	*	0.419	2.1E-09	*	0.686	2.8E-17	*
GtsRNA-Ala-AGC	GtsRNA-Ala-AGC-7	Genomic tRNA-Ala-AGC-7	0.647	1.8E-11	*	0.474	4.9E-17	*	0.636	3.1E-07	*
GtsRNA-Ala-AGC	GtsRNA-Ala-AGC-8	Genomic tRNA-Ala-AGC-8	0.010	1.3E-01		0.032	6.1E-32		0.035	1.8E-24	
GtsRNA-Ala-CGC	GtsRNA-Ala-CGC-1	Genomic tRNA-Ala-CGC-1	0.045	2.8E-07		0.103	6.6E-61		0.065	6.8E-15	
GtsRNA-Ala-CGC	GtsRNA-Ala-CGC-2	Genomic tRNA-Ala-CGC-2	0.042	2.2E-07		0.082	6.5E-60		0.048	1.7E-13	
GtsRNA-Ala-CGC	GtsRNA-Ala-CGC-3	Genomic tRNA-Ala-CGC-3	0.004	1.0E+00		0.029	1.6E-26		0.030	1.8E-05	
GtsRNA-Ala-CGC	GtsRNA-Ala-CGC-4	Genomic tRNA-Ala-CGC-4	0.243	5.0E-09	*	0.485	4.1E-01		0.713	1.7E-05	*
GtsRNA-Ala-CGC	GtsRNA-Ala-CGC-5	Genomic tRNA-Ala-CGC-5	0.177	1.1E-04		0.101	1.3E-03		0.278	2.2E-13	*
GtsRNA-Ala-CGC	GtsRNA-Ala-CGC-6	Genomic tRNA-Ala-CGC-6	0.004	1.0E+00		0.028	8.1E-21		0.030	7.6E-06	
GtsRNA-Ala-CGC	GtsRNA-Ala-CGC-7	Genomic tRNA-Ala-CGC-7	0.045	9.5E-01		0.030	1.1E-25		0.049	1.7E-07	
GtsRNA-Ala-TGC	GtsRNA-Ala-TGC-1	Genomic tRNA-Ala-TGC-1	0.048	6.2E-15		0.122	3.5E-75		0.084	1.7E-19	
GtsRNA-Ala-TGC	GtsRNA-Ala-TGC-2	Genomic tRNA-Ala-TGC-2	0.046	1.3E-08		0.104	9.6E-70		0.065	3.4E-08	
GtsRNA-Ala-TGC	GtsRNA-Ala-TGC-4	Genomic tRNA-Ala-TGC-4	0.042	1.2E-07		0.112	1.3E-73		0.077	5.3E-14	
GtsRNA-Ala-TGC	GtsRNA-Ala-TGC-5	Genomic tRNA-Ala-TGC-5	0.046	1.5E-04		0.031	1.0E-19		0.043	2.6E-08	
GtsRNA-Ala-TGC	GtsRNA-Ala-TGC-6	Genomic tRNA-Ala-TGC-6	0.046	9.9E-02		0.046	3.4E-55		0.028	5.2E-16	
GtsRNA-Ala-TGC	GtsRNA-Ala-TGC-7	Genomic tRNA-Ala-TGC-7	0.042	1.9E-01		0.029	1.0E-29		0.044	4.0E-05	
GtsRNA-Ala-TGC	GtsRNA-Ala-TGC-8	Genomic tRNA-Ala-TGC-8	0.008	1.0E+00		0.028	4.6E-41		0.034	1.5E-32	
GtsRNA-Arg-ACG	GtsRNA-Arg-ACG-1	Genomic tRNA-Arg-ACG-1	0.208	1.6E-68	*	0.510	2.6E-80	*	0.718	4.2E-85	*
GtsRNA-Arg-ACG	GtsRNA-Arg-ACG-2	Genomic tRNA-Arg-ACG-2	0.339	9.6E-03	*	0.113	4.5E-02		0.445	2.1E-18	*
GtsRNA-Arg-ACG	GtsRNA-Arg-ACG-3	Genomic tRNA-Arg-ACG-3	0.240	3.4E-72	*	0.555	6.8E-70	*	0.795	1.0E-55	*
GtsRNA-Arg-CCG	GtsRNA-Arg-CCG-1	Genomic tRNA-Arg-CCG-1	0.430	3.5E-24	*	0.293	1.8E-28	*	0.715	7.4E-78	*
GtsRNA-Arg-CCG	GtsRNA-Arg-CCG-2	Genomic tRNA-Arg-CCG-2	0.465	3.7E-24	*	0.334	9.8E-39	*	0.787	1.1E-86	*
GtsRNA-Arg-CCG	GtsRNA-Arg-CCG-3	Genomic tRNA-Arg-CCG-3	0.210	3.4E-22	*	0.160	8.2E-01		0.102	1.3E-08	
GtsRNA-Arg-CCT	GtsRNA-Arg-CCT-1	Genomic tRNA-Arg-CCT-1	0.031	6.3E-01		0.112	1.2E-20		0.101	2.4E-13	
GtsRNA-Arg-CCT	GtsRNA-Arg-CCT-2	Genomic tRNA-Arg-CCT-2	0.129	1.3E-14		0.168	1.2E-26		0.272	1.2E-26	*
GtsRNA-Arg-CCT	GtsRNA-Arg-CCT-3	Genomic tRNA-Arg-CCT-3	0.033	4.3E-03		0.085	7.6E-15		0.071	4.0E-03	
GtsRNA-Arg-CCT	GtsRNA-Arg-CCT-4	Genomic tRNA-Arg-CCT-4	0.154	8.3E-18		0.153	4.4E-23		0.282	1.8E-26	*
GtsRNA-Arg-TCG	GtsRNA-Arg-TCG-1	Genomic tRNA-Arg-TCG-1	0.336	3.0E-42	*	0.082	1.5E-01		0.370	1.5E-43	*
GtsRNA-Arg-TCG	GtsRNA-Arg-TCG-2	Genomic tRNA-Arg-TCG-2	0.406	9.9E-46	*	0.085	2.8E-01		0.464	1.8E-53	*
GtsRNA-Arg-TCG	GtsRNA-Arg-TCG-3	Genomic tRNA-Arg-TCG-3	0.228	2.6E-19	*	0.066	1.0E+00		0.234	7.4E-21	*

			MEFs *vs.* intermediates			Intermediates *vs.* iPSCs			MEFs *vs.* iPSCs		
sncRNA family	sncRNA subfamily	Parental RNA	*CD*	Adjusted *P*		*CD*	Adjusted *P*		*CD*	Adjusted *P*	
GtsRNA-Arg-TCG	GtsRNA-Arg-TCG-4	Genomic tRNA-Arg-TCG-4	0.215	2.1E-18	*	0.067	1.0E+00		0.226	4.8E-17	*
GtsRNA-Arg-TCT	GtsRNA-Arg-TCT-1	Genomic tRNA-Arg-TCT-1	0.016	7.6E-01		0.050	3.6E-04		0.059	3.1E-10	
GtsRNA-Arg-TCT	GtsRNA-Arg-TCT-2	Genomic tRNA-Arg-TCT-2	0.015	9.9E-01		0.051	7.0E-07		0.060	2.4E-07	
GtsRNA-Arg-TCT	GtsRNA-Arg-TCT-3	Genomic tRNA-Arg-TCT-3	0.015	9.3E-01		0.052	1.3E-11		0.060	1.8E-16	
GtsRNA-Arg-TCT	GtsRNA-Arg-TCT-4	Genomic tRNA-Arg-TCT-4	0.469	1.7E-13	*	0.368	1.1E-07	*	0.620	4.2E-23	*
GtsRNA-Arg-TCT	GtsRNA-Arg-TCT-5	Genomic tRNA-Arg-TCT-5	0.020	6.3E-01		0.053	9.8E-17		0.060	5.7E-15	
GtsRNA-Asn-GTT	GtsRNA-Asn-GTT-1	Genomic tRNA-Asn-GTT-1	0.148	2.1E-02		0.114	2.3E-01		0.225	1.0E-07	*
GtsRNA-Asn-GTT	GtsRNA-Asn-GTT-2	Genomic tRNA-Asn-GTT-2	0.145	9.9E-02		0.119	2.6E-01		0.234	4.9E-09	*
GtsRNA-Asn-GTT	GtsRNA-Asn-GTT-3	Genomic tRNA-Asn-GTT-3	0.154	3.3E-02		0.120	2.6E-01		0.239	2.4E-10	*
GtsRNA-Asn-GTT	GtsRNA-Asn-GTT-4	Genomic tRNA-Asn-GTT-4	0.549	7.6E-03	*	0.444	1.2E-05	*	0.459	4.9E-02	*
GtsRNA-Asn-GTT	GtsRNA-Asn-GTT-5	Genomic tRNA-Asn-GTT-5	0.363	9.8E-13	*	0.347	3.4E-45	*	0.625	3.7E-49	*
GtsRNA-Asp-GTC	GtsRNA-Asp-GTC-1	Genomic tRNA-Asp-GTC-1	0.419	4.6E-64	*	0.619	3.5E-57	*	0.624	3.7E-40	*
GtsRNA-Asp-GTC	GtsRNA-Asp-GTC-2	Genomic tRNA-Asp-GTC-2	0.404	4.0E-63	*	0.639	8.1E-63	*	0.635	2.0E-40	*
GtsRNA-Asp-GTC	GtsRNA-Asp-GTC-3	Genomic tRNA-Asp-GTC-3	0.124	3.2E-16		0.364	1.5E-20	*	0.242	8.5E-09	*
GtsRNA-Asp-GTC	GtsRNA-Asp-GTC-4	Genomic tRNA-Asp-GTC-4	0.229	4.2E-10	*	0.500	6.5E-20	*	0.318	8.0E-05	*
GtsRNA-Cys-GCA	GtsRNA-Cys-GCA-1	Genomic tRNA-Cys-GCA-1	0.114	2.0E-04		0.637	1.1E-55	*	0.741	2.3E-53	*
GtsRNA-Cys-GCA	GtsRNA-Cys-GCA-10	Genomic tRNA-Cys-GCA-10	0.161	2.3E-10		0.306	3.9E-22	*	0.332	8.8E-28	*
GtsRNA-Cys-GCA	GtsRNA-Cys-GCA-11	Genomic tRNA-Cys-GCA-11	0.248	4.9E-01		0.749	1.1E-36	*	0.846	3.3E-57	*
GtsRNA-Cys-GCA	GtsRNA-Cys-GCA-12	Genomic tRNA-Cys-GCA-12	0.171	2.0E-24		0.294	2.8E-29	*	0.343	1.0E-45	*
GtsRNA-Cys-GCA	GtsRNA-Cys-GCA-13	Genomic tRNA-Cys-GCA-13	0.219	2.5E-33	*	0.311	1.7E-46	*	0.430	3.7E-55	*
GtsRNA-Cys-GCA	GtsRNA-Cys-GCA-14	Genomic tRNA-Cys-GCA-14	0.202	9.8E-22	*	0.326	3.2E-34	*	0.411	6.9E-63	*
GtsRNA-Cys-GCA	GtsRNA-Cys-GCA-15	Genomic tRNA-Cys-GCA-15	0.233	4.1E-13	*	0.277	9.9E-64	*	0.316	1.4E-31	*
GtsRNA-Cys-GCA	GtsRNA-Cys-GCA-16	Genomic tRNA-Cys-GCA-16	0.209	3.3E-02	*	0.388	3.2E-22	*	0.557	7.6E-59	*
GtsRNA-Cys-GCA	GtsRNA-Cys-GCA-17	Genomic tRNA-Cys-GCA-17	0.295	4.9E-28	*	0.305	2.5E-19	*	0.388	6.7E-37	*
GtsRNA-Cys-GCA	GtsRNA-Cys-GCA-18	Genomic tRNA-Cys-GCA-18	0.255	6.8E-05	*	0.707	4.8E-42	*	0.904	1.9E-60	*
GtsRNA-Cys-GCA	GtsRNA-Cys-GCA-19	Genomic tRNA-Cys-GCA-19	0.145	3.9E-01		0.421	2.2E-44	*	0.303	5.1E-39	*
GtsRNA-Cys-GCA	GtsRNA-Cys-GCA-2	Genomic tRNA-Cys-GCA-2	0.110	1.2E-02		0.636	1.5E-56	*	0.731	3.5E-57	*
GtsRNA-Cys-GCA	GtsRNA-Cys-GCA-21	Genomic tRNA-Cys-GCA-21	0.246	6.6E-08	*	0.484	5.4E-23	*	0.662	2.4E-41	*
GtsRNA-Cys-GCA	GtsRNA-Cys-GCA-24	Genomic tRNA-Cys-GCA-24	0.173	8.0E-17		0.272	4.4E-40	*	0.307	1.7E-44	*
GtsRNA-Cys-GCA	GtsRNA-Cys-GCA-25	Genomic tRNA-Cys-GCA-25	0.415	3.8E-22	*	0.775	2.9E-59	*	0.998	7.9E-57	*
GtsRNA-Cys-GCA	GtsRNA-Cys-GCA-3	Genomic tRNA-Cys-GCA-3	0.106	6.9E-02		0.629	8.4E-59	*	0.715	1.4E-60	*
GtsRNA-Cys-GCA	GtsRNA-Cys-GCA-4	Genomic tRNA-Cys-GCA-4	0.192	3.2E-10		0.328	4.4E-21	*	0.400	3.0E-50	*
GtsRNA-Cys-GCA	GtsRNA-Cys-GCA-5	Genomic tRNA-Cys-GCA-5	0.200	1.1E-13		0.329	3.4E-21	*	0.394	1.2E-49	*
GtsRNA-Cys-GCA	GtsRNA-Cys-GCA-6	Genomic tRNA-Cys-GCA-6	0.161	2.1E-10		0.306	3.2E-22	*	0.331	8.9E-29	*
GtsRNA-Cys-GCA	GtsRNA-Cys-GCA-7	Genomic tRNA-Cys-GCA-7	0.212	4.2E-21	*	0.327	3.9E-26	*	0.384	1.0E-53	*
GtsRNA-Cys-GCA	GtsRNA-Cys-GCA-8	Genomic tRNA-Cys-GCA-8	0.345	3.6E-46	*	0.299	7.5E-25	*	0.387	4.3E-39	*
GtsRNA-Cys-GCA	GtsRNA-Cys-GCA-9	Genomic tRNA-Cys-GCA-9	0.263	1.4E-18	*	0.420	1.1E-40	*	0.535	3.6E-66	*
GtsRNA-Gln-CTG	GtsRNA-Gln-CTG-1	Genomic tRNA-Gln-CTG-1	0.074	1.7E-04		0.102	2.1E-17		0.175	1.7E-72	

			MEFs *vs.* intermediates			Intermediates *vs.* iPSCs			MEFs *vs.* iPSCs		
sncRNA family	sncRNA subfamily	Parental RNA	*CD*	Adjusted *P*		*CD*	Adjusted *P*		*CD*	Adjusted *P*	
GtsRNA-Gln-CTG	GtsRNA-Gln-CTG-2	Genomic tRNA-Gln-CTG-2	0.074	2.7E-04		0.103	2.3E-16		0.176	1.1E-71	
GtsRNA-Gln-CTG	GtsRNA-Gln-CTG-3	Genomic tRNA-Gln-CTG-3	0.074	2.3E-04		0.102	6.0E-17		0.176	7.2E-72	
GtsRNA-Gln-CTG	GtsRNA-Gln-CTG-4	Genomic tRNA-Gln-CTG-4	0.075	3.8E-05		0.096	5.2E-18		0.170	1.4E-73	
GtsRNA-Gln-CTG	GtsRNA-Gln-CTG-5	Genomic tRNA-Gln-CTG-5	0.028	7.2E-01		0.070	5.6E-09		0.098	2.1E-62	
GtsRNA-Gln-TTG	GtsRNA-Gln-TTG-1	Genomic tRNA-Gln-TTG-1	0.078	5.8E-07		0.102	3.8E-17		0.179	2.6E-73	
GtsRNA-Gln-TTG	GtsRNA-Gln-TTG-2	Genomic tRNA-Gln-TTG-2	0.076	1.3E-04		0.098	1.4E-12		0.174	2.4E-63	
GtsRNA-Gln-TTG	GtsRNA-Gln-TTG-3	Genomic tRNA-Gln-TTG-3	0.078	2.4E-09		0.102	5.9E-17		0.179	6.3E-71	
GtsRNA-Gln-TTG	GtsRNA-Gln-TTG-4	Genomic tRNA-Gln-TTG-4	0.086	1.0E+00		0.195	2.5E-07		0.184	1.8E-11	
GtsRNA-Gln-TTG	GtsRNA-Gln-TTG-5	Genomic tRNA-Gln-TTG-5	0.076	2.1E-10		0.098	1.1E-12		0.174	1.5E-66	
GtsRNA-Glu-CTC	GtsRNA-Glu-CTC-1	Genomic tRNA-Glu-CTC-1	0.177	6.2E-09		0.070	1.0E+00		0.143	3.4E-19	
GtsRNA-Glu-CTC	GtsRNA-Glu-CTC-2	Genomic tRNA-Glu-CTC-2	0.179	6.3E-09		0.069	1.0E+00		0.147	4.1E-20	
GtsRNA-Glu-CTC	GtsRNA-Glu-CTC-5	Genomic tRNA-Glu-CTC-5	0.018	9.3E-01		0.055	1.0E-05		0.065	1.8E-04	
GtsRNA-Glu-TTC	GtsRNA-Glu-TTC-1	Genomic tRNA-Glu-TTC-1	0.328	5.4E-17	*	0.157	1.4E-01		0.462	2.5E-60	*
GtsRNA-Glu-TTC	GtsRNA-Glu-TTC-2	Genomic tRNA-Glu-TTC-2	0.328	6.9E-16	*	0.159	8.2E-02		0.461	1.5E-58	*
GtsRNA-Glu-TTC	GtsRNA-Glu-TTC-3	Genomic tRNA-Glu-TTC-3	0.145	1.5E-11		0.169	1.6E-12		0.128	3.2E-28	
GtsRNA-Gly-ACC	GtsRNA-Gly-ACC-1	Genomic tRNA-Gly-ACC-1	0.194	5.0E-02		0.028	2.3E-01		0.176	1.7E-13	
GtsRNA-Gly-CCC	GtsRNA-Gly-CCC-1	Genomic tRNA-Gly-CCC-1	0.453	3.5E-29	*	0.382	4.2E-36	*	0.793	2.0E-45	*
GtsRNA-Gly-CCC	GtsRNA-Gly-CCC-2	Genomic tRNA-Gly-CCC-2	0.392	6.8E-13	*	0.486	1.2E-38	*	0.841	3.1E-67	*
GtsRNA-Gly-CCC	GtsRNA-Gly-CCC-3	Genomic tRNA-Gly-CCC-3	0.097	1.0E+00		0.170	6.2E-23		0.250	1.0E-18	*
GtsRNA-Gly-CCC	GtsRNA-Gly-CCC-4	Genomic tRNA-Gly-CCC-4	0.106	1.0E+00		0.161	2.4E-21		0.251	3.7E-14	*
GtsRNA-Gly-GCC	GtsRNA-Gly-GCC-1	Genomic tRNA-Gly-GCC-1	0.110	8.4E-01		0.153	1.0E-23		0.247	3.2E-27	*
GtsRNA-Gly-GCC	GtsRNA-Gly-GCC-2	Genomic tRNA-Gly-GCC-2	0.116	4.3E-01		0.142	4.2E-24		0.237	9.7E-27	*
GtsRNA-Gly-GCC	GtsRNA-Gly-GCC-3	Genomic tRNA-Gly-GCC-3	0.103	1.3E-01		0.128	1.6E-18		0.217	4.3E-30	*
GtsRNA-Gly-GCC	GtsRNA-Gly-GCC-4	Genomic tRNA-Gly-GCC-4	0.102	1.4E-01		0.128	1.6E-20		0.216	3.1E-30	*
GtsRNA-Gly-TCC	GtsRNA-Gly-TCC-1	Genomic tRNA-Gly-TCC-1	0.141	9.1E-01		0.747	7.4E-63	*	0.883	1.1E-95	*
GtsRNA-His-GTG	GtsRNA-His-GTG-1	Genomic tRNA-His-GTG-1	0.105	5.6E-06		0.069	3.5E-01		0.156	3.1E-35	
GtsRNA-His-GTG	GtsRNA-His-GTG-2	Genomic tRNA-His-GTG-2	0.027	1.0E+00		0.240	5.1E-39	*	0.239	2.0E-42	*
GtsRNA-His-GTG	GtsRNA-His-GTG-3	Genomic tRNA-His-GTG-3	0.028	1.0E+00		0.260	1.5E-35	*	0.256	5.1E-41	*
GtsRNA-Ile-AAT	GtsRNA-Ile-AAT-1	Genomic tRNA-Ile-AAT-1	0.286	1.0E+00		0.415	4.2E-07	*	0.581	2.0E-32	*
GtsRNA-Ile-AAT	GtsRNA-Ile-AAT-2	Genomic tRNA-Ile-AAT-2	0.421	6.5E-01		0.582	2.7E-10	*	0.902	2.6E-34	*
GtsRNA-Ile-AAT	GtsRNA-Ile-AAT-3	Genomic tRNA-Ile-AAT-3	0.314	8.1E-01		0.515	1.4E-18	*	0.711	8.4E-36	*
GtsRNA-Ile-AAT	GtsRNA-Ile-AAT-4	Genomic tRNA-Ile-AAT-4	0.460	8.3E-04	*	0.818	6.0E-16	*	0.693	7.0E-33	*
GtsRNA-Ile-TAT	GtsRNA-Ile-TAT-1	Genomic tRNA-Ile-TAT-1	0.579	4.7E-41	*	0.204	3.9E-26	*	0.756	2.0E-51	*
GtsRNA-Ile-TAT	GtsRNA-Ile-TAT-2	Genomic tRNA-Ile-TAT-2	0.435	3.1E-23	*	0.195	7.0E-28		0.600	3.2E-35	*
GtsRNA-Ile-TAT	GtsRNA-Ile-TAT-3	Genomic tRNA-Ile-TAT-3	0.568	2.8E-12	*	0.343	4.8E-06	*	0.368	1.3E-07	*
GtsRNA-iMet-CAT	GtsRNA-iMet-CAT-1	Genomic tRNA-iMet-CAT-1	0.171	3.2E-39		0.165	1.0E-07		0.197	7.1E-20	
GtsRNA-iMet-CAT	GtsRNA-iMet-CAT-2	Genomic tRNA-iMet-CAT-2	0.186	6.6E-41		0.174	5.5E-08		0.207	1.4E-19	*
GtsRNA-iMet-CAT	GtsRNA-iMet-CAT-3	Genomic tRNA-iMet-CAT-3	0.175	7.3E-41		0.154	7.9E-08		0.192	1.8E-20	

			MEFs *vs.* intermediates			Intermediates *vs.* iPSCs			MEFs *vs.* iPSCs		
sncRNA family	sncRNA subfamily	Parental RNA	*CD*	Adjusted *P*		*CD*	Adjusted *P*		*CD*	Adjusted *P*	
GtsRNA-Leu-AAG	GtsRNA-Leu-AAG-1	Genomic tRNA-Leu-AAG-1	0.613	3.5E-11	*	0.770	5.3E-19	*	1.073	5.6E-63	*
GtsRNA-Leu-AAG	GtsRNA-Leu-AAG-2	Genomic tRNA-Leu-AAG-2	0.610	2.4E-11	*	0.755	1.6E-18	*	1.074	8.7E-65	*
GtsRNA-Leu-AAG	GtsRNA-Leu-AAG-3	Genomic tRNA-Leu-AAG-3	0.616	9.9E-12	*	0.752	7.7E-18	*	1.077	1.8E-64	*
GtsRNA-Leu-CAA	GtsRNA-Leu-CAA-1	Genomic tRNA-Leu-CAA-1	0.257	7.6E-74	*	0.369	1.1E-40	*	0.367	3.5E-57	*
GtsRNA-Leu-CAA	GtsRNA-Leu-CAA-2	Genomic tRNA-Leu-CAA-2	0.295	6.4E-68	*	0.380	1.7E-42	*	0.401	3.9E-70	*
GtsRNA-Leu-CAA	GtsRNA-Leu-CAA-3	Genomic tRNA-Leu-CAA-3	0.298	6.4E-68	*	0.378	1.1E-44	*	0.404	3.3E-72	*
GtsRNA-Leu-CAA	GtsRNA-Leu-CAA-4	Genomic tRNA-Leu-CAA-4	0.300	1.7E-74	*	0.370	3.5E-42	*	0.402	5.0E-71	*
GtsRNA-Leu-CAG	GtsRNA-Leu-CAG-1	Genomic tRNA-Leu-CAG-1	0.236	2.4E-58	*	0.378	6.6E-49	*	0.356	2.0E-73	*
GtsRNA-Leu-CAG	GtsRNA-Leu-CAG-2	Genomic tRNA-Leu-CAG-2	0.241	3.3E-57	*	0.372	5.2E-42	*	0.343	4.1E-76	*
GtsRNA-Leu-CAG	GtsRNA-Leu-CAG-3	Genomic tRNA-Leu-CAG-3	0.229	4.4E-57	*	0.353	2.7E-41	*	0.290	1.2E-54	*
GtsRNA-Leu-CAG	GtsRNA-Leu-CAG-4	Genomic tRNA-Leu-CAG-4	0.260	3.0E-65	*	0.375	2.8E-57	*	0.395	2.0E-73	*
GtsRNA-Leu-TAA	GtsRNA-Leu-TAA-1	Genomic tRNA-Leu-TAA-1	0.414	6.0E-29	*	0.202	3.9E-05	*	0.578	4.6E-61	*
GtsRNA-Leu-TAA	GtsRNA-Leu-TAA-2	Genomic tRNA-Leu-TAA-2	0.314	3.1E-03	*	0.522	8.3E-11	*	0.811	1.5E-54	*
GtsRNA-Leu-TAA	GtsRNA-Leu-TAA-3	Genomic tRNA-Leu-TAA-3	0.366	6.5E-56	*	0.096	3.4E-03		0.446	1.6E-63	*
GtsRNA-Leu-TAA	GtsRNA-Leu-TAA-4	Genomic tRNA-Leu-TAA-4	0.137	9.9E-10		0.050	6.1E-04		0.168	2.3E-22	
GtsRNA-Leu-TAG	GtsRNA-Leu-TAG-1	Genomic tRNA-Leu-TAG-1	0.683	8.9E-17	*	0.796	9.5E-22	*	1.151	2.3E-55	*
GtsRNA-Leu-TAG	GtsRNA-Leu-TAG-2	Genomic tRNA-Leu-TAG-2	0.688	1.9E-20	*	0.791	6.2E-23	*	1.173	3.2E-59	*
GtsRNA-Leu-TAG	GtsRNA-Leu-TAG-3	Genomic tRNA-Leu-TAG-3	0.420	2.0E-04	*	0.840	1.1E-40	*	1.023	1.3E-79	*
GtsRNA-Lys-CTT	GtsRNA-Lys-CTT-1	Genomic tRNA-Lys-CTT-1	0.231	8.2E-13	*	0.421	1.1E-36	*	0.596	1.8E-38	*
GtsRNA-Lys-CTT	GtsRNA-Lys-CTT-12	Genomic tRNA-Lys-CTT-12	0.546	9.2E-40	*	0.335	2.9E-20	*	0.878	6.7E-51	*
GtsRNA-Lys-CTT	GtsRNA-Lys-CTT-13	Genomic tRNA-Lys-CTT-13	0.038	9.3E-01		0.084	7.0E-10		0.089	3.9E-04	
GtsRNA-Lys-CTT	GtsRNA-Lys-CTT-14	Genomic tRNA-Lys-CTT-14	0.138	1.3E-10		0.237	1.9E-15	*	0.337	1.8E-50	*
GtsRNA-Lys-CTT	GtsRNA-Lys-CTT-2	Genomic tRNA-Lys-CTT-2	0.216	4.2E-07	*	0.242	6.3E-16	*	0.304	7.3E-24	*
GtsRNA-Lys-CTT	GtsRNA-Lys-CTT-3	Genomic tRNA-Lys-CTT-3	0.241	1.5E-14	*	0.194	1.8E-07		0.243	4.8E-08	*
GtsRNA-Lys-CTT	GtsRNA-Lys-CTT-4	Genomic tRNA-Lys-CTT-4	0.395	5.1E-25	*	0.217	5.3E-03	*	0.596	1.9E-60	*
GtsRNA-Lys-CTT	GtsRNA-Lys-CTT-5	Genomic tRNA-Lys-CTT-5	0.171	1.8E-04		0.078	8.2E-01		0.243	2.8E-32	*
GtsRNA-Lys-CTT	GtsRNA-Lys-CTT-6	Genomic tRNA-Lys-CTT-6	0.144	3.1E-01		0.105	5.5E-04		0.154	2.0E-04	
GtsRNA-Lys-CTT	GtsRNA-Lys-CTT-7	Genomic tRNA-Lys-CTT-7	0.483	6.1E-51	*	0.191	7.0E-17		0.639	8.5E-64	*
GtsRNA-Lys-CTT	GtsRNA-Lys-CTT-8	Genomic tRNA-Lys-CTT-8	0.089	6.8E-07		0.090	8.3E-12		0.075	1.9E-08	
GtsRNA-Lys-CTT	GtsRNA-Lys-CTT-9	Genomic tRNA-Lys-CTT-9	0.082	1.0E+00		0.049	1.0E+00		0.052	1.0E+00	
GtsRNA-Lys-TTT	GtsRNA-Lys-TTT-1	Genomic tRNA-Lys-TTT-1	0.260	8.2E-48	*	0.117	2.3E-10		0.308	3.4E-66	*
GtsRNA-Lys-TTT	GtsRNA-Lys-TTT-2	Genomic tRNA-Lys-TTT-2	0.231	2.8E-51	*	0.155	1.3E-22		0.374	2.0E-82	*
GtsRNA-Lys-TTT	GtsRNA-Lys-TTT-3	Genomic tRNA-Lys-TTT-3	0.045	6.6E-37		0.085	7.1E-38		0.115	6.8E-58	
GtsRNA-Lys-TTT	GtsRNA-Lys-TTT-4	Genomic tRNA-Lys-TTT-4	0.393	1.6E-24	*	0.217	8.0E-05	*	0.227	1.4E-10	*
GtsRNA-Lys-TTT	GtsRNA-Lys-TTT-5	Genomic tRNA-Lys-TTT-5	0.810	2.3E-50	*	0.421	1.0E-33	*	0.533	4.5E-34	*
GtsRNA-Met-CAT	GtsRNA-Met-CAT-1	Genomic tRNA-Met-CAT-1	0.427	9.9E-06	*	0.217	4.8E-41	*	0.637	1.2E-53	*
GtsRNA-Met-CAT	GtsRNA-Met-CAT-2	Genomic tRNA-Met-CAT-2	0.160	3.2E-09		0.144	2.3E-20		0.262	1.8E-15	*
GtsRNA-Met-CAT	GtsRNA-Met-CAT-3	Genomic tRNA-Met-CAT-3	0.162	2.5E-11		0.143	1.1E-19		0.262	4.9E-14	*

sncRNA family	sncRNA subfamily	Parental RNA	MEFs *vs.* intermediates			Intermediates *vs.* iPSCs			MEFs *vs.* iPSCs		
			CD	Adjusted *P*		*CD*	Adjusted *P*		*CD*	Adjusted *P*	
GtsRNA-Met-CAT	GtsRNA-Met-CAT-4	Genomic tRNA-Met-CAT-4	0.168	1.2E-14		0.148	5.2E-21		0.274	9.7E-17	*
GtsRNA-Met-CAT	GtsRNA-Met-CAT-5	Genomic tRNA-Met-CAT-5	0.166	2.2E-10		0.141	5.5E-16		0.268	7.0E-15	*
GtsRNA-Met-CAT	GtsRNA-Met-CAT-6	Genomic tRNA-Met-CAT-6	0.162	1.1E-14		0.136	7.3E-26		0.260	1.3E-17	*
GtsRNA-Phe-GAA	GtsRNA-Phe-GAA-1	Genomic tRNA-Phe-GAA-1	0.289	3.7E-24	*	0.513	6.9E-26	*	0.608	8.1E-28	*
GtsRNA-Phe-GAA	GtsRNA-Phe-GAA-2	Genomic tRNA-Phe-GAA-2	0.325	7.0E-33	*	0.534	1.9E-29	*	0.644	2.0E-28	*
GtsRNA-Phe-GAA	GtsRNA-Phe-GAA-3	Genomic tRNA-Phe-GAA-3	0.126	5.2E-01		0.450	8.5E-13	*	0.412	5.6E-24	*
GtsRNA-Pro-AGG	GtsRNA-Pro-AGG-1	Genomic tRNA-Pro-AGG-1	0.096	8.2E-01		0.132	3.3E-01		0.111	8.1E-22	
GtsRNA-Pro-CGG	GtsRNA-Pro-CGG-1	Genomic tRNA-Pro-CGG-1	0.096	8.2E-01		0.132	3.6E-01		0.111	5.1E-22	
GtsRNA-Pro-TGG	GtsRNA-Pro-TGG-1	Genomic tRNA-Pro-TGG-1	0.105	9.3E-01		0.142	9.7E-02		0.112	7.8E-22	
GtsRNA-Pro-TGG	GtsRNA-Pro-TGG-2	Genomic tRNA-Pro-TGG-2	0.096	8.1E-01		0.132	3.4E-01		0.112	3.2E-21	
GtsRNA-Pro-TGG	GtsRNA-Pro-TGG-3	Genomic tRNA-Pro-TGG-3	0.107	5.5E-33		0.223	1.9E-28	*	0.187	9.0E-40	
GtsRNA-Pro-TGG	GtsRNA-Pro-TGG-4	Genomic tRNA-Pro-TGG-4	0.035	2.2E-01		0.084	7.3E-01		0.111	2.4E-20	
GtsRNA-SeC-TCA	GtsRNA-SeC-TCA-1	Genomic tRNA-SeC-TCA-1	0.619	2.9E-11	*	0.406	2.2E-15	*	0.642	9.0E-26	*
GtsRNA-Ser-AGA	GtsRNA-Ser-AGA-1	Genomic tRNA-Ser-AGA-1	0.549	3.8E-24	*	0.899	9.8E-73	*	1.439	6.0E-73	*
GtsRNA-Ser-AGA	GtsRNA-Ser-AGA-2	Genomic tRNA-Ser-AGA-2	0.549	4.1E-24	*	0.898	2.9E-71	*	1.437	9.3E-74	*
GtsRNA-Ser-AGA	GtsRNA-Ser-AGA-3	Genomic tRNA-Ser-AGA-3	0.911	8.9E-54	*	0.748	8.2E-52	*	1.658	4.0E-53	*
GtsRNA-Ser-CGA	GtsRNA-Ser-CGA-1	Genomic tRNA-Ser-CGA-1	0.018	1.2E-03		0.030	3.6E-22		0.045	3.5E-65	
GtsRNA-Ser-CGA	GtsRNA-Ser-CGA-2	Genomic tRNA-Ser-CGA-2	0.039	1.4E-20		0.046	4.5E-15		0.080	3.2E-54	
GtsRNA-Ser-CGA	GtsRNA-Ser-CGA-3	Genomic tRNA-Ser-CGA-3	0.012	7.3E-01		0.025	3.3E-21		0.031	1.8E-35	
GtsRNA-Ser-GCT	GtsRNA-Ser-GCT-1	Genomic tRNA-Ser-GCT-1	0.584	1.4E-67	*	0.355	7.2E-69	*	0.940	6.9E-119	*
GtsRNA-Ser-GCT	GtsRNA-Ser-GCT-2	Genomic tRNA-Ser-GCT-2	0.711	1.0E-43	*	0.590	9.9E-64	*	1.299	2.1E-111	*
GtsRNA-Ser-GCT	GtsRNA-Ser-GCT-3	Genomic tRNA-Ser-GCT-3	0.705	1.5E-44	*	0.594	9.9E-68	*	1.299	1.8E-108	*
GtsRNA-Ser-GCT	GtsRNA-Ser-GCT-4	Genomic tRNA-Ser-GCT-4	0.697	2.4E-35	*	0.613	2.5E-65	*	1.305	2.9E-109	*
GtsRNA-Ser-GCT	GtsRNA-Ser-GCT-5	Genomic tRNA-Ser-GCT-5	0.759	1.1E-89	*	0.377	1.8E-76	*	1.137	1.0E-121	*
GtsRNA-Ser-GGA	GtsRNA-Ser-GGA-1	Genomic tRNA-Ser-GGA-1	0.155	2.7E-13		0.451	5.6E-40	*	0.565	1.2E-38	*
GtsRNA-Ser-TGA	GtsRNA-Ser-TGA-1	Genomic tRNA-Ser-TGA-1	0.142	1.6E-34		0.063	1.1E-43		0.198	1.1E-42	
GtsRNA-Ser-TGA	GtsRNA-Ser-TGA-2	Genomic tRNA-Ser-TGA-2	0.537	3.5E-29	*	0.668	1.7E-68	*	1.205	5.5E-79	*
GtsRNA-Sup-TCA	GtsRNA-Sup-TCA-1	Genomic tRNA-Sup-TCA-1	0.666	1.2E-37	*	0.167	3.9E-11		0.756	1.0E-52	*
GtsRNA-Sup-TTA	GtsRNA-Sup-TTA-1	Genomic tRNA-Sup-TTA-1	0.354	5.1E-05	*	0.383	5.4E-10	*	0.234	3.8E-03	*
GtsRNA-Thr-AGT	GtsRNA-Thr-AGT-1	Genomic tRNA-Thr-AGT-1	0.502	5.4E-26	*	0.457	6.8E-08	*	0.954	1.8E-57	*
GtsRNA-Thr-AGT	GtsRNA-Thr-AGT-2	Genomic tRNA-Thr-AGT-2	0.498	1.2E-29	*	0.451	1.6E-09	*	0.943	5.6E-53	*
GtsRNA-Thr-AGT	GtsRNA-Thr-AGT-3	Genomic tRNA-Thr-AGT-3	0.497	1.9E-29	*	0.462	2.2E-11	*	0.953	1.7E-57	*
GtsRNA-Thr-AGT	GtsRNA-Thr-AGT-4	Genomic tRNA-Thr-AGT-4	0.564	2.0E-18	*	0.305	3.2E-12	*	0.859	1.0E-46	*
GtsRNA-Thr-AGT	GtsRNA-Thr-AGT-5	Genomic tRNA-Thr-AGT-5	0.068	9.2E-01		0.045	1.0E+00		0.065	1.1E-17	
GtsRNA-Thr-AGT	GtsRNA-Thr-AGT-6	Genomic tRNA-Thr-AGT-6	0.153	9.9E-02		0.046	6.1E-01		0.143	2.4E-09	
GtsRNA-Thr-AGT	GtsRNA-Thr-AGT-7	Genomic tRNA-Thr-AGT-7	0.517	5.5E-27	*	0.462	1.2E-10	*	0.975	5.2E-64	*
GtsRNA-Thr-CGT	GtsRNA-Thr-CGT-1	Genomic tRNA-Thr-CGT-1	0.455	7.8E-10	*	0.425	7.5E-03	*	0.873	5.7E-67	*
GtsRNA-Thr-CGT	GtsRNA-Thr-CGT-2	Genomic tRNA-Thr-CGT-2	0.455	6.7E-09	*	0.409	1.9E-02	*	0.852	7.9E-64	*

			MEFs *vs.* intermediates			Intermediates *vs.* iPSCs			MEFs *vs.* iPSCs		
sncRNA family	sncRNA subfamily	Parental RNA	*CD*	Adjusted *P*		*CD*	Adjusted *P*		*CD*	Adjusted *P*	
GtsRNA-Thr-CGT	GtsRNA-Thr-CGT-3	Genomic tRNA-Thr-CGT-3	0.119	5.8E-01		0.045	1.0E+00		0.114	3.8E-12	
GtsRNA-Thr-CGT	GtsRNA-Thr-CGT-4	Genomic tRNA-Thr-CGT-4	0.128	1.7E-02		0.042	1.0E+00		0.121	1.1E-09	
GtsRNA-Thr-TGT	GtsRNA-Thr-TGT-1	Genomic tRNA-Thr-TGT-1	0.130	2.5E-02		0.055	1.1E-01		0.123	1.4E-07	
GtsRNA-Thr-TGT	GtsRNA-Thr-TGT-2	Genomic tRNA-Thr-TGT-2	0.456	8.5E-38	*	0.686	3.0E-67	*	1.006	1.0E-55	*
GtsRNA-Thr-TGT	GtsRNA-Thr-TGT-3	Genomic tRNA-Thr-TGT-3	0.454	1.2E-37	*	0.685	5.0E-61	*	1.004	7.1E-56	*
GtsRNA-Trp-CCA	GtsRNA-Trp-CCA-1	Genomic tRNA-Trp-CCA-1	0.055	1.0E+00		0.202	2.1E-23	*	0.238	2.1E-54	*
GtsRNA-Trp-CCA	GtsRNA-Trp-CCA-2	Genomic tRNA-Trp-CCA-2	0.050	1.0E+00		0.178	2.2E-07		0.199	5.9E-12	
GtsRNA-Trp-CCA	GtsRNA-Trp-CCA-3	Genomic tRNA-Trp-CCA-3	0.055	1.0E+00		0.204	1.2E-32	*	0.238	3.9E-55	*
GtsRNA-Trp-CCA	GtsRNA-Trp-CCA-4	Genomic tRNA-Trp-CCA-4	0.055	1.0E+00		0.204	7.7E-33	*	0.238	7.2E-57	*
GtsRNA-Trp-CCA	GtsRNA-Trp-CCA-5	Genomic tRNA-Trp-CCA-5	0.056	1.0E+00		0.211	5.4E-23	*	0.247	2.6E-56	*
GtsRNA-Trp-CCA	GtsRNA-Trp-CCA-6	Genomic tRNA-Trp-CCA-6	0.055	1.3E-01		0.181	2.1E-27		0.219	5.7E-56	*
GtsRNA-Tyr-GTA	GtsRNA-Tyr-GTA-1	Genomic tRNA-Tyr-GTA-1	0.194	2.2E-21		0.232	6.5E-18	*	0.180	5.9E-21	
GtsRNA-Tyr-GTA	GtsRNA-Tyr-GTA-2	Genomic tRNA-Tyr-GTA-2	0.192	5.1E-23		0.224	1.5E-17	*	0.173	5.2E-20	
GtsRNA-Tyr-GTA	GtsRNA-Tyr-GTA-3	Genomic tRNA-Tyr-GTA-3	0.194	2.6E-23		0.224	9.8E-20	*	0.174	3.1E-19	
GtsRNA-Tyr-GTA	GtsRNA-Tyr-GTA-4	Genomic tRNA-Tyr-GTA-4	0.198	1.5E-22		0.236	4.9E-20	*	0.184	5.2E-19	
GtsRNA-Tyr-GTA	GtsRNA-Tyr-GTA-5	Genomic tRNA-Tyr-GTA-5	0.163	7.1E-25		0.207	3.3E-11	*	0.190	9.8E-37	
GtsRNA-Val-AAC	GtsRNA-Val-AAC-1	Genomic tRNA-Val-AAC-1	0.114	6.0E-01		0.028	4.2E-05		0.103	4.1E-20	
GtsRNA-Val-AAC	GtsRNA-Val-AAC-2	Genomic tRNA-Val-AAC-2	0.135	2.6E-02		0.019	9.2E-03		0.122	1.4E-06	
GtsRNA-Val-AAC	GtsRNA-Val-AAC-3	Genomic tRNA-Val-AAC-3	0.138	1.9E-02		0.017	1.5E-02		0.126	6.3E-06	
GtsRNA-Val-AAC	GtsRNA-Val-AAC-4	Genomic tRNA-Val-AAC-4	0.074	1.1E-02		0.036	4.2E-05		0.053	9.9E-04	
GtsRNA-Val-AAC	GtsRNA-Val-AAC-5	Genomic tRNA-Val-AAC-5	0.113	6.6E-01		0.017	1.5E-03		0.104	6.2E-19	
GtsRNA-Val-AAC	GtsRNA-Val-AAC-6	Genomic tRNA-Val-AAC-6	0.026	4.7E-03		0.129	7.8E-06		0.115	3.2E-03	
GtsRNA-Val-CAC	GtsRNA-Val-CAC-1	Genomic tRNA-Val-CAC-1	0.117	1.1E-01		0.029	1.2E-03		0.103	1.9E-07	
GtsRNA-Val-CAC	GtsRNA-Val-CAC-2	Genomic tRNA-Val-CAC-2	0.114	4.6E-01		0.030	3.0E-05		0.103	7.4E-20	
GtsRNA-Val-CAC	GtsRNA-Val-CAC-3	Genomic tRNA-Val-CAC-3	0.138	1.5E-01		0.025	1.1E-03		0.126	2.2E-08	
GtsRNA-Val-CAC	GtsRNA-Val-CAC-4	Genomic tRNA-Val-CAC-4	0.069	6.3E-01		0.056	8.5E-12		0.058	2.0E-10	
GtsRNA-Val-CAC	GtsRNA-Val-CAC-5	Genomic tRNA-Val-CAC-5	0.385	9.6E-30	*	0.691	4.9E-32	*	0.361	4.6E-20	*
GtsRNA-Val-CAC	GtsRNA-Val-CAC-7	Genomic tRNA-Val-CAC-7	0.424	8.0E-39	*	0.826	3.8E-39	*	1.024	2.2E-23	*
GtsRNA-Val-TAC	GtsRNA-Val-TAC-1	Genomic tRNA-Val-TAC-1	0.649	8.0E-58	*	0.452	6.8E-37	*	0.510	5.4E-50	*
MtsRNA-Ala-TGC	MtsRNA-Ala-TGC-1	Mitochondrial tRNA-Ala-TGC-1	0.093	1.0E+00		0.102	1.0E-14		0.181	3.8E-09	
MtsRNA-Cys-GCA	MtsRNA-Cys-GCA-1	Mitochondrial tRNA-Cys-GCA-1	0.338	2.4E-04	*	0.434	1.0E-12	*	0.333	6.9E-01	
MtsRNA-Cys-GCA	MtsRNA-Cys-GCA-2	Mitochondrial tRNA-Cys-GCA-2	0.358	1.0E+00		0.263	7.0E-01		0.338	8.8E-01	
MtsRNA-Cys-GCA	MtsRNA-Cys-GCA-3	Mitochondrial tRNA-Cys-GCA-3	0.351	1.0E+00		0.388	1.0E-04	*	0.337	5.2E-01	
MtsRNA-Gln-TTG	MtsRNA-Gln-TTG-1	Mitochondrial tRNA-Gln-TTG-1	0.164	1.0E+00		0.384	1.1E-18	*	0.246	1.1E-03	*
MtsRNA-Gln-TTG	MtsRNA-Gln-TTG-2	Mitochondrial tRNA-Gln-TTG-2	0.140	7.0E-01		0.260	1.0E+00		0.155	1.0E+00	
MtsRNA-Gln-TTG	MtsRNA-Gln-TTG-3	Mitochondrial tRNA-Gln-TTG-3	0.162	1.0E+00		0.339	2.6E-08	*	0.195	8.2E-01	
MtsRNA-Glu-TTC	MtsRNA-Glu-TTC-1	Mitochondrial tRNA-Glu-TTC-1	0.190	6.1E-09		0.067	1.0E+00		0.256	3.1E-16	*
MtsRNA-Glu-TTC	MtsRNA-Glu-TTC-2	Mitochondrial tRNA-Glu-TTC-2	0.178	4.1E-07		0.056	1.0E+00		0.233	5.3E-11	*

			MEFs *vs.* intermediates			Intermediates *vs.* iPSCs			MEFs *vs.* iPSCs		
sncRNA family	sncRNA subfamily	Parental RNA	*CD*	Adjusted *P*		*CD*	Adjusted *P*		*CD*	Adjusted *P*	
MtsRNA-Glu-TTC	MtsRNA-Glu-TTC-3	Mitochondrial tRNA-Glu-TTC-3	0.014	1.0E+00		0.047	1.2E-09		0.041	1.0E-01	
MtsRNA-Gly-TCC	MtsRNA-Gly-TCC-1	Mitochondrial tRNA-Gly-TCC-1	0.035	1.2E-04		0.085	9.9E-01		0.118	4.7E-05	
MtsRNA-Gly-TCC	MtsRNA-Gly-TCC-2	Mitochondrial tRNA-Gly-TCC-2	0.034	1.9E-06		0.088	5.9E-01		0.116	5.9E-04	
MtsRNA-His-GTG	MtsRNA-His-GTG-1	Mitochondrial tRNA-His-GTG-1	0.171	8.3E-18		0.267	6.2E-06	*	0.429	1.1E-20	*
MtsRNA-His-GTG	MtsRNA-His-GTG-2	Mitochondrial tRNA-His-GTG-2	0.169	5.7E-18		0.266	5.9E-06	*	0.427	4.0E-22	*
MtsRNA-Ile-GAT	MtsRNA-Ile-GAT-1	Mitochondrial tRNA-Ile-GAT-1	0.178	7.3E-47		0.067	1.0E+00		0.179	5.8E-25	
MtsRNA-Ile-GAT	MtsRNA-Ile-GAT-2	Mitochondrial tRNA-Ile-GAT-2	0.178	7.3E-47		0.067	1.0E+00		0.179	5.8E-25	
MtsRNA-Leu-TAA	MtsRNA-Leu-TAA-1	Mitochondrial tRNA-Leu-TAA-1	0.514	8.2E-01		0.426	1.9E-11	*	0.863	5.1E-24	*
MtsRNA-Leu-TAG	MtsRNA-Leu-TAG-1	Mitochondrial tRNA-Leu-TAG-1	0.375	9.9E-01		0.141	8.3E-02		0.394	9.9E-01	
MtsRNA-Met-CAT	MtsRNA-Met-CAT-1	Mitochondrial tRNA-Met-CAT-1	0.353	8.7E-04	*	0.310	3.9E-03	*	0.590	7.1E-13	*
MtsRNA-Pro-TGG	MtsRNA-Pro-TGG-1	Mitochondrial tRNA-Pro-TGG-1	0.688	5.8E-19	*	0.088	1.0E+00		0.741	1.1E-22	*
MtsRNA-Pro-TGG	MtsRNA-Pro-TGG-2	Mitochondrial tRNA-Pro-TGG-2	0.680	2.9E-16	*	0.131	1.0E+00		0.750	2.7E-16	*
MtsRNA-Pro-TGG	MtsRNA-Pro-TGG-3	Mitochondrial tRNA-Pro-TGG-3	0.743	4.6E-19	*	0.195	1.0E+00		0.909	4.3E-19	*
MtsRNA-Ser-GCT	MtsRNA-Ser-GCT-1	Mitochondrial tRNA-Ser-GCT-1	0.138	2.4E-14		0.123	5.3E-01		0.144	4.7E-01	
MtsRNA-Ser-GCT	MtsRNA-Ser-GCT-2	Mitochondrial tRNA-Ser-GCT-2	0.177	5.1E-25		0.132	2.8E-01		0.179	6.7E-01	
MtsRNA-Ser-TGA	MtsRNA-Ser-TGA-1	Mitochondrial tRNA-Ser-TGA-1	0.089	8.8E-02		0.172	1.0E+00		0.116	8.7E-01	
MtsRNA-Thr-TGT	MtsRNA-Thr-TGT-1	Mitochondrial tRNA-Thr-TGT-1	0.262	6.1E-28	*	0.043	1.0E+00		0.300	1.4E-29	*
MtsRNA-Thr-TGT	MtsRNA-Thr-TGT-2	Mitochondrial tRNA-Thr-TGT-2	0.265	2.0E-29	*	0.042	9.8E-01		0.301	3.2E-31	*
MtsRNA-Thr-TGT	MtsRNA-Thr-TGT-3	Mitochondrial tRNA-Thr-TGT-3	0.270	1.8E-31	*	0.030	1.0E+00		0.295	1.4E-31	*
MtsRNA-Trp-TCA	MtsRNA-Trp-TCA-1	Mitochondrial tRNA-Trp-TCA-1	0.825	4.1E-18	*	0.329	1.7E-15	*	1.021	1.9E-24	*
MtsRNA-Trp-TCA	MtsRNA-Trp-TCA-2	Mitochondrial tRNA-Trp-TCA-2	0.772	2.3E-18	*	0.329	1.7E-15	*	0.966	7.4E-23	*
MtsRNA-Tyr-GTA	MtsRNA-Tyr-GTA-1	Mitochondrial tRNA-Tyr-GTA-1	0.130	1.1E-01		0.060	1.0E+00		0.170	2.4E-10	
MtsRNA-Val-TAC	MtsRNA-Val-TAC-1	Mitochondrial tRNA-Val-TAC-1	0.150	1.6E-28		0.167	2.2E-18		0.137	1.0E-04	
MtsRNA-Val-TAC	MtsRNA-Val-TAC-2	Mitochondrial tRNA-Val-TAC-2	0.206	5.2E-10	*	0.558	6.0E-55	*	0.509	2.2E-45	*
MtsRNA-Val-TAC	MtsRNA-Val-TAC-3	Mitochondrial tRNA-Val-TAC-3	0.165	1.1E-29		0.170	4.5E-14		0.140	3.5E-04	
rsRNA-4.5S	rsRNA-4.5S	4.5S rRNA	0.187	1.6E-40		0.024	1.0E+00		0.210	6.0E-81	*
rsRNA-5S	rsRNA-5S	5S rRNA	0.178	1.0E-01		0.375	7.0E-72	*	0.528	4.1E-76	*
rsRNA-5.8S	rsRNA-5.8S	5.8S rRNA	0.260	4.8E-02	*	0.431	4.7E-23	*	0.690	2.6E-88	*
rsRNA-12S	rsRNA-12S	12S rRNA	0.258	1.6E-17	*	0.206	6.4E-26	*	0.361	1.3E-32	*
rsRNA-16S	rsRNA-16S	16S rRNA	0.315	8.6E-75	*	0.179	4.8E-01		0.305	1.7E-155	*
rsRNA-18S	rsRNA-18S	18S rRNA	0.458	0.0E+00	*	0.432	5.3E-266	*	0.702	0.0E+00	*
rsRNA-28S	rsRNA-28S	28S rRNA	0.503	0.0E+00	*	0.554	0.0E+00	*	0.922	0.0E+00	*
rsRNA-45S	rsRNA-45S	45S rRNA	0.244	1.3E-207	*	1.139	0.0E+00	*	1.318	0.0E+00	*

Note - Differential fragmentation analysis was performed using *qMAP* Model 1. *: differentially fragmented parental RNAs; *CD*: coverage difference.
