# Supplementary material for: qMAP decodes RNA fragmentation dynamics in development and disease
Source: Mol Syst Biol. 2026 Jul 20;22(10):1626–49. doi: 10.1038/s44320-026-00237-2 (PMC13638987; doi:10.1038/s44320-026-00237-2)
Supplement: Supplementary file 3 [file 44320_2026_237_MOESM2_ESM.pdf]

Table EV2. Differential fragmentation analysis between controls, quiescent UC patients, and active UC patients

			Control *vs.* quiescent UC			Quiescent UC *vs.* active UC			Control *vs.* active UC		
sncRNA family	sncRNA subfamily	Parental RNA	*CD*	Adjusted *P* (Model 1)	Adjusted *P* (Model 2)	*CD*	Adjusted *P* (Model 1)	Adjusted *P* (Model 2)	*CD*	Adjusted *P* (Model 1)	Adjusted *P* (Model 2)
GtsRNA-Ala-AGC	GtsRNA-Ala-AGC-1	Genomic tRNA-Ala-AGC-1	0.129	1.2E-10	1.1E-01	0.039	1.0E+00	7.5E-01	0.127	5.7E-35	4.0E-02
GtsRNA-Ala-AGC	GtsRNA-Ala-AGC-10	Genomic tRNA-Ala-AGC-10	0.104	1.0E-20	3.1E-02	0.056	3.7E-01	4.4E-01	0.055	5.2E-01	2.7E-01
GtsRNA-Ala-AGC	GtsRNA-Ala-AGC-11	Genomic tRNA-Ala-AGC-11	0.229	1.1E-133	0.0E+00	0.192	9.0E-94	4.5E-03	0.194	1.5E-85	1.4E-03
GtsRNA-Ala-AGC	GtsRNA-Ala-AGC-12	Genomic tRNA-Ala-AGC-12	0.104	1.6E-22	2.5E-02	0.056	6.0E-01	4.4E-01	0.055	1.6E-01	2.7E-01
GtsRNA-Ala-AGC	GtsRNA-Ala-AGC-14	Genomic tRNA-Ala-AGC-14	0.103	6.5E-31	1.3E-02	0.055	1.0E+00	4.4E-01	0.054	6.2E-05	2.5E-01
GtsRNA-Ala-AGC	GtsRNA-Ala-AGC-15	Genomic tRNA-Ala-AGC-15	0.369	7.4E-237	0.0E+00	0.240	2.4E-58	4.5E-03	0.152	8.6E-37	1.9E-02
GtsRNA-Ala-AGC	GtsRNA-Ala-AGC-16	Genomic tRNA-Ala-AGC-16	0.031	1.1E-03	2.8E-01	0.016	1.0E+00	7.5E-01	0.023	2.6E-01	2.9E-01
GtsRNA-Ala-AGC	GtsRNA-Ala-AGC-2	Genomic tRNA-Ala-AGC-2	0.078	6.9E-04	4.7E-01	0.177	1.3E-37	1.3E-01	0.205	5.6E-32	2.7E-02
GtsRNA-Ala-AGC	GtsRNA-Ala-AGC-3	Genomic tRNA-Ala-AGC-3	0.078	1.6E-03	4.7E-01	0.176	3.9E-33	1.3E-01	0.205	2.7E-39	2.4E-02
GtsRNA-Ala-AGC	GtsRNA-Ala-AGC-4	Genomic tRNA-Ala-AGC-4	0.124	1.4E-10	3.1E-01	0.085	5.3E-01	5.0E-01	0.167	1.5E-14	8.3E-02
GtsRNA-Ala-AGC	GtsRNA-Ala-AGC-5	Genomic tRNA-Ala-AGC-5	0.086	1.4E-04	4.7E-01	0.198	1.5E-35	1.1E-01	0.195	6.9E-24	3.7E-02
GtsRNA-Ala-AGC	GtsRNA-Ala-AGC-6	Genomic tRNA-Ala-AGC-6	0.136	6.7E-02	2.4E-01	0.126	5.7E-12	2.3E-01	0.162	6.5E-39	3.8E-02
GtsRNA-Ala-AGC	GtsRNA-Ala-AGC-7	Genomic tRNA-Ala-AGC-7	0.090	2.2E-02	4.6E-01	0.209	5.0E-45	8.7E-02	0.199	2.1E-43	2.7E-02
GtsRNA-Ala-AGC	GtsRNA-Ala-AGC-8	Genomic tRNA-Ala-AGC-8	0.219	8.1E-125	8.9E-04	0.217	7.9E-109	1.4E-03	0.216	2.6E-132	0.0E+00
GtsRNA-Ala-AGC	GtsRNA-Ala-AGC-9	Genomic tRNA-Ala-AGC-9	0.316	3.0E-58	0.0E+00	0.203	4.9E-06	6.5E-02	0.120	7.8E-06	1.3E-01
GtsRNA-Ala-CGC	GtsRNA-Ala-CGC-1	Genomic tRNA-Ala-CGC-1	0.091	1.7E-02	4.3E-01	0.124	1.5E-05	3.1E-01	0.181	2.1E-28	6.0E-02
GtsRNA-Ala-CGC	GtsRNA-Ala-CGC-2	Genomic tRNA-Ala-CGC-2	0.089	2.5E-02	4.4E-01	0.124	5.6E-04	3.1E-01	0.180	1.2E-27	6.8E-02
GtsRNA-Ala-CGC	GtsRNA-Ala-CGC-3	Genomic tRNA-Ala-CGC-3	0.097	5.0E-04	4.0E-01	0.137	2.9E-21	2.7E-01	0.188	7.5E-48	3.9E-02
GtsRNA-Ala-CGC	GtsRNA-Ala-CGC-4	Genomic tRNA-Ala-CGC-4	0.141	1.1E-32	4.0E-02	0.118	9.2E-24	1.3E-01	0.101	2.2E-18	1.0E-01
GtsRNA-Ala-TGC	GtsRNA-Ala-TGC-1	Genomic tRNA-Ala-TGC-1	0.079	3.1E-02	4.8E-01	0.164	6.1E-12	1.7E-01	0.207	3.6E-48	3.3E-02
GtsRNA-Ala-TGC	GtsRNA-Ala-TGC-2	Genomic tRNA-Ala-TGC-2	0.090	1.8E-02	4.4E-01	0.124	6.1E-04	3.1E-01	0.180	1.8E-29	6.1E-02
GtsRNA-Ala-TGC	GtsRNA-Ala-TGC-3	Genomic tRNA-Ala-TGC-3	0.087	3.0E-03	4.6E-01	0.119	1.6E-02	3.3E-01	0.176	2.1E-17	6.6E-02
GtsRNA-Ala-TGC	GtsRNA-Ala-TGC-4	Genomic tRNA-Ala-TGC-4	0.082	1.1E-01	4.7E-01	0.129	4.5E-05	2.9E-01	0.182	4.7E-25	5.8E-02
GtsRNA-Ala-TGC	GtsRNA-Ala-TGC-5	Genomic tRNA-Ala-TGC-5	0.092	3.1E-02	4.5E-01	0.187	7.5E-31	1.4E-01	0.189	8.7E-33	4.1E-02
GtsRNA-Ala-TGC	GtsRNA-Ala-TGC-6	Genomic tRNA-Ala-TGC-6	0.107	1.7E-10	3.4E-01	0.057	3.0E-01	6.5E-01	0.152	1.6E-15	1.0E-01
GtsRNA-Ala-TGC	GtsRNA-Ala-TGC-7	Genomic tRNA-Ala-TGC-7	0.092	2.0E-03	4.5E-01	0.183	6.8E-16	1.4E-01	0.188	7.2E-26	4.1E-02
GtsRNA-Arg-ACG	GtsRNA-Arg-ACG-1	Genomic tRNA-Arg-ACG-1	0.055	8.4E-01	6.7E-01	0.208	6.2E-38	8.3E-02	0.214	4.4E-103	1.9E-02
GtsRNA-Arg-ACG	GtsRNA-Arg-ACG-2	Genomic tRNA-Arg-ACG-2	0.049	1.0E+00	7.4E-01	0.210	6.8E-34	7.9E-02	0.207	5.0E-76	2.4E-02
GtsRNA-Arg-CCG	GtsRNA-Arg-CCG-1	Genomic tRNA-Arg-CCG-1	0.063	1.0E+00	6.4E-01	0.134	2.8E-10	2.9E-01	0.085	4.9E-03	2.9E-01
GtsRNA-Arg-CCG	GtsRNA-Arg-CCG-2	Genomic tRNA-Arg-CCG-2	0.225	0.0E+00	2.1E-02	0.233	6.0E-148	1.2E-02	0.020	1.0E+00	8.8E-01
GtsRNA-Arg-CCT	GtsRNA-Arg-CCT-1	Genomic tRNA-Arg-CCT-1	0.083	5.0E-30	9.6E-02	0.072	3.5E-14	1.5E-01	0.080	5.0E-42	6.4E-02
GtsRNA-Arg-CCT	GtsRNA-Arg-CCT-2	Genomic tRNA-Arg-CCT-2	0.114	2.7E-22	2.2E-01	0.161	4.4E-34	4.5E-02	0.085	2.3E-08	2.0E-01
GtsRNA-Arg-CCT	GtsRNA-Arg-CCT-3	Genomic tRNA-Arg-CCT-3	0.091	1.1E-41	6.5E-02	0.074	7.8E-15	1.4E-01	0.085	6.1E-64	4.1E-02

sncRNA family	sncRNA subfamily	Parental RNA	Control *vs.* quiescent UC			Quiescent UC *vs.* active UC			Control *vs.* active UC		
			CD	Adjusted *P* (Model 1)	Adjusted *P* (Model 2)	*CD*	Adjusted *P* (Model 1)	Adjusted *P* (Model 2)	*CD*	Adjusted *P* (Model 1)	Adjusted *P* (Model 2)
GtsRNA-Arg-CCT	GtsRNA-Arg-CCT-4	Genomic tRNA-Arg-CCT-4	0.112	2.6E-22	2.7E-01	0.224	3.9E-148	3.8E-03	0.117	5.9E-46	8.5E-02
GtsRNA-Arg-CCT	GtsRNA-Arg-CCT-5	Genomic tRNA-Arg-CCT-5	0.159	2.8E-34	1.3E-01	0.199	4.4E-35	4.1E-02	0.340	3.3E-319	0.0E+00
GtsRNA-Arg-TCG	GtsRNA-Arg-TCG-1	Genomic tRNA-Arg-TCG-1	0.028	1.0E+00	8.3E-01	0.118	2.8E-11	3.7E-01	0.126	1.3E-28	2.0E-01
GtsRNA-Arg-TCG	GtsRNA-Arg-TCG-2	Genomic tRNA-Arg-TCG-2	0.103	4.8E-03	2.8E-01	0.081	6.5E-02	3.7E-01	0.166	8.2E-53	1.3E-02
GtsRNA-Arg-TCG	GtsRNA-Arg-TCG-3	Genomic tRNA-Arg-TCG-3	0.097	9.5E-02	5.1E-01	0.071	1.0E+00	6.8E-01	0.090	2.0E-03	4.0E-01
GtsRNA-Arg-TCG	GtsRNA-Arg-TCG-4	Genomic tRNA-Arg-TCG-4	0.106	1.6E-05	3.0E-01	0.085	8.2E-04	4.0E-01	0.174	6.6E-62	1.9E-02
GtsRNA-Arg-TCG	GtsRNA-Arg-TCG-5	Genomic tRNA-Arg-TCG-5	0.106	1.3E-05	3.5E-01	0.104	1.9E-08	2.9E-01	0.194	4.9E-101	1.3E-02
GtsRNA-Arg-TCG	GtsRNA-Arg-TCG-6	Genomic tRNA-Arg-TCG-6	0.099	8.9E-20	1.4E-01	0.131	1.3E-27	8.8E-02	0.088	2.7E-10	7.8E-02
GtsRNA-Arg-TCT	GtsRNA-Arg-TCT-1	Genomic tRNA-Arg-TCT-1	0.031	1.0E+00	5.3E-01	0.052	1.1E-01	4.6E-01	0.083	1.9E-60	1.0E-01
GtsRNA-Arg-TCT	GtsRNA-Arg-TCT-2	Genomic tRNA-Arg-TCT-2	0.039	1.0E+00	7.6E-01	0.052	1.0E+00	7.2E-01	0.035	1.0E+00	7.7E-01
GtsRNA-Arg-TCT	GtsRNA-Arg-TCT-3	Genomic tRNA-Arg-TCT-3	0.022	1.0E+00	8.6E-01	0.022	1.0E+00	8.8E-01	0.036	1.0E+00	7.4E-01
GtsRNA-Arg-TCT	GtsRNA-Arg-TCT-4	Genomic tRNA-Arg-TCT-4	0.346	1.6E-07	2.4E-01	0.505	2.1E-12	3.8E-02	0.248	2.3E-02	3.2E-01
GtsRNA-Arg-TCT	GtsRNA-Arg-TCT-5	Genomic tRNA-Arg-TCT-5	0.040	8.8E-01	6.7E-01	0.021	1.0E+00	7.3E-01	0.051	3.7E-02	3.4E-01
GtsRNA-Asn-GTT	GtsRNA-Asn-GTT-1	Genomic tRNA-Asn-GTT-1	0.195	3.2E-92	0.0E+00	0.132	6.4E-19	1.1E-01	0.068	2.4E-02	3.6E-01
GtsRNA-Asn-GTT	GtsRNA-Asn-GTT-11	Genomic tRNA-Asn-GTT-11	0.136	2.0E-01	4.1E-01	0.260	2.9E-31	1.2E-02	0.149	1.5E-14	7.9E-02
GtsRNA-Asn-GTT	GtsRNA-Asn-GTT-2	Genomic tRNA-Asn-GTT-2	0.161	4.7E-76	0.0E+00	0.099	6.9E-07	2.2E-01	0.069	3.2E-04	2.7E-01
GtsRNA-Asn-GTT	GtsRNA-Asn-GTT-3	Genomic tRNA-Asn-GTT-3	0.165	1.1E-68	3.1E-03	0.110	3.8E-11	1.9E-01	0.062	6.7E-02	3.6E-01
GtsRNA-Asn-GTT	GtsRNA-Asn-GTT-4	Genomic tRNA-Asn-GTT-4	0.201	5.6E-80	0.0E+00	0.133	1.2E-14	1.3E-01	0.074	2.2E-06	3.2E-01
GtsRNA-Asn-GTT	GtsRNA-Asn-GTT-5	Genomic tRNA-Asn-GTT-5	0.145	1.9E-34	3.1E-02	0.108	5.9E-09	1.3E-01	0.057	4.4E-04	4.0E-01
GtsRNA-Asn-GTT	GtsRNA-Asn-GTT-6	Genomic tRNA-Asn-GTT-6	0.174	7.6E-96	8.9E-04	0.116	1.0E-12	1.4E-01	0.063	4.6E-04	3.6E-01
GtsRNA-Asn-GTT	GtsRNA-Asn-GTT-7	Genomic tRNA-Asn-GTT-7	0.183	8.9E-31	1.6E-02	0.174	1.8E-21	7.5E-02	0.071	2.3E-04	3.1E-01
GtsRNA-Asn-GTT	GtsRNA-Asn-GTT-9	Genomic tRNA-Asn-GTT-9	0.300	3.1E-26	2.5E-02	0.278	1.5E-30	3.8E-02	0.070	2.1E-01	7.0E-01
GtsRNA-Asp-GTC	GtsRNA-Asp-GTC-1	Genomic tRNA-Asp-GTC-1	0.276	1.9E-224	8.7E-03	0.174	8.9E-31	1.7E-01	0.449	0.0E+00	0.0E+00
GtsRNA-Asp-GTC	GtsRNA-Asp-GTC-2	Genomic tRNA-Asp-GTC-2	0.299	6.1E-242	6.0E-03	0.159	3.5E-22	2.2E-01	0.457	0.0E+00	0.0E+00
GtsRNA-Asp-GTC	GtsRNA-Asp-GTC-3	Genomic tRNA-Asp-GTC-3	0.040	6.7E-12	4.0E-01	0.024	1.0E+00	7.4E-01	0.031	9.5E-09	2.9E-01
GtsRNA-Cys-GCA	GtsRNA-Cys-GCA-1	Genomic tRNA-Cys-GCA-1	0.034	8.4E-01	5.4E-01	0.100	3.7E-120	8.4E-04	0.133	0.0E+00	0.0E+00
GtsRNA-Cys-GCA	GtsRNA-Cys-GCA-10	Genomic tRNA-Cys-GCA-10	0.014	1.0E+00	7.4E-01	0.053	7.9E-59	8.4E-04	0.056	3.8E-145	1.4E-03
GtsRNA-Cys-GCA	GtsRNA-Cys-GCA-11	Genomic tRNA-Cys-GCA-11	0.095	4.2E-01	4.0E-01	0.118	3.7E-19	2.4E-01	0.153	2.0E-50	4.0E-02
GtsRNA-Cys-GCA	GtsRNA-Cys-GCA-12	Genomic tRNA-Cys-GCA-12	0.066	6.0E-01	5.6E-01	0.200	1.7E-68	0.0E+00	0.194	3.6E-70	1.4E-03
GtsRNA-Cys-GCA	GtsRNA-Cys-GCA-13	Genomic tRNA-Cys-GCA-13	0.034	9.9E-01	5.3E-01	0.100	4.5E-126	2.0E-03	0.132	0.0E+00	0.0E+00
GtsRNA-Cys-GCA	GtsRNA-Cys-GCA-14	Genomic tRNA-Cys-GCA-14	0.039	1.3E-01	4.7E-01	0.104	3.4E-100	1.4E-03	0.142	0.0E+00	0.0E+00
GtsRNA-Cys-GCA	GtsRNA-Cys-GCA-15	Genomic tRNA-Cys-GCA-15	0.029	7.5E-01	5.6E-01	0.091	1.5E-121	0.0E+00	0.118	0.0E+00	0.0E+00
GtsRNA-Cys-GCA	GtsRNA-Cys-GCA-16	Genomic tRNA-Cys-GCA-16	0.109	7.1E-06	3.2E-01	0.186	2.5E-42	8.2E-02	0.282	1.3E-127	7.7E-04
GtsRNA-Cys-GCA	GtsRNA-Cys-GCA-17	Genomic tRNA-Cys-GCA-17	0.086	1.8E-01	4.3E-01	0.112	3.0E-19	2.2E-01	0.138	3.9E-53	3.4E-02
GtsRNA-Cys-GCA	GtsRNA-Cys-GCA-18	Genomic tRNA-Cys-GCA-18	0.073	3.8E-01	4.9E-01	0.151	2.0E-42	9.6E-02	0.158	2.6E-58	2.4E-02

sncRNA family	sncRNA subfamily	Parental RNA	Control *vs.* quiescent UC			Quiescent UC *vs.* active UC			Control *vs.* active UC		
			CD	Adjusted *P* (Model 1)	Adjusted *P* (Model 2)	*CD*	Adjusted *P* (Model 1)	Adjusted *P* (Model 2)	*CD*	Adjusted *P* (Model 1)	Adjusted *P* (Model 2)
GtsRNA-Cys-GCA	GtsRNA-Cys-GCA-19	Genomic tRNA-Cys-GCA-19	0.028	8.3E-01	5.6E-01	0.090	5.0E-108	0.0E+00	0.116	0.0E+00	0.0E+00
GtsRNA-Cys-GCA	GtsRNA-Cys-GCA-2	Genomic tRNA-Cys-GCA-2	0.037	1.1E-01	4.3E-01	0.086	2.9E-108	4.5E-03	0.123	0.0E+00	0.0E+00
GtsRNA-Cys-GCA	GtsRNA-Cys-GCA-20	Genomic tRNA-Cys-GCA-20	0.086	6.6E-02	4.3E-01	0.133	1.5E-30	1.4E-01	0.151	5.6E-58	2.4E-02
GtsRNA-Cys-GCA	GtsRNA-Cys-GCA-21	Genomic tRNA-Cys-GCA-21	0.076	1.0E+00	4.9E-01	0.110	9.7E-30	2.7E-01	0.075	3.1E-14	3.4E-01
GtsRNA-Cys-GCA	GtsRNA-Cys-GCA-22	Genomic tRNA-Cys-GCA-22	0.074	9.3E-02	3.0E-01	0.137	1.4E-30	2.0E-02	0.119	7.2E-44	9.0E-03
GtsRNA-Cys-GCA	GtsRNA-Cys-GCA-23	Genomic tRNA-Cys-GCA-23	0.096	3.2E-04	2.5E-01	0.182	1.5E-66	1.2E-02	0.095	9.5E-23	9.1E-02
GtsRNA-Cys-GCA	GtsRNA-Cys-GCA-3	Genomic tRNA-Cys-GCA-3	0.099	1.0E-03	3.9E-01	0.180	5.2E-33	5.6E-02	0.244	4.7E-100	0.0E+00
GtsRNA-Cys-GCA	GtsRNA-Cys-GCA-4	Genomic tRNA-Cys-GCA-4	0.039	9.5E-07	4.0E-01	0.082	4.3E-141	3.1E-03	0.120	0.0E+00	0.0E+00
GtsRNA-Cys-GCA	GtsRNA-Cys-GCA-5	Genomic tRNA-Cys-GCA-5	0.166	2.4E-32	5.6E-02	0.145	6.8E-20	1.1E-01	0.243	5.4E-115	0.0E+00
GtsRNA-Cys-GCA	GtsRNA-Cys-GCA-6	Genomic tRNA-Cys-GCA-6	0.051	1.1E-12	2.5E-01	0.067	1.9E-107	5.2E-03	0.112	0.0E+00	0.0E+00
GtsRNA-Cys-GCA	GtsRNA-Cys-GCA-7	Genomic tRNA-Cys-GCA-7	0.097	8.1E-01	4.0E-01	0.140	4.3E-28	1.4E-01	0.182	2.2E-69	1.1E-02
GtsRNA-Cys-GCA	GtsRNA-Cys-GCA-8	Genomic tRNA-Cys-GCA-8	0.037	1.0E+00	5.1E-01	0.112	3.4E-123	8.4E-04	0.147	0.0E+00	0.0E+00
GtsRNA-Cys-GCA	GtsRNA-Cys-GCA-9	Genomic tRNA-Cys-GCA-9	0.032	8.9E-01	5.5E-01	0.102	1.4E-165	1.4E-03	0.134	0.0E+00	0.0E+00
GtsRNA-Gln-CTG	GtsRNA-Gln-CTG-1	Genomic tRNA-Gln-CTG-1	0.101	5.8E-10	3.0E-01	0.232	3.5E-74	3.4E-03	0.196	6.7E-179	6.7E-03
GtsRNA-Gln-CTG	GtsRNA-Gln-CTG-2	Genomic tRNA-Gln-CTG-2	0.094	1.5E-05	3.5E-01	0.238	3.2E-71	3.8E-03	0.198	3.5E-164	5.6E-03
GtsRNA-Gln-CTG	GtsRNA-Gln-CTG-3	Genomic tRNA-Gln-CTG-3	0.107	1.4E-08	3.0E-01	0.256	5.9E-109	4.5E-03	0.208	9.4E-167	1.9E-03
GtsRNA-Gln-CTG	GtsRNA-Gln-CTG-4	Genomic tRNA-Gln-CTG-4	0.107	2.0E-07	3.0E-01	0.256	1.4E-84	2.6E-03	0.208	4.1E-204	5.9E-03
GtsRNA-Gln-CTG	GtsRNA-Gln-CTG-5	Genomic tRNA-Gln-CTG-5	0.107	2.3E-11	2.2E-01	0.205	2.5E-61	3.1E-03	0.147	1.4E-108	7.7E-03
GtsRNA-Gln-CTG	GtsRNA-Gln-CTG-6	Genomic tRNA-Gln-CTG-6	0.107	2.3E-34	1.3E-01	0.160	5.0E-132	3.7E-02	0.077	2.0E-27	1.7E-01
GtsRNA-Gln-CTG	GtsRNA-Gln-CTG-7	Genomic tRNA-Gln-CTG-7	0.105	5.6E-06	2.5E-01	0.254	2.4E-139	0.0E+00	0.157	1.8E-197	1.4E-03
GtsRNA-Gln-TTG	GtsRNA-Gln-TTG-1	Genomic tRNA-Gln-TTG-1	0.114	4.1E-13	2.8E-01	0.223	1.8E-49	2.2E-02	0.157	4.2E-34	4.9E-02
GtsRNA-Gln-TTG	GtsRNA-Gln-TTG-2	Genomic tRNA-Gln-TTG-2	0.119	2.5E-09	2.2E-01	0.214	2.2E-46	1.3E-02	0.129	8.3E-21	6.7E-02
GtsRNA-Gln-TTG	GtsRNA-Gln-TTG-3	Genomic tRNA-Gln-TTG-3	0.128	1.4E-37	9.3E-02	0.189	6.7E-78	1.9E-02	0.092	1.9E-15	1.2E-01
GtsRNA-Gln-TTG	GtsRNA-Gln-TTG-4	Genomic tRNA-Gln-TTG-4	0.122	4.0E-12	2.2E-01	0.194	1.7E-63	6.8E-02	0.077	6.2E-03	3.2E-01
GtsRNA-Glu-CTC	GtsRNA-Glu-CTC-1	Genomic tRNA-Glu-CTC-1	0.005	1.0E+00	8.2E-01	0.021	6.7E-21	1.2E-01	0.019	2.8E-61	3.0E-02
GtsRNA-Glu-CTC	GtsRNA-Glu-CTC-2	Genomic tRNA-Glu-CTC-2	0.005	1.0E+00	8.0E-01	0.018	8.6E-21	1.3E-01	0.018	3.5E-34	2.6E-02
GtsRNA-Glu-TTC	GtsRNA-Glu-TTC-1	Genomic tRNA-Glu-TTC-1	0.041	1.1E-16	2.7E-01	0.065	8.7E-83	6.8E-02	0.041	2.0E-119	8.3E-02
GtsRNA-Glu-TTC	GtsRNA-Glu-TTC-2	Genomic tRNA-Glu-TTC-2	0.051	2.9E-25	2.0E-01	0.075	1.0E-103	4.7E-02	0.040	1.5E-45	1.0E-01
GtsRNA-Glu-TTC	GtsRNA-Glu-TTC-3	Genomic tRNA-Glu-TTC-3	0.009	4.4E-02	4.0E-01	0.014	5.7E-24	1.9E-01	0.006	1.7E-03	4.2E-01
GtsRNA-Glu-TTC	GtsRNA-Glu-TTC-4	Genomic tRNA-Glu-TTC-4	0.010	5.2E-02	4.7E-01	0.018	1.6E-21	1.9E-01	0.008	2.0E-05	3.6E-01
GtsRNA-Gly-CCC	GtsRNA-Gly-CCC-1	Genomic tRNA-Gly-CCC-1	0.006	9.5E-01	4.8E-01	0.007	2.1E-06	4.3E-01	0.006	5.5E-42	3.9E-01
GtsRNA-Gly-CCC	GtsRNA-Gly-CCC-2	Genomic tRNA-Gly-CCC-2	0.123	1.9E-29	2.8E-01	0.071	1.0E-06	4.3E-01	0.060	9.8E-01	4.7E-01
GtsRNA-Gly-CCC	GtsRNA-Gly-CCC-3	Genomic tRNA-Gly-CCC-3	0.002	1.0E+00	6.2E-01	0.008	1.3E-25	1.4E-03	0.007	6.1E-85	1.9E-03
GtsRNA-Gly-GCC	GtsRNA-Gly-GCC-1	Genomic tRNA-Gly-GCC-1	0.008	3.6E-02	4.0E-01	0.008	4.6E-07	4.0E-01	0.007	8.2E-39	3.2E-01
GtsRNA-Gly-GCC	GtsRNA-Gly-GCC-2	Genomic tRNA-Gly-GCC-2	0.008	8.4E-01	4.3E-01	0.008	1.6E-01	4.3E-01	0.009	1.8E-36	2.9E-01

			Control *vs.* quiescent UC			Quiescent UC *vs.* active UC			Control *vs.* active UC		
sncRNA family	sncRNA subfamily	Parental RNA	*CD*	Adjusted *P* (Model 1)	Adjusted *P* (Model 2)	*CD*	Adjusted *P* (Model 1)	Adjusted *P* (Model 2)	*CD*	Adjusted *P* (Model 1)	Adjusted *P* (Model 2)
GtsRNA-Gly-GCC	GtsRNA-Gly-GCC-3	Genomic tRNA-Gly-GCC-3	0.008	1.9E-03	4.0E-01	0.007	3.2E-10	4.2E-01	0.007	7.6E-64	3.4E-01
GtsRNA-Gly-GCC	GtsRNA-Gly-GCC-4	Genomic tRNA-Gly-GCC-4	0.054	6.3E-14	3.7E-01	0.249	6.8E-124	0.0E+00	0.224	8.4E-206	0.0E+00
GtsRNA-Gly-GCC	GtsRNA-Gly-GCC-5	Genomic tRNA-Gly-GCC-5	0.007	7.7E-01	4.1E-01	0.007	2.6E-18	4.2E-01	0.006	1.1E-47	3.8E-01
GtsRNA-Gly-TCC	GtsRNA-Gly-TCC-1	Genomic tRNA-Gly-TCC-1	0.124	1.7E-07	4.0E-01	0.118	1.0E-04	4.1E-01	0.036	1.0E+00	7.8E-01
GtsRNA-Gly-TCC	GtsRNA-Gly-TCC-2	Genomic tRNA-Gly-TCC-2	0.128	4.2E-10	3.9E-01	0.110	1.6E-02	4.3E-01	0.042	1.0E+00	7.3E-01
GtsRNA-Gly-TCC	GtsRNA-Gly-TCC-3	Genomic tRNA-Gly-TCC-3	0.128	6.7E-12	4.0E-01	0.105	2.6E-05	4.4E-01	0.046	1.0E+00	7.1E-01
GtsRNA-Gly-TCC	GtsRNA-Gly-TCC-4	Genomic tRNA-Gly-TCC-4	0.182	2.4E-65	1.8E-01	0.103	7.0E-03	4.4E-01	0.108	1.6E-13	3.1E-01
GtsRNA-His-GTG	GtsRNA-His-GTG-1	Genomic tRNA-His-GTG-1	0.042	9.5E-19	1.3E-01	0.061	1.8E-145	1.4E-03	0.072	1.8E-276	7.7E-04
GtsRNA-His-GTG	GtsRNA-His-GTG-2	Genomic tRNA-His-GTG-2	0.053	1.4E-05	1.9E-01	0.082	4.7E-49	5.0E-02	0.041	7.6E-16	1.7E-01
GtsRNA-Ile-AAT	GtsRNA-Ile-AAT-1	Genomic tRNA-Ile-AAT-1	0.123	9.1E-12	1.5E-01	0.097	1.1E-03	3.2E-01	0.086	1.7E-13	2.6E-01
GtsRNA-Ile-AAT	GtsRNA-Ile-AAT-2	Genomic tRNA-Ile-AAT-2	0.086	2.2E-04	3.8E-01	0.064	1.0E+00	6.5E-01	0.122	2.1E-24	1.0E-01
GtsRNA-Ile-AAT	GtsRNA-Ile-AAT-3	Genomic tRNA-Ile-AAT-3	0.079	1.2E-01	4.1E-01	0.069	8.0E-01	5.6E-01	0.115	2.6E-19	1.2E-01
GtsRNA-Ile-AAT	GtsRNA-Ile-AAT-4	Genomic tRNA-Ile-AAT-4	0.093	3.0E-05	3.3E-01	0.064	9.6E-01	6.1E-01	0.113	2.2E-22	1.4E-01
GtsRNA-Ile-AAT	GtsRNA-Ile-AAT-5	Genomic tRNA-Ile-AAT-5	0.080	2.5E-02	4.0E-01	0.056	1.0E+00	6.9E-01	0.102	3.0E-20	1.9E-01
GtsRNA-Ile-AAT	GtsRNA-Ile-AAT-6	Genomic tRNA-Ile-AAT-6	0.103	4.9E-04	4.0E-01	0.121	6.0E-10	2.5E-01	0.116	6.2E-21	1.2E-01
GtsRNA-Ile-AAT	GtsRNA-Ile-AAT-7	Genomic tRNA-Ile-AAT-7	0.077	3.0E-03	4.1E-01	0.046	1.0E+00	7.9E-01	0.099	5.7E-11	2.0E-01
GtsRNA-Ile-AAT	GtsRNA-Ile-AAT-8	Genomic tRNA-Ile-AAT-8	0.078	7.0E-02	4.0E-01	0.046	1.0E+00	7.4E-01	0.096	5.2E-06	2.1E-01
GtsRNA-Ile-AAT	GtsRNA-Ile-AAT-9	Genomic tRNA-Ile-AAT-9	0.132	7.9E-02	5.1E-01	0.247	2.3E-36	8.8E-02	0.154	2.3E-12	2.3E-01
GtsRNA-Ile-GAT	GtsRNA-Ile-GAT-1	Genomic tRNA-Ile-GAT-1	0.144	1.0E+00	4.8E-01	0.193	2.8E-07	1.9E-01	0.201	7.9E-27	3.0E-02
GtsRNA-Ile-TAT	GtsRNA-Ile-TAT-1	Genomic tRNA-Ile-TAT-1	0.182	2.2E-04	3.0E-01	0.222	6.7E-04	1.2E-01	0.173	2.8E-31	6.8E-02
GtsRNA-Ile-TAT	GtsRNA-Ile-TAT-2	Genomic tRNA-Ile-TAT-2	0.157	1.1E-04	3.5E-01	0.188	2.2E-04	1.4E-01	0.159	2.4E-31	8.7E-02
GtsRNA-Ile-TAT	GtsRNA-Ile-TAT-3	Genomic tRNA-Ile-TAT-3	0.187	6.3E-06	2.8E-01	0.220	5.7E-05	1.3E-01	0.171	1.1E-27	7.7E-02
GtsRNA-iMet-CAT	GtsRNA-iMet-CAT-1	Genomic tRNA-iMet-CAT-1	0.015	9.6E-01	6.0E-01	0.018	4.0E-01	4.4E-01	0.025	2.4E-03	2.4E-01
GtsRNA-iMet-CAT	GtsRNA-iMet-CAT-2	Genomic tRNA-iMet-CAT-2-1	0.015	9.6E-01	5.7E-01	0.020	5.7E-04	3.9E-01	0.026	9.0E-12	2.1E-01
GtsRNA-Leu-AAG	GtsRNA-Leu-AAG-1	Genomic tRNA-Leu-AAG-1	0.222	9.2E-207	3.1E-02	0.086	5.5E-25	4.2E-01	0.137	1.9E-11	1.7E-01
GtsRNA-Leu-AAG	GtsRNA-Leu-AAG-2	Genomic tRNA-Leu-AAG-2	0.222	3.0E-209	3.1E-02	0.087	2.1E-19	4.2E-01	0.136	3.5E-09	1.6E-01
GtsRNA-Leu-AAG	GtsRNA-Leu-AAG-3	Genomic tRNA-Leu-AAG-3	0.224	1.8E-165	3.1E-02	0.088	1.2E-21	4.1E-01	0.137	2.3E-08	1.7E-01
GtsRNA-Leu-AAG	GtsRNA-Leu-AAG-4	Genomic tRNA-Leu-AAG-4	0.136	8.4E-145	9.4E-02	0.068	1.3E-45	3.9E-01	0.069	8.3E-04	3.4E-01
GtsRNA-Leu-CAA	GtsRNA-Leu-CAA-1	Genomic tRNA-Leu-CAA-1	0.102	1.1E-10	1.9E-01	0.256	2.8E-119	3.4E-03	0.155	1.6E-43	2.4E-02
GtsRNA-Leu-CAA	GtsRNA-Leu-CAA-2	Genomic tRNA-Leu-CAA-2	0.103	9.6E-25	1.8E-01	0.255	5.0E-135	4.9E-03	0.155	4.5E-58	2.7E-02
GtsRNA-Leu-CAA	GtsRNA-Leu-CAA-3	Genomic tRNA-Leu-CAA-3	0.101	8.9E-21	1.9E-01	0.253	2.8E-128	3.4E-03	0.155	7.2E-52	2.3E-02
GtsRNA-Leu-CAA	GtsRNA-Leu-CAA-4	Genomic tRNA-Leu-CAA-4	0.103	1.6E-38	1.7E-01	0.255	1.5E-144	5.6E-03	0.155	2.2E-49	2.5E-02
GtsRNA-Leu-CAA	GtsRNA-Leu-CAA-5	Genomic tRNA-Leu-CAA-5	0.183	4.8E-16	1.1E-01	0.267	4.1E-70	1.9E-02	0.153	1.2E-38	9.0E-03
GtsRNA-Leu-CAA	GtsRNA-Leu-CAA-6	Genomic tRNA-Leu-CAA-6	0.220	4.3E-98	2.4E-03	0.203	6.8E-90	1.6E-02	0.028	1.8E-01	7.1E-01
GtsRNA-Leu-CAG	GtsRNA-Leu-CAG-1	Genomic tRNA-Leu-CAG-1	0.142	4.1E-48	5.6E-02	0.201	9.2E-92	1.4E-02	0.088	1.7E-32	2.0E-01

			Control *vs.* quiescent UC			Quiescent UC *vs.* active UC			Control *vs.* active UC		
sncRNA family	sncRNA subfamily	Parental RNA	*CD*	Adjusted *P* (Model 1)	Adjusted *P* (Model 2)	*CD*	Adjusted *P* (Model 1)	Adjusted *P* (Model 2)	*CD*	Adjusted *P* (Model 1)	Adjusted *P* (Model 2)
GtsRNA-Leu-CAG	GtsRNA-Leu-CAG-2	Genomic tRNA-Leu-CAG-2	0.152	6.3E-55	4.1E-02	0.209	1.6E-117	1.2E-02	0.087	4.4E-20	2.0E-01
GtsRNA-Leu-TAA	GtsRNA-Leu-TAA-1	Genomic tRNA-Leu-TAA-1	0.065	1.0E+00	6.8E-01	0.037	1.0E+00	9.4E-01	0.070	3.0E-02	4.6E-01
GtsRNA-Leu-TAA	GtsRNA-Leu-TAA-2	Genomic tRNA-Leu-TAA-2	0.082	8.9E-03	2.8E-01	0.068	1.2E-04	4.0E-01	0.037	1.0E+00	6.8E-01
GtsRNA-Leu-TAA	GtsRNA-Leu-TAA-3	Genomic tRNA-Leu-TAA-3	0.061	1.0E+00	7.3E-01	0.065	1.0E+00	7.0E-01	0.029	1.0E+00	9.1E-01
GtsRNA-Leu-TAA	GtsRNA-Leu-TAA-4	Genomic tRNA-Leu-TAA-4	0.100	1.0E+00	8.8E-01	0.150	7.0E-01	6.5E-01	0.152	6.1E-01	4.7E-01
GtsRNA-Leu-TAG	GtsRNA-Leu-TAG-1	Genomic tRNA-Leu-TAG-1	0.223	2.3E-177	3.1E-02	0.084	1.8E-16	4.3E-01	0.139	5.3E-12	1.6E-01
GtsRNA-Leu-TAG	GtsRNA-Leu-TAG-2	Genomic tRNA-Leu-TAG-2	0.200	1.8E-141	4.9E-02	0.095	1.5E-34	3.8E-01	0.106	4.3E-02	2.5E-01
GtsRNA-Leu-TAG	GtsRNA-Leu-TAG-3	Genomic tRNA-Leu-TAG-3	0.204	8.6E-102	4.0E-02	0.092	2.4E-04	3.8E-01	0.112	7.0E-12	2.3E-01
GtsRNA-Lys-CTT	GtsRNA-Lys-CTT-1	Genomic tRNA-Lys-CTT-1	0.006	1.0E+00	8.0E-01	0.033	4.7E-153	0.0E+00	0.032	1.0E-241	7.7E-03
GtsRNA-Lys-CTT	GtsRNA-Lys-CTT-10	Genomic tRNA-Lys-CTT-10	0.036	1.3E-01	3.0E-01	0.043	2.9E-30	1.9E-01	0.072	4.7E-66	3.0E-03
GtsRNA-Lys-CTT	GtsRNA-Lys-CTT-11	Genomic tRNA-Lys-CTT-11	0.044	5.6E-05	2.4E-01	0.039	3.2E-02	2.9E-01	0.071	2.5E-45	9.0E-03
GtsRNA-Lys-CTT	GtsRNA-Lys-CTT-2	Genomic tRNA-Lys-CTT-2	0.006	8.8E-01	8.0E-01	0.037	7.2E-107	0.0E+00	0.032	8.9E-246	9.0E-03
GtsRNA-Lys-CTT	GtsRNA-Lys-CTT-3	Genomic tRNA-Lys-CTT-3	0.021	7.2E-01	5.9E-01	0.081	1.7E-149	8.4E-04	0.062	0.0E+00	3.0E-03
GtsRNA-Lys-CTT	GtsRNA-Lys-CTT-4	Genomic tRNA-Lys-CTT-4	0.006	1.0E+00	8.1E-01	0.033	6.9E-121	8.4E-04	0.032	1.2E-262	7.1E-03
GtsRNA-Lys-CTT	GtsRNA-Lys-CTT-5	Genomic tRNA-Lys-CTT-5	0.015	7.2E-01	3.5E-01	0.038	2.0E-173	0.0E+00	0.024	3.3E-241	7.7E-04
GtsRNA-Lys-CTT	GtsRNA-Lys-CTT-7	Genomic tRNA-Lys-CTT-7	0.016	1.0E+00	6.6E-01	0.077	2.9E-118	0.0E+00	0.061	2.7E-194	0.0E+00
GtsRNA-Lys-CTT	GtsRNA-Lys-CTT-8	Genomic tRNA-Lys-CTT-8	0.329	5.5E-208	1.7E-03	0.637	0.0E+00	0.0E+00	0.320	2.9E-142	7.7E-04
GtsRNA-Lys-CTT	GtsRNA-Lys-CTT-9	Genomic tRNA-Lys-CTT-9	0.051	3.3E-85	8.9E-04	0.068	1.7E-40	9.3E-03	0.023	9.2E-03	4.5E-01
GtsRNA-Lys-TTT	GtsRNA-Lys-TTT-1	Genomic tRNA-Lys-TTT-1	0.028	8.4E-01	4.3E-01	0.048	2.0E-42	1.9E-01	0.069	1.7E-128	1.1E-02
GtsRNA-Lys-TTT	GtsRNA-Lys-TTT-11	Genomic tRNA-Lys-TTT-11	0.032	9.7E-05	2.8E-01	0.035	8.6E-63	2.9E-01	0.055	7.0E-110	1.6E-02
GtsRNA-Lys-TTT	GtsRNA-Lys-TTT-2	Genomic tRNA-Lys-TTT-2	0.035	8.9E-01	6.7E-01	0.153	2.6E-124	1.4E-02	0.161	2.3E-178	7.7E-04
GtsRNA-Lys-TTT	GtsRNA-Lys-TTT-3	Genomic tRNA-Lys-TTT-3	0.049	1.1E-01	6.4E-01	0.154	1.1E-22	2.2E-02	0.145	9.6E-48	1.7E-02
GtsRNA-Lys-TTT	GtsRNA-Lys-TTT-4	Genomic tRNA-Lys-TTT-4	0.021	1.0E+00	8.7E-01	0.164	9.3E-38	1.7E-02	0.158	1.5E-66	1.0E-02
GtsRNA-Lys-TTT	GtsRNA-Lys-TTT-5	Genomic tRNA-Lys-TTT-5	0.032	9.6E-01	7.7E-01	0.151	9.1E-41	2.3E-02	0.150	7.9E-92	1.3E-02
GtsRNA-Lys-TTT	GtsRNA-Lys-TTT-6	Genomic tRNA-Lys-TTT-6	0.041	8.2E-01	2.7E-01	0.040	4.7E-74	2.8E-01	0.076	8.0E-235	2.5E-03
GtsRNA-Lys-TTT	GtsRNA-Lys-TTT-7	Genomic tRNA-Lys-TTT-7	0.073	6.3E-37	3.7E-02	0.058	4.7E-05	1.2E-01	0.061	4.4E-60	5.8E-02
GtsRNA-Met-CAT	GtsRNA-Met-CAT-1	Genomic tRNA-Met-CAT-1	0.024	1.0E+00	7.6E-01	0.144	2.9E-75	1.2E-02	0.153	8.1E-135	3.5E-03
GtsRNA-Met-CAT	GtsRNA-Met-CAT-2	Genomic tRNA-Met-CAT-2	0.054	8.2E-01	3.7E-01	0.046	7.1E-01	4.0E-01	0.035	7.0E-02	4.5E-01
GtsRNA-Met-CAT	GtsRNA-Met-CAT-3	Genomic tRNA-Met-CAT-3	0.090	1.4E-05	2.7E-01	0.066	4.2E-01	4.9E-01	0.066	1.7E-14	1.5E-01
GtsRNA-Met-CAT	GtsRNA-Met-CAT-4	Genomic tRNA-Met-CAT-4	0.060	7.1E-04	2.3E-01	0.045	5.0E-01	3.9E-01	0.037	1.5E-05	3.7E-01
GtsRNA-Met-CAT	GtsRNA-Met-CAT-5	Genomic tRNA-Met-CAT-5	0.063	1.4E-09	1.7E-01	0.045	4.8E-01	3.7E-01	0.033	1.7E-02	4.6E-01
GtsRNA-Met-CAT	GtsRNA-Met-CAT-6	Genomic tRNA-Met-CAT-6	0.032	1.0E+00	7.7E-01	0.078	9.3E-08	1.4E-01	0.051	8.9E-01	3.6E-01
GtsRNA-Met-CAT	GtsRNA-Met-CAT-7	Genomic tRNA-Met-CAT-7	0.061	1.6E-01	6.0E-01	0.076	5.0E-02	2.8E-01	0.029	2.1E-01	8.0E-01
GtsRNA-Phe-GAA	GtsRNA-Phe-GAA-1	Genomic tRNA-Phe-GAA-1	0.051	1.0E-02	4.5E-01	0.127	4.7E-123	8.4E-04	0.079	6.4E-54	6.1E-02
GtsRNA-Phe-GAA	GtsRNA-Phe-GAA-2	Genomic tRNA-Phe-GAA-2	0.047	2.5E-01	4.7E-01	0.100	1.5E-99	5.2E-03	0.061	6.7E-23	1.7E-01

sncRNA family	sncRNA subfamily	Parental RNA	Control *vs.* quiescent UC			Quiescent UC *vs.* active UC			Control *vs.* active UC		
			CD	Adjusted *P* (Model 1)	Adjusted *P* (Model 2)	*CD*	Adjusted *P* (Model 1)	Adjusted *P* (Model 2)	*CD*	Adjusted *P* (Model 1)	Adjusted *P* (Model 2)
GtsRNA-Phe-GAA	GtsRNA-Phe-GAA-3	Genomic tRNA-Phe-GAA-3	0.046	2.7E-02	4.7E-01	0.098	1.7E-93	3.4E-03	0.056	1.2E-32	1.8E-01
GtsRNA-Phe-GAA	GtsRNA-Phe-GAA-4	Genomic tRNA-Phe-GAA-4	0.046	3.0E-01	3.9E-01	0.061	3.6E-11	1.1E-01	0.040	8.7E-19	2.4E-01
GtsRNA-Phe-GAA	GtsRNA-Phe-GAA-6	Genomic tRNA-Phe-GAA-6	0.118	9.5E-01	4.7E-01	0.122	1.8E-03	4.0E-01	0.072	1.4E-06	3.6E-01
GtsRNA-Pro-AGG	GtsRNA-Pro-AGG-1	Genomic tRNA-Pro-AGG-1	0.075	9.1E-01	5.7E-01	0.460	2.4E-319	0.0E+00	0.426	4.6E-288	7.7E-04
GtsRNA-Pro-AGG	GtsRNA-Pro-AGG-2	Genomic tRNA-Pro-AGG-2	0.098	4.4E-61	4.7E-01	0.453	5.4E-312	0.0E+00	0.424	0.0E+00	0.0E+00
GtsRNA-Pro-CGG	GtsRNA-Pro-CGG-1	Genomic tRNA-Pro-CGG-1	0.096	1.5E-51	4.9E-01	0.453	0.0E+00	0.0E+00	0.428	0.0E+00	7.7E-04
GtsRNA-Pro-CGG	GtsRNA-Pro-CGG-2	Genomic tRNA-Pro-CGG-2	0.077	5.4E-10	5.7E-01	0.460	0.0E+00	0.0E+00	0.430	0.0E+00	0.0E+00
GtsRNA-Pro-TGG	GtsRNA-Pro-TGG-1	Genomic tRNA-Pro-TGG-1	0.123	4.1E-64	3.9E-01	0.416	7.5E-313	0.0E+00	0.343	1.0E-188	5.9E-03
GtsRNA-Pro-TGG	GtsRNA-Pro-TGG-2	Genomic tRNA-Pro-TGG-2	0.103	1.5E-39	4.7E-01	0.405	6.2E-288	0.0E+00	0.337	1.6E-122	5.1E-03
GtsRNA-Pro-TGG	GtsRNA-Pro-TGG-3	Genomic tRNA-Pro-TGG-3	0.098	2.9E-49	4.7E-01	0.447	0.0E+00	0.0E+00	0.422	1.2E-276	0.0E+00
GtsRNA-SeC-TCA	GtsRNA-SeC-TCA-1	Genomic tRNA-SeC-TCA-1	0.166	8.6E-38	9.4E-02	0.272	1.4E-133	0.0E+00	0.133	2.7E-76	8.7E-02
GtsRNA-Ser-AGA	GtsRNA-Ser-AGA-1	Genomic tRNA-Ser-AGA-1	0.310	0.0E+00	0.0E+00	0.258	2.6E-86	1.2E-02	0.072	1.4E-20	4.5E-01
GtsRNA-Ser-AGA	GtsRNA-Ser-AGA-2	Genomic tRNA-Ser-AGA-2	0.297	3.9E-286	0.0E+00	0.255	8.4E-69	1.2E-02	0.066	1.6E-16	4.7E-01
GtsRNA-Ser-AGA	GtsRNA-Ser-AGA-3	Genomic tRNA-Ser-AGA-3	0.289	0.0E+00	0.0E+00	0.254	8.5E-74	1.2E-02	0.064	4.6E-21	4.9E-01
GtsRNA-Ser-AGA	GtsRNA-Ser-AGA-4	Genomic tRNA-Ser-AGA-4	0.279	5.0E-170	8.9E-04	0.281	3.9E-80	7.7E-03	0.051	9.4E-01	6.1E-01
GtsRNA-Ser-CGA	GtsRNA-Ser-CGA-1	Genomic tRNA-Ser-CGA-1	0.181	2.2E-04	3.8E-01	0.140	7.9E-01	4.4E-01	0.278	1.1E-84	1.9E-02
GtsRNA-Ser-CGA	GtsRNA-Ser-CGA-2	Genomic tRNA-Ser-CGA-2	0.123	1.0E+00	6.4E-01	0.191	2.7E-04	2.8E-01	0.271	2.4E-56	3.2E-02
GtsRNA-Ser-CGA	GtsRNA-Ser-CGA-3	Genomic tRNA-Ser-CGA-3	0.143	9.9E-01	6.0E-01	0.361	1.5E-45	4.1E-02	0.320	6.3E-72	6.3E-03
GtsRNA-Ser-CGA	GtsRNA-Ser-CGA-4	Genomic tRNA-Ser-CGA-4	0.151	5.3E-10	1.3E-01	0.059	1.0E+00	6.8E-01	0.137	3.8E-12	8.9E-02
GtsRNA-Ser-GCT	GtsRNA-Ser-GCT-1	Genomic tRNA-Ser-GCT-1	0.074	1.1E-10	4.9E-01	0.151	3.4E-28	1.2E-01	0.172	4.7E-126	1.6E-02
GtsRNA-Ser-GCT	GtsRNA-Ser-GCT-2	Genomic tRNA-Ser-GCT-2	0.142	9.2E-12	2.4E-01	0.189	2.9E-76	7.7E-03	0.132	8.0E-29	1.2E-01
GtsRNA-Ser-GCT	GtsRNA-Ser-GCT-3	Genomic tRNA-Ser-GCT-3	0.066	3.0E-06	4.8E-01	0.142	1.3E-37	8.7E-02	0.154	2.6E-102	1.9E-02
GtsRNA-Ser-GCT	GtsRNA-Ser-GCT-4	Genomic tRNA-Ser-GCT-4	0.068	2.6E-08	4.7E-01	0.144	3.2E-33	8.3E-02	0.158	5.3E-90	1.3E-02
GtsRNA-Ser-GCT	GtsRNA-Ser-GCT-5	Genomic tRNA-Ser-GCT-5	0.070	1.3E-07	4.7E-01	0.148	4.7E-29	8.2E-02	0.162	5.2E-121	9.8E-03
GtsRNA-Ser-GCT	GtsRNA-Ser-GCT-6	Genomic tRNA-Ser-GCT-6	0.068	2.7E-22	2.5E-02	0.092	3.0E-49	7.7E-03	0.055	4.0E-23	7.0E-02
GtsRNA-Ser-TGA	GtsRNA-Ser-TGA-1	Genomic tRNA-Ser-TGA-1	0.193	5.1E-13	1.3E-01	0.221	5.6E-31	3.7E-02	0.036	1.0E+00	8.3E-01
GtsRNA-Ser-TGA	GtsRNA-Ser-TGA-2	Genomic tRNA-Ser-TGA-2	0.310	0.0E+00	0.0E+00	0.258	7.6E-68	1.0E-02	0.072	1.7E-21	4.4E-01
GtsRNA-Ser-TGA	GtsRNA-Ser-TGA-3	Genomic tRNA-Ser-TGA-3	0.296	2.2E-304	8.9E-04	0.255	4.3E-104	1.3E-02	0.066	1.3E-09	4.8E-01
GtsRNA-Ser-TGA	GtsRNA-Ser-TGA-4	Genomic tRNA-Ser-TGA-4	0.086	1.0E-47	2.2E-01	0.129	3.4E-71	1.4E-02	0.073	4.4E-11	1.9E-01
GtsRNA-Thr-AGT	GtsRNA-Thr-AGT-1	Genomic tRNA-Thr-AGT-1	0.263	8.6E-66	0.0E+00	0.195	2.9E-36	7.7E-03	0.089	4.4E-07	1.7E-01
GtsRNA-Thr-AGT	GtsRNA-Thr-AGT-2	Genomic tRNA-Thr-AGT-2	0.266	5.0E-65	0.0E+00	0.172	1.3E-20	2.3E-02	0.110	2.7E-13	1.0E-01
GtsRNA-Thr-AGT	GtsRNA-Thr-AGT-3	Genomic tRNA-Thr-AGT-3	0.340	2.8E-50	0.0E+00	0.238	2.8E-20	3.4E-02	0.120	5.6E-11	1.2E-01
GtsRNA-Thr-AGT	GtsRNA-Thr-AGT-4	Genomic tRNA-Thr-AGT-4	0.254	4.5E-19	1.6E-02	0.172	1.3E-03	2.9E-01	0.134	1.5E-11	7.8E-02
GtsRNA-Thr-AGT	GtsRNA-Thr-AGT-5	Genomic tRNA-Thr-AGT-5	0.263	2.2E-81	8.9E-04	0.188	2.2E-21	1.5E-02	0.094	1.1E-11	1.4E-01
GtsRNA-Thr-AGT	GtsRNA-Thr-AGT-6	Genomic tRNA-Thr-AGT-6	0.282	3.0E-104	0.0E+00	0.176	1.7E-17	2.4E-02	0.111	1.0E-10	8.7E-02

sncRNA family	sncRNA subfamily	Parental RNA	Control *vs.* quiescent UC			Quiescent UC *vs.* active UC			Control *vs.* active UC		
			CD	Adjusted *P* (Model 1)	Adjusted *P* (Model 2)	*CD*	Adjusted *P* (Model 1)	Adjusted *P* (Model 2)	*CD*	Adjusted *P* (Model 1)	Adjusted *P* (Model 2)
GtsRNA-Thr-CGT	GtsRNA-Thr-CGT-1	Genomic tRNA-Thr-CGT-1	0.337	1.4E-41	0.0E+00	0.231	4.3E-07	1.4E-01	0.136	1.9E-10	1.0E-01
GtsRNA-Thr-CGT	GtsRNA-Thr-CGT-2	Genomic tRNA-Thr-CGT-2	0.192	8.5E-78	8.9E-04	0.037	1.0E+00	9.1E-01	0.185	2.7E-197	7.7E-04
GtsRNA-Thr-CGT	GtsRNA-Thr-CGT-3	Genomic tRNA-Thr-CGT-3	0.269	1.2E-87	8.9E-04	0.169	9.6E-14	2.5E-02	0.111	1.6E-24	1.0E-01
GtsRNA-Thr-CGT	GtsRNA-Thr-CGT-4	Genomic tRNA-Thr-CGT-4	0.174	1.9E-78	1.7E-03	0.030	1.0E+00	9.2E-01	0.183	5.7E-193	0.0E+00
GtsRNA-Thr-TGT	GtsRNA-Thr-TGT-1	Genomic tRNA-Thr-TGT-1	0.288	2.8E-11	3.1E-02	0.146	9.2E-01	6.3E-01	0.242	5.5E-94	1.9E-03
GtsRNA-Thr-TGT	GtsRNA-Thr-TGT-2	Genomic tRNA-Thr-TGT-2	0.123	1.1E-01	4.0E-01	0.208	2.8E-31	7.9E-02	0.133	2.3E-49	1.8E-01
GtsRNA-Thr-TGT	GtsRNA-Thr-TGT-3	Genomic tRNA-Thr-TGT-3	0.067	4.6E-01	4.7E-01	0.116	6.0E-31	1.8E-01	0.092	3.3E-34	2.3E-01
GtsRNA-Thr-TGT	GtsRNA-Thr-TGT-4	Genomic tRNA-Thr-TGT-4	0.136	2.1E-12	1.9E-01	0.155	2.1E-41	1.1E-01	0.106	2.4E-49	2.2E-01
GtsRNA-Thr-TGT	GtsRNA-Thr-TGT-5	Genomic tRNA-Thr-TGT-5	0.101	1.0E-07	2.3E-01	0.190	6.0E-88	1.2E-02	0.109	1.6E-36	7.9E-02
GtsRNA-Thr-TGT	GtsRNA-Thr-TGT-6	Genomic tRNA-Thr-TGT-6	0.148	5.4E-23	2.0E-01	0.076	8.0E-01	4.6E-01	0.092	1.4E-10	3.1E-01
GtsRNA-Trp-CCA	GtsRNA-Trp-CCA-1	Genomic tRNA-Trp-CCA-1	0.040	1.0E+00	7.9E-01	0.176	5.2E-17	1.4E-01	0.201	6.0E-63	2.7E-02
GtsRNA-Trp-CCA	GtsRNA-Trp-CCA-2	Genomic tRNA-Trp-CCA-2	0.055	9.3E-01	6.4E-01	0.135	1.5E-08	2.8E-01	0.146	3.2E-21	1.2E-01
GtsRNA-Trp-CCA	GtsRNA-Trp-CCA-3	Genomic tRNA-Trp-CCA-3	0.044	1.0E+00	7.3E-01	0.160	1.4E-11	1.7E-01	0.198	2.3E-85	2.7E-02
GtsRNA-Trp-CCA	GtsRNA-Trp-CCA-4	Genomic tRNA-Trp-CCA-4	0.043	1.0E+00	7.3E-01	0.165	2.2E-13	1.5E-01	0.205	1.8E-84	2.0E-02
GtsRNA-Trp-CCA	GtsRNA-Trp-CCA-5	Genomic tRNA-Trp-CCA-5	0.030	1.0E+00	8.5E-01	0.195	3.3E-22	1.3E-01	0.199	9.9E-67	4.1E-02
GtsRNA-Tyr-GTA	GtsRNA-Tyr-GTA-1	Genomic tRNA-Tyr-GTA-1	0.062	3.7E-01	5.7E-01	0.162	6.7E-15	9.0E-02	0.184	2.9E-43	2.4E-02
GtsRNA-Tyr-GTA	GtsRNA-Tyr-GTA-2	Genomic tRNA-Tyr-GTA-2	0.052	5.9E-01	5.7E-01	0.123	4.2E-14	1.4E-01	0.134	9.4E-35	3.8E-02
GtsRNA-Tyr-GTA	GtsRNA-Tyr-GTA-3	Genomic tRNA-Tyr-GTA-3	0.101	3.0E-01	4.7E-01	0.207	7.1E-15	8.7E-02	0.293	3.2E-54	1.3E-02
GtsRNA-Tyr-GTA	GtsRNA-Tyr-GTA-4	Genomic tRNA-Tyr-GTA-4	0.123	9.5E-13	1.8E-01	0.068	9.3E-01	4.7E-01	0.175	3.5E-66	2.3E-02
GtsRNA-Tyr-GTA	GtsRNA-Tyr-GTA-5	Genomic tRNA-Tyr-GTA-5	0.079	1.3E-13	2.2E-01	0.046	1.0E+00	6.5E-01	0.119	1.4E-44	3.1E-02
GtsRNA-Tyr-GTA	GtsRNA-Tyr-GTA-6	Genomic tRNA-Tyr-GTA-6	0.210	1.3E-29	8.6E-02	0.093	4.2E-01	3.9E-01	0.289	1.6E-123	7.5E-03
GtsRNA-Tyr-GTA	GtsRNA-Tyr-GTA-7	Genomic tRNA-Tyr-GTA-7	0.112	4.2E-18	1.7E-01	0.063	4.1E-01	4.0E-01	0.156	1.6E-47	2.1E-02
GtsRNA-Tyr-GTA	GtsRNA-Tyr-GTA-8	Genomic tRNA-Tyr-GTA-8	0.054	9.6E-01	6.4E-01	0.165	1.4E-13	6.8E-02	0.159	1.9E-28	4.1E-02
GtsRNA-Tyr-GTA	GtsRNA-Tyr-GTA-9	Genomic tRNA-Tyr-GTA-9	0.067	9.1E-01	6.2E-01	0.077	9.3E-01	5.1E-01	0.063	7.7E-01	3.6E-01
GtsRNA-Val-AAC	GtsRNA-Val-AAC-1	Genomic tRNA-Val-AAC-1	0.005	7.9E-03	4.1E-01	0.005	5.2E-01	4.2E-01	0.005	6.9E-05	2.9E-01
GtsRNA-Val-AAC	GtsRNA-Val-AAC-2	Genomic tRNA-Val-AAC-2	0.004	8.3E-01	4.7E-01	0.003	1.0E+00	6.6E-01	0.004	2.1E-05	3.6E-01
GtsRNA-Val-AAC	GtsRNA-Val-AAC-3	Genomic tRNA-Val-AAC-3	0.004	3.7E-01	4.6E-01	0.005	5.2E-03	3.9E-01	0.004	9.3E-08	2.9E-01
GtsRNA-Val-AAC	GtsRNA-Val-AAC-4	Genomic tRNA-Val-AAC-4	0.005	2.9E-06	4.0E-01	0.005	3.3E-01	4.1E-01	0.005	1.5E-06	3.0E-01
GtsRNA-Val-AAC	GtsRNA-Val-AAC-5	Genomic tRNA-Val-AAC-5	0.003	1.0E+00	5.6E-01	0.004	7.0E-01	6.2E-01	0.004	1.0E+00	4.6E-01
GtsRNA-Val-AAC	GtsRNA-Val-AAC-6	Genomic tRNA-Val-AAC-6	0.063	4.2E-17	2.0E-01	0.096	3.1E-27	4.6E-02	0.056	4.3E-05	2.7E-01
GtsRNA-Val-CAC	GtsRNA-Val-CAC-1	Genomic tRNA-Val-CAC-1	0.005	1.5E-03	4.0E-01	0.005	2.0E-02	3.9E-01	0.005	4.0E-13	2.9E-01
GtsRNA-Val-CAC	GtsRNA-Val-CAC-2	Genomic tRNA-Val-CAC-2	0.006	9.9E-02	4.7E-01	0.008	4.3E-05	3.8E-01	0.005	1.5E-04	3.6E-01
GtsRNA-Val-CAC	GtsRNA-Val-CAC-3	Genomic tRNA-Val-CAC-3	0.077	6.3E-32	1.1E-01	0.064	1.1E-53	1.6E-01	0.062	1.6E-41	1.1E-01
GtsRNA-Val-CAC	GtsRNA-Val-CAC-4	Genomic tRNA-Val-CAC-4	0.004	3.5E-01	4.5E-01	0.005	1.6E-01	3.8E-01	0.005	1.5E-08	2.9E-01
GtsRNA-Val-CAC	GtsRNA-Val-CAC-5	Genomic tRNA-Val-CAC-5	0.005	2.3E-03	4.0E-01	0.005	5.2E-01	3.8E-01	0.005	1.6E-03	2.9E-01

sncRNA family	sncRNA subfamily	Parental RNA	Control *vs.* quiescent UC			Quiescent UC *vs.* active UC			Control *vs.* active UC		
			CD	Adjusted *P* (Model 1)	Adjusted *P* (Model 2)	*CD*	Adjusted *P* (Model 1)	Adjusted *P* (Model 2)	*CD*	Adjusted *P* (Model 1)	Adjusted *P* (Model 2)
GtsRNA-Val-CAC	GtsRNA-Val-CAC-6	Genomic tRNA-Val-CAC-6	0.005	2.1E-03	4.0E-01	0.005	4.5E-02	4.0E-01	0.005	2.7E-08	2.8E-01
GtsRNA-Val-TAC	GtsRNA-Val-TAC-1	Genomic tRNA-Val-TAC-1	0.201	9.2E-30	1.1E-01	0.247	3.4E-49	6.8E-02	0.055	1.0E+00	5.9E-01
GtsRNA-Val-TAC	GtsRNA-Val-TAC-2	Genomic tRNA-Val-TAC-2	0.207	1.5E-40	9.6E-02	0.250	7.5E-85	6.9E-02	0.053	9.9E-01	6.3E-01
GtsRNA-Val-TAC	GtsRNA-Val-TAC-3	Genomic tRNA-Val-TAC-3	0.223	1.1E-57	7.0E-02	0.251	1.4E-53	6.7E-02	0.045	1.0E+00	6.8E-01
GtsRNA-Val-TAC	GtsRNA-Val-TAC-4	Genomic tRNA-Val-TAC-4	0.187	6.9E-64	8.1E-02	0.133	1.2E-18	2.6E-01	0.095	4.5E-15	2.9E-01
MtsRNA-Ala-TGC	MtsRNA-Ala-TGC-1	Mitochondrial tRNA-Ala-TGC-1	0.084	1.0E+00	8.5E-01	0.349	6.7E-26	3.8E-02	0.318	1.0E-52	2.7E-02
MtsRNA-Arg-TCG	MtsRNA-Arg-TCG-1	Mitochondrial tRNA-Arg-TCG-1	0.106	1.7E-04	3.2E-01	0.171	1.3E-20	1.0E-01	0.071	7.5E-03	3.5E-01
MtsRNA-Asn-GTT	MtsRNA-Asn-GTT-1	Mitochondrial tRNA-Asn-GTT-1	0.050	1.0E+00	7.7E-01	0.129	3.6E-06	2.0E-01	0.090	3.3E-27	1.3E-01
MtsRNA-Asn-GTT	MtsRNA-Asn-GTT-2	Mitochondrial tRNA-Asn-GTT-2	0.049	1.0E+00	7.8E-01	0.152	6.3E-10	1.4E-01	0.109	4.7E-35	1.0E-01
MtsRNA-Asp-GTC	MtsRNA-Asp-GTC-1	Mitochondrial tRNA-Asp-GTC-1	0.095	9.3E-01	6.2E-01	0.199	2.7E-31	3.3E-02	0.108	2.6E-10	2.3E-01
MtsRNA-Asp-GTC	MtsRNA-Asp-GTC-2	Mitochondrial tRNA-Asp-GTC-2	0.088	1.0E+00	7.2E-01	0.193	8.8E-35	3.4E-02	0.121	1.0E-11	1.8E-01
MtsRNA-Cys-GCA	MtsRNA-Cys-GCA-1	Mitochondrial tRNA-Cys-GCA-1	0.103	7.5E-01	5.0E-01	0.190	7.7E-18	1.6E-01	0.104	1.8E-03	3.6E-01
MtsRNA-Cys-GCA	MtsRNA-Cys-GCA-2	Mitochondrial tRNA-Cys-GCA-2	0.078	1.0E+00	6.6E-01	0.184	4.7E-12	1.8E-01	0.129	4.4E-13	2.5E-01
MtsRNA-Gln-TTG	MtsRNA-Gln-TTG-1	Mitochondrial tRNA-Gln-TTG-1	0.144	6.4E-191	0.0E+00	0.172	1.9E-201	0.0E+00	0.047	5.4E-18	6.1E-02
MtsRNA-Glu-TTC	MtsRNA-Glu-TTC-1	Mitochondrial tRNA-Glu-TTC-1	0.055	3.6E-191	0.0E+00	0.072	3.5E-111	3.1E-03	0.025	6.3E-02	2.4E-01
MtsRNA-Gly-TCC	MtsRNA-Gly-TCC-1	Mitochondrial tRNA-Gly-TCC-1	0.268	4.0E-22	1.0E-01	0.402	1.1E-68	7.7E-03	0.202	2.6E-12	1.0E-01
MtsRNA-His-GTG	MtsRNA-His-GTG-1	Mitochondrial tRNA-His-GTG-1	0.066	1.9E-59	9.3E-02	0.117	3.9E-184	2.0E-03	0.058	1.3E-59	6.0E-02
MtsRNA-His-GTG	MtsRNA-His-GTG-2	Mitochondrial tRNA-His-GTG-2	0.071	2.0E-35	1.3E-01	0.134	1.1E-183	1.4E-03	0.071	2.9E-75	4.6E-02
MtsRNA-Ile-GAT	MtsRNA-Ile-GAT-1	Mitochondrial tRNA-Ile-GAT-1	0.131	1.1E-05	2.4E-01	0.203	2.2E-29	2.3E-02	0.112	5.7E-10	1.0E-01
MtsRNA-Ile-GAT	MtsRNA-Ile-GAT-2	Mitochondrial tRNA-Ile-GAT-2	0.188	4.6E-05	1.7E-01	0.252	1.9E-19	3.1E-02	0.105	5.7E-03	2.3E-01
MtsRNA-Leu-TAA	MtsRNA-Leu-TAA-1	Mitochondrial tRNA-Leu-TAA-1	0.079	1.6E-12	3.9E-01	0.120	1.6E-35	9.3E-02	0.061	1.9E-07	2.9E-01
MtsRNA-Leu-TAG	MtsRNA-Leu-TAG-1	Mitochondrial tRNA-Leu-TAG-1	0.119	7.9E-05	1.9E-01	0.126	6.8E-05	1.2E-01	0.022	1.0E+00	7.6E-01
MtsRNA-Lys-TTT	MtsRNA-Lys-TTT-1	Mitochondrial tRNA-Lys-TTT-1	0.094	1.5E-17	2.5E-01	0.108	1.1E-18	1.4E-01	0.059	8.5E-05	3.3E-01
MtsRNA-Lys-TTT	MtsRNA-Lys-TTT-2	Mitochondrial tRNA-Lys-TTT-2	0.089	7.6E-15	3.5E-01	0.104	2.4E-10	2.4E-01	0.064	1.1E-05	2.9E-01
MtsRNA-Met-CAT	MtsRNA-Met-CAT-1	Mitochondrial tRNA-Met-CAT-1	0.023	5.2E-79	1.6E-02	0.038	4.6E-225	8.4E-04	0.019	4.9E-109	6.8E-02
MtsRNA-Phe-GAA	MtsRNA-Phe-GAA-1	Mitochondrial tRNA-Phe-GAA-1	0.077	1.9E-17	3.7E-01	0.064	3.4E-18	4.5E-01	0.048	2.3E-04	5.1E-01
MtsRNA-Pro-TGG	MtsRNA-Pro-TGG-1	Mitochondrial tRNA-Pro-TGG-1	0.182	2.5E-167	2.4E-03	0.249	8.0E-246	0.0E+00	0.106	1.3E-52	2.4E-02
MtsRNA-Ser-GCT	MtsRNA-Ser-GCT-1	Mitochondrial tRNA-Ser-GCT-1	0.071	3.6E-108	8.9E-04	0.112	7.4E-263	0.0E+00	0.045	1.2E-94	2.4E-02
MtsRNA-Ser-TGA	MtsRNA-Ser-TGA-1	Mitochondrial tRNA-Ser-TGA-1	0.098	1.5E-05	3.0E-01	0.244	2.1E-96	1.6E-02	0.155	1.1E-55	4.4E-02
MtsRNA-Thr-TGT	MtsRNA-Thr-TGT-1	Mitochondrial tRNA-Thr-TGT-1	0.056	1.0E+00	7.0E-01	0.103	1.8E-03	3.7E-01	0.052	1.0E+00	6.8E-01
MtsRNA-Trp-TCA	MtsRNA-Trp-TCA-1	Mitochondrial tRNA-Trp-TCA-1	0.056	1.0E+00	6.9E-01	0.076	1.0E+00	6.1E-01	0.111	3.1E-14	1.6E-01
MtsRNA-Tyr-GTA	MtsRNA-Tyr-GTA-1	Mitochondrial tRNA-Tyr-GTA-1	0.005	2.7E-02	4.8E-01	0.016	6.0E-28	1.6E-02	0.020	2.4E-86	7.7E-04
MtsRNA-Tyr-GTA	MtsRNA-Tyr-GTA-2	Mitochondrial tRNA-Tyr-GTA-2	0.004	1.0E+00	6.8E-01	0.016	8.9E-48	1.2E-02	0.019	3.9E-68	0.0E+00
MtsRNA-Val-TAC	MtsRNA-Val-TAC-1	Mitochondrial tRNA-Val-TAC-1	0.180	6.9E-181	0.0E+00	0.403	0.0E+00	0.0E+00	0.228	7.9E-205	1.9E-03
rsRNA-5S	rsRNA-5S	5S rRNA	0.105	4.9E-281	4.9E-02	0.163	0.0E+00	3.8E-03	0.071	3.3E-55	2.4E-01

sncRNA family	sncRNA subfamily	Parental RNA	Control *vs.* quiescent UC			Quiescent UC *vs.* active UC			Control *vs.* active UC		
			CD	Adjusted *P* (Model 1)	Adjusted *P* (Model 2)	*CD*	Adjusted *P* (Model 1)	Adjusted *P* (Model 2)	*CD*	Adjusted *P* (Model 1)	Adjusted *P* (Model 2)
rsRNA-5.8S	rsRNA-5.8S	5.8S rRNA	0.148	5.7E-89	2.8E-01	0.176	1.5E-287	2.3E-01	0.282	0.0E+00	7.7E-04
rsRNA-12S	rsRNA-12S	12S rRNA	0.565	0.0E+00	0.0E+00	0.702	0.0E+00	0.0E+00	0.259	1.3E-301	2.5E-02
rsRNA-16S	rsRNA-16S	16S rRNA	0.454	0.0E+00	0.0E+00	0.656	0.0E+00	0.0E+00	0.297	0.0E+00	0.0E+00
rsRNA-18S	rsRNA-18S	18S rRNA	0.635	0.0E+00	0.0E+00	1.019	0.0E+00	0.0E+00	0.411	0.0E+00	0.0E+00
rsRNA-28S	rsRNA-28S	28S rRNA	0.741	0.0E+00	0.0E+00	1.151	0.0E+00	0.0E+00	0.476	0.0E+00	0.0E+00
rsRNA-45S	rsRNA-45S	45S rRNA	0.603	0.0E+00	0.0E+00	0.875	0.0E+00	0.0E+00	0.471	0.0E+00	0.0E+00

Note - Differential fragmentation analysis was performed using *qMAP* Model 1 and Model 2. *CD*: coverage difference.
