# Supplementary material for: qMAP decodes RNA fragmentation dynamics in development and disease
Source: Mol Syst Biol. 2026 Jul 20;22(10):1626–49. doi: 10.1038/s44320-026-00237-2 (PMC13638987; doi:10.1038/s44320-026-00237-2)
Supplement: Supplementary file 4 [file 44320_2026_237_MOESM3_ESM.pdf]

Table EV3. Differential fragmentation analysis between ES controls, MS controls, and MS RIF patients

			ES controls *vs.* MS controls		ES controls *vs.* MS patients			MS controls *vs.* MS patients		
sncRNA family	sncRNA subfamily	Parental RNA	*CD*	Adjusted *P*	*CD*	Adjusted *P*		*CD*	Adjusted *P*	
GtsRNA-Ala-AGC	GtsRNA-Ala-AGC-11	Genomic tRNA-Ala-AGC-11	0.022	9.9E-01	0.052	5.4E-01		0.063	3.1E-01	
GtsRNA-Ala-AGC	GtsRNA-Ala-AGC-15	Genomic tRNA-Ala-AGC-15	0.023	9.9E-01	0.046	5.1E-01		0.054	2.3E-01	
GtsRNA-Ala-AGC	GtsRNA-Ala-AGC-2	Genomic tRNA-Ala-AGC-2	0.013	9.9E-01	0.038	8.0E-02		0.037	2.6E-02	
GtsRNA-Ala-AGC	GtsRNA-Ala-AGC-3	Genomic tRNA-Ala-AGC-3	0.008	9.9E-01	0.038	1.8E-02		0.032	3.4E-02	
GtsRNA-Ala-AGC	GtsRNA-Ala-AGC-4	Genomic tRNA-Ala-AGC-4	0.006	9.9E-01	0.053	7.5E-02		0.052	3.7E-02	
GtsRNA-Ala-AGC	GtsRNA-Ala-AGC-5	Genomic tRNA-Ala-AGC-5	0.015	9.9E-01	0.068	1.2E-02		0.068	5.9E-03	
GtsRNA-Ala-AGC	GtsRNA-Ala-AGC-7	Genomic tRNA-Ala-AGC-7	0.022	9.9E-01	0.067	3.9E-02		0.069	5.9E-03	
GtsRNA-Ala-AGC	GtsRNA-Ala-AGC-8	Genomic tRNA-Ala-AGC-8	0.017	9.9E-01	0.049	5.4E-01		0.058	3.1E-01	
GtsRNA-Ala-AGC	GtsRNA-Ala-AGC-9	Genomic tRNA-Ala-AGC-9	0.055	9.6E-01	0.034	6.6E-01		0.056	1.6E-01	
GtsRNA-Ala-CGC	GtsRNA-Ala-CGC-1	Genomic tRNA-Ala-CGC-1	0.005	9.9E-01	0.052	8.4E-03		0.052	6.0E-03	
GtsRNA-Ala-CGC	GtsRNA-Ala-CGC-2	Genomic tRNA-Ala-CGC-2	0.006	9.9E-01	0.050	9.4E-03		0.051	6.8E-03	
GtsRNA-Ala-CGC	GtsRNA-Ala-CGC-3	Genomic tRNA-Ala-CGC-3	0.006	9.9E-01	0.048	1.6E-02		0.049	5.4E-03	
GtsRNA-Ala-CGC	GtsRNA-Ala-CGC-4	Genomic tRNA-Ala-CGC-4	0.012	9.9E-01	0.080	8.0E-02		0.080	7.0E-02	
GtsRNA-Ala-TGC	GtsRNA-Ala-TGC-1	Genomic tRNA-Ala-TGC-1	0.005	9.9E-01	0.050	8.9E-03		0.046	6.8E-03	
GtsRNA-Ala-TGC	GtsRNA-Ala-TGC-2	Genomic tRNA-Ala-TGC-2	0.005	9.9E-01	0.053	8.4E-03		0.052	6.3E-03	
GtsRNA-Ala-TGC	GtsRNA-Ala-TGC-3	Genomic tRNA-Ala-TGC-3	0.008	9.9E-01	0.050	8.6E-03		0.053	5.4E-03	
GtsRNA-Ala-TGC	GtsRNA-Ala-TGC-4	Genomic tRNA-Ala-TGC-4	0.006	9.9E-01	0.049	8.4E-03		0.048	5.9E-03	
GtsRNA-Ala-TGC	GtsRNA-Ala-TGC-5	Genomic tRNA-Ala-TGC-5	0.006	9.9E-01	0.084	7.5E-03		0.079	5.4E-03	
GtsRNA-Ala-TGC	GtsRNA-Ala-TGC-6	Genomic tRNA-Ala-TGC-6	0.004	9.9E-01	0.047	1.2E-02		0.044	5.4E-03	
GtsRNA-Ala-TGC	GtsRNA-Ala-TGC-7	Genomic tRNA-Ala-TGC-7	0.006	9.9E-01	0.084	7.5E-03		0.079	5.9E-03	
GtsRNA-Arg-ACG	GtsRNA-Arg-ACG-1	Genomic tRNA-Arg-ACG-1	0.009	9.9E-01	0.034	1.9E-01		0.036	1.1E-01	
GtsRNA-Arg-ACG	GtsRNA-Arg-ACG-2	Genomic tRNA-Arg-ACG-2	0.011	9.9E-01	0.034	2.1E-01		0.042	4.3E-02	
GtsRNA-Asp-GTC	GtsRNA-Asp-GTC-1	Genomic tRNA-Asp-GTC-1	0.087	9.9E-01	0.218	2.8E-02	*	0.268	5.1E-03	*
GtsRNA-Asp-GTC	GtsRNA-Asp-GTC-2	Genomic tRNA-Asp-GTC-2	0.096	9.9E-01	0.216	2.8E-02	*	0.275	5.1E-03	*
GtsRNA-Asp-GTC	GtsRNA-Asp-GTC-3	Genomic tRNA-Asp-GTC-3	0.098	9.9E-01	0.136	2.3E-01		0.088	4.5E-01	
GtsRNA-Cys-GCA	GtsRNA-Cys-GCA-1	Genomic tRNA-Cys-GCA-1	0.043	9.9E-01	0.087	1.6E-01		0.130	4.2E-03	
GtsRNA-Cys-GCA	GtsRNA-Cys-GCA-11	Genomic tRNA-Cys-GCA-11	0.044	9.6E-01	0.044	2.7E-01		0.084	0.0E+00	
GtsRNA-Cys-GCA	GtsRNA-Cys-GCA-13	Genomic tRNA-Cys-GCA-13	0.050	9.9E-01	0.070	2.6E-01		0.119	4.4E-03	
GtsRNA-Cys-GCA	GtsRNA-Cys-GCA-14	Genomic tRNA-Cys-GCA-14	0.047	9.9E-01	0.072	2.1E-01		0.118	4.4E-03	
GtsRNA-Cys-GCA	GtsRNA-Cys-GCA-15	Genomic tRNA-Cys-GCA-15	0.030	9.9E-01	0.055	2.7E-01		0.084	1.6E-02	
GtsRNA-Cys-GCA	GtsRNA-Cys-GCA-16	Genomic tRNA-Cys-GCA-16	0.023	9.6E-01	0.046	2.3E-02		0.025	1.2E-01	
GtsRNA-Cys-GCA	GtsRNA-Cys-GCA-17	Genomic tRNA-Cys-GCA-17	0.043	9.6E-01	0.044	3.0E-01		0.084	0.0E+00	
GtsRNA-Cys-GCA	GtsRNA-Cys-GCA-18	Genomic tRNA-Cys-GCA-18	0.040	9.6E-01	0.042	2.7E-01		0.077	2.1E-03	

			ES controls *vs.* MS controls			ES controls *vs.* MS patients			MS controls *vs.* MS patients		
sncRNA family	sncRNA subfamily	Parental RNA	*CD*	Adjusted *P*		*CD*	Adjusted *P*		*CD*	Adjusted *P*	
GtsRNA-Cys-GCA	GtsRNA-Cys-GCA-19	Genomic tRNA-Cys-GCA-19	0.026	9.9E-01		0.057	2.5E-01		0.079	2.3E-02	
GtsRNA-Cys-GCA	GtsRNA-Cys-GCA-2	Genomic tRNA-Cys-GCA-2	0.051	9.9E-01		0.105	1.1E-01		0.155	5.4E-03	
GtsRNA-Cys-GCA	GtsRNA-Cys-GCA-20	Genomic tRNA-Cys-GCA-20	0.036	9.6E-01		0.041	2.7E-01		0.071	3.0E-03	
GtsRNA-Cys-GCA	GtsRNA-Cys-GCA-21	Genomic tRNA-Cys-GCA-21	0.045	9.6E-01		0.044	3.1E-01		0.084	2.1E-03	
GtsRNA-Cys-GCA	GtsRNA-Cys-GCA-3	Genomic tRNA-Cys-GCA-3	0.039	9.6E-01		0.049	1.1E-01		0.067	0.0E+00	
GtsRNA-Cys-GCA	GtsRNA-Cys-GCA-4	Genomic tRNA-Cys-GCA-4	0.046	9.9E-01		0.106	1.1E-01		0.151	5.9E-03	
GtsRNA-Cys-GCA	GtsRNA-Cys-GCA-5	Genomic tRNA-Cys-GCA-5	0.054	9.6E-01		0.059	2.2E-01		0.104	3.0E-03	
GtsRNA-Cys-GCA	GtsRNA-Cys-GCA-6	Genomic tRNA-Cys-GCA-6	0.060	9.9E-01		0.179	7.5E-02		0.238	5.4E-03	*
GtsRNA-Cys-GCA	GtsRNA-Cys-GCA-7	Genomic tRNA-Cys-GCA-7	0.051	9.6E-01		0.046	2.7E-01		0.083	0.0E+00	
GtsRNA-Cys-GCA	GtsRNA-Cys-GCA-8	Genomic tRNA-Cys-GCA-8	0.053	9.6E-01		0.078	2.1E-01		0.130	3.0E-03	
GtsRNA-Cys-GCA	GtsRNA-Cys-GCA-9	Genomic tRNA-Cys-GCA-9	0.054	9.6E-01		0.071	2.5E-01		0.125	4.4E-03	
GtsRNA-Gln-CTG	GtsRNA-Gln-CTG-1	Genomic tRNA-Gln-CTG-1	0.040	9.9E-01		0.183	8.0E-02		0.200	2.1E-02	*
GtsRNA-Gln-CTG	GtsRNA-Gln-CTG-2	Genomic tRNA-Gln-CTG-2	0.042	9.9E-01		0.181	8.0E-02		0.196	2.6E-02	
GtsRNA-Gln-CTG	GtsRNA-Gln-CTG-3	Genomic tRNA-Gln-CTG-3	0.051	9.9E-01		0.182	8.0E-02		0.202	2.1E-02	*
GtsRNA-Gln-CTG	GtsRNA-Gln-CTG-4	Genomic tRNA-Gln-CTG-4	0.051	9.9E-01		0.182	8.0E-02		0.202	2.1E-02	*
GtsRNA-Gln-CTG	GtsRNA-Gln-CTG-5	Genomic tRNA-Gln-CTG-5	0.038	9.9E-01		0.187	1.3E-01		0.221	2.0E-02	*
GtsRNA-Gln-CTG	GtsRNA-Gln-CTG-6	Genomic tRNA-Gln-CTG-6	0.028	9.9E-01		0.166	4.9E-02		0.159	4.9E-02	
GtsRNA-Gln-CTG	GtsRNA-Gln-CTG-7	Genomic tRNA-Gln-CTG-7	0.038	9.9E-01		0.175	5.2E-02		0.208	5.4E-03	*
GtsRNA-Gln-TTG	GtsRNA-Gln-TTG-1	Genomic tRNA-Gln-TTG-1	0.040	9.9E-01		0.177	8.0E-02		0.193	2.0E-02	
GtsRNA-Gln-TTG	GtsRNA-Gln-TTG-2	Genomic tRNA-Gln-TTG-2	0.040	9.9E-01		0.190	1.2E-01		0.227	1.6E-02	*
GtsRNA-Gln-TTG	GtsRNA-Gln-TTG-3	Genomic tRNA-Gln-TTG-3	0.041	9.9E-01		0.161	1.0E-01		0.177	2.3E-02	
GtsRNA-Gln-TTG	GtsRNA-Gln-TTG-4	Genomic tRNA-Gln-TTG-4	0.098	9.9E-01		0.064	8.3E-01		0.086	6.0E-01	
GtsRNA-Glu-CTC	GtsRNA-Glu-CTC-1	Genomic tRNA-Glu-CTC-1	0.039	9.9E-01		0.089	8.4E-03		0.066	9.4E-02	
GtsRNA-Glu-CTC	GtsRNA-Glu-CTC-2	Genomic tRNA-Glu-CTC-2	0.037	9.9E-01		0.079	2.3E-02		0.062	1.3E-01	
GtsRNA-Glu-TTC	GtsRNA-Glu-TTC-1	Genomic tRNA-Glu-TTC-1	0.023	9.9E-01		0.078	1.3E-01		0.073	1.5E-01	
GtsRNA-Glu-TTC	GtsRNA-Glu-TTC-2	Genomic tRNA-Glu-TTC-2	0.023	9.9E-01		0.081	1.4E-01		0.071	1.9E-01	
GtsRNA-Glu-TTC	GtsRNA-Glu-TTC-3	Genomic tRNA-Glu-TTC-3	0.030	9.9E-01		0.047	5.1E-01		0.050	4.5E-01	
GtsRNA-Glu-TTC	GtsRNA-Glu-TTC-4	Genomic tRNA-Glu-TTC-4	0.033	9.9E-01		0.038	6.7E-01		0.042	5.9E-01	
GtsRNA-Gly-CCC	GtsRNA-Gly-CCC-1	Genomic tRNA-Gly-CCC-1	0.039	9.9E-01		0.200	8.4E-03		0.169	3.7E-02	
GtsRNA-Gly-CCC	GtsRNA-Gly-CCC-2	Genomic tRNA-Gly-CCC-2	0.016	9.9E-01		0.044	7.5E-03		0.052	2.1E-03	
GtsRNA-Gly-CCC	GtsRNA-Gly-CCC-3	Genomic tRNA-Gly-CCC-3	0.043	9.9E-01		0.233	7.5E-03	*	0.196	1.9E-02	
GtsRNA-Gly-GCC	GtsRNA-Gly-GCC-1	Genomic tRNA-Gly-GCC-1	0.045	9.9E-01		0.216	1.2E-02	*	0.179	4.2E-02	
GtsRNA-Gly-GCC	GtsRNA-Gly-GCC-2	Genomic tRNA-Gly-GCC-2	0.069	9.9E-01		0.246	7.5E-03	*	0.185	5.8E-02	
GtsRNA-Gly-GCC	GtsRNA-Gly-GCC-3	Genomic tRNA-Gly-GCC-3	0.017	9.9E-01		0.153	7.2E-03		0.142	2.0E-02	
GtsRNA-Gly-GCC	GtsRNA-Gly-GCC-4	Genomic tRNA-Gly-GCC-4	0.036	9.9E-01		0.049	4.6E-01		0.061	1.2E-01	

sncRNA family	sncRNA subfamily	Parental RNA	ES controls *vs.* MS controls		ES controls *vs.* MS patients			MS controls *vs.* MS patients		
			CD	Adjusted *P*	*CD*	Adjusted *P*		*CD*	Adjusted *P*	
GtsRNA-Gly-GCC	GtsRNA-Gly-GCC-5	Genomic tRNA-Gly-GCC-5	0.033	9.9E-01	0.206	7.5E-03	*	0.176	2.3E-02	
GtsRNA-Gly-TCC	GtsRNA-Gly-TCC-1	Genomic tRNA-Gly-TCC-1	0.014	9.6E-01	0.031	9.2E-03		0.017	1.1E-01	
GtsRNA-Gly-TCC	GtsRNA-Gly-TCC-2	Genomic tRNA-Gly-TCC-2	0.014	9.6E-01	0.031	8.4E-03		0.017	1.1E-01	
GtsRNA-Gly-TCC	GtsRNA-Gly-TCC-3	Genomic tRNA-Gly-TCC-3	0.014	9.6E-01	0.031	8.4E-03		0.017	1.1E-01	
GtsRNA-Gly-TCC	GtsRNA-Gly-TCC-4	Genomic tRNA-Gly-TCC-4	0.046	9.9E-01	0.033	7.3E-01		0.039	6.2E-01	
GtsRNA-His-GTG	GtsRNA-His-GTG-1	Genomic tRNA-His-GTG-1	0.025	9.9E-01	0.329	0.0E+00	*	0.305	9.8E-03	*
GtsRNA-His-GTG	GtsRNA-His-GTG-2	Genomic tRNA-His-GTG-2	0.046	9.9E-01	0.314	3.3E-03	*	0.270	2.0E-02	*
GtsRNA-iMet-CAT	GtsRNA-iMet-CAT-1	Genomic tRNA-iMet-CAT-1	0.012	9.9E-01	0.077	3.5E-01		0.080	2.0E-01	
GtsRNA-iMet-CAT	GtsRNA-iMet-CAT-2	Genomic tRNA-iMet-CAT-2	0.014	9.9E-01	0.091	2.7E-01		0.094	1.6E-01	
GtsRNA-Leu-AAG	GtsRNA-Leu-AAG-1	Genomic tRNA-Leu-AAG-1	0.013	9.9E-01	0.021	2.1E-01		0.030	2.5E-02	
GtsRNA-Leu-AAG	GtsRNA-Leu-AAG-2	Genomic tRNA-Leu-AAG-2	0.013	9.9E-01	0.020	2.3E-01		0.028	4.1E-02	
GtsRNA-Leu-AAG	GtsRNA-Leu-AAG-3	Genomic tRNA-Leu-AAG-3	0.011	9.9E-01	0.020	2.2E-01		0.027	4.2E-02	
GtsRNA-Leu-AAG	GtsRNA-Leu-AAG-4	Genomic tRNA-Leu-AAG-4	0.006	9.9E-01	0.019	6.2E-02		0.022	2.9E-02	
GtsRNA-Leu-CAA	GtsRNA-Leu-CAA-1	Genomic tRNA-Leu-CAA-1	0.010	9.9E-01	0.105	3.3E-03		0.099	7.8E-03	
GtsRNA-Leu-CAA	GtsRNA-Leu-CAA-2	Genomic tRNA-Leu-CAA-2	0.010	9.9E-01	0.105	3.3E-03		0.100	5.4E-03	
GtsRNA-Leu-CAA	GtsRNA-Leu-CAA-3	Genomic tRNA-Leu-CAA-3	0.010	9.9E-01	0.105	7.5E-03		0.099	1.0E-02	
GtsRNA-Leu-CAA	GtsRNA-Leu-CAA-4	Genomic tRNA-Leu-CAA-4	0.010	9.9E-01	0.105	7.5E-03		0.100	6.0E-03	
GtsRNA-Leu-CAA	GtsRNA-Leu-CAA-6	Genomic tRNA-Leu-CAA-6	0.014	9.9E-01	0.061	2.7E-01		0.058	2.6E-01	
GtsRNA-Leu-CAG	GtsRNA-Leu-CAG-1	Genomic tRNA-Leu-CAG-1	0.012	9.9E-01	0.096	4.9E-02		0.099	2.2E-02	
GtsRNA-Leu-CAG	GtsRNA-Leu-CAG-2	Genomic tRNA-Leu-CAG-2	0.013	9.9E-01	0.101	5.8E-02		0.104	2.1E-02	
GtsRNA-Leu-TAG	GtsRNA-Leu-TAG-1	Genomic tRNA-Leu-TAG-1	0.013	9.9E-01	0.021	2.1E-01		0.031	2.3E-02	
GtsRNA-Leu-TAG	GtsRNA-Leu-TAG-2	Genomic tRNA-Leu-TAG-2	0.011	9.9E-01	0.021	2.0E-01		0.028	5.3E-02	
GtsRNA-Leu-TAG	GtsRNA-Leu-TAG-3	Genomic tRNA-Leu-TAG-3	0.009	9.9E-01	0.018	1.9E-01		0.023	6.3E-02	
GtsRNA-Lys-CTT	GtsRNA-Lys-CTT-1	Genomic tRNA-Lys-CTT-1	0.082	9.9E-01	0.084	5.1E-01		0.164	1.3E-01	
GtsRNA-Lys-CTT	GtsRNA-Lys-CTT-11	Genomic tRNA-Lys-CTT-11	0.025	9.9E-01	0.052	8.0E-02		0.064	1.7E-02	
GtsRNA-Lys-CTT	GtsRNA-Lys-CTT-2	Genomic tRNA-Lys-CTT-2	0.086	9.9E-01	0.077	5.4E-01		0.162	1.5E-01	
GtsRNA-Lys-CTT	GtsRNA-Lys-CTT-3	Genomic tRNA-Lys-CTT-3	0.088	9.9E-01	0.035	8.6E-01		0.121	2.7E-01	
GtsRNA-Lys-CTT	GtsRNA-Lys-CTT-4	Genomic tRNA-Lys-CTT-4	0.080	9.9E-01	0.084	5.1E-01		0.163	1.3E-01	
GtsRNA-Lys-CTT	GtsRNA-Lys-CTT-5	Genomic tRNA-Lys-CTT-5	0.083	9.9E-01	0.060	7.5E-01		0.116	4.1E-01	
GtsRNA-Lys-CTT	GtsRNA-Lys-CTT-7	Genomic tRNA-Lys-CTT-7	0.013	9.9E-01	0.070	2.7E-01		0.069	1.7E-01	
GtsRNA-Lys-CTT	GtsRNA-Lys-CTT-8	Genomic tRNA-Lys-CTT-8	0.014	9.9E-01	0.141	6.0E-02		0.134	3.7E-02	
GtsRNA-Lys-CTT	GtsRNA-Lys-CTT-9	Genomic tRNA-Lys-CTT-9	0.037	9.9E-01	0.050	1.9E-01		0.044	2.9E-01	
GtsRNA-Lys-TTT	GtsRNA-Lys-TTT-2	Genomic tRNA-Lys-TTT-2	0.068	9.9E-01	0.169	1.7E-02		0.139	1.7E-02	
GtsRNA-Lys-TTT	GtsRNA-Lys-TTT-3	Genomic tRNA-Lys-TTT-3	0.049	9.9E-01	0.076	5.1E-01		0.087	2.5E-01	
GtsRNA-Lys-TTT	GtsRNA-Lys-TTT-4	Genomic tRNA-Lys-TTT-4	0.076	9.9E-01	0.062	6.5E-01		0.099	1.9E-01	

			ES controls *vs.* MS controls		ES controls *vs.* MS patients			MS controls *vs.* MS patients		
sncRNA family	sncRNA subfamily	Parental RNA	*CD*	Adjusted *P*	*CD*	Adjusted *P*		*CD*	Adjusted *P*	
GtsRNA-Lys-TTT	GtsRNA-Lys-TTT-5	Genomic tRNA-Lys-TTT-5	0.052	9.9E-01	0.077	5.1E-01		0.096	2.0E-01	
GtsRNA-Lys-TTT	GtsRNA-Lys-TTT-7	Genomic tRNA-Lys-TTT-7	0.012	9.9E-01	0.159	3.3E-03		0.156	2.1E-03	
GtsRNA-Phe-GAA	GtsRNA-Phe-GAA-1	Genomic tRNA-Phe-GAA-1	0.059	9.9E-01	0.039	8.7E-01		0.081	1.8E-01	
GtsRNA-Phe-GAA	GtsRNA-Phe-GAA-2	Genomic tRNA-Phe-GAA-2	0.046	9.9E-01	0.036	8.9E-01		0.050	4.2E-01	
GtsRNA-Phe-GAA	GtsRNA-Phe-GAA-3	Genomic tRNA-Phe-GAA-3	0.047	9.9E-01	0.034	9.0E-01		0.040	5.6E-01	
GtsRNA-Phe-GAA	GtsRNA-Phe-GAA-4	Genomic tRNA-Phe-GAA-4	0.025	9.9E-01	0.046	6.4E-01		0.031	5.5E-01	
GtsRNA-Pro-AGG	GtsRNA-Pro-AGG-1	Genomic tRNA-Pro-AGG-1	0.003	9.9E-01	0.022	8.0E-02		0.021	8.3E-02	
GtsRNA-Pro-AGG	GtsRNA-Pro-AGG-2	Genomic tRNA-Pro-AGG-2	0.004	9.9E-01	0.022	1.1E-01		0.021	1.0E-01	
GtsRNA-Pro-CGG	GtsRNA-Pro-CGG-1	Genomic tRNA-Pro-CGG-1	0.004	9.9E-01	0.022	9.7E-02		0.021	1.1E-01	
GtsRNA-Pro-CGG	GtsRNA-Pro-CGG-2	Genomic tRNA-Pro-CGG-2	0.003	9.9E-01	0.022	8.0E-02		0.021	8.9E-02	
GtsRNA-Pro-TGG	GtsRNA-Pro-TGG-1	Genomic tRNA-Pro-TGG-1	0.004	9.9E-01	0.021	1.0E-01		0.021	1.0E-01	
GtsRNA-Pro-TGG	GtsRNA-Pro-TGG-2	Genomic tRNA-Pro-TGG-2	0.002	1.0E+00	0.022	8.0E-02		0.021	7.7E-02	
GtsRNA-Pro-TGG	GtsRNA-Pro-TGG-3	Genomic tRNA-Pro-TGG-3	0.005	9.9E-01	0.022	1.1E-01		0.022	1.0E-01	
GtsRNA-SeC-TCA	GtsRNA-SeC-TCA-1	Genomic tRNA-SeC-TCA-1	0.148	9.9E-01	0.536	5.6E-03	*	0.406	2.0E-02	*
GtsRNA-Ser-AGA	GtsRNA-Ser-AGA-1	Genomic tRNA-Ser-AGA-1	0.037	9.9E-01	0.184	7.5E-03		0.217	4.4E-03	*
GtsRNA-Ser-AGA	GtsRNA-Ser-AGA-2	Genomic tRNA-Ser-AGA-2	0.045	9.9E-01	0.181	7.5E-03		0.221	5.1E-03	*
GtsRNA-Ser-AGA	GtsRNA-Ser-AGA-3	Genomic tRNA-Ser-AGA-3	0.038	9.9E-01	0.186	7.5E-03		0.217	4.4E-03	*
GtsRNA-Ser-AGA	GtsRNA-Ser-AGA-4	Genomic tRNA-Ser-AGA-4	0.044	9.9E-01	0.180	7.5E-03		0.220	4.4E-03	*
GtsRNA-Ser-GCT	GtsRNA-Ser-GCT-1	Genomic tRNA-Ser-GCT-1	0.004	9.9E-01	0.008	4.9E-01		0.011	2.0E-01	
GtsRNA-Ser-GCT	GtsRNA-Ser-GCT-3	Genomic tRNA-Ser-GCT-3	0.004	9.9E-01	0.011	2.7E-01		0.012	1.1E-01	
GtsRNA-Ser-GCT	GtsRNA-Ser-GCT-4	Genomic tRNA-Ser-GCT-4	0.003	9.9E-01	0.010	2.7E-01		0.012	1.1E-01	
GtsRNA-Ser-GCT	GtsRNA-Ser-GCT-5	Genomic tRNA-Ser-GCT-5	0.003	9.9E-01	0.010	2.7E-01		0.012	1.0E-01	
GtsRNA-Ser-GCT	GtsRNA-Ser-GCT-6	Genomic tRNA-Ser-GCT-6	0.004	9.9E-01	0.010	2.7E-01		0.012	1.1E-01	
GtsRNA-Ser-TGA	GtsRNA-Ser-TGA-1	Genomic tRNA-Ser-TGA-1	0.082	9.9E-01	0.055	6.4E-01		0.126	1.9E-01	
GtsRNA-Ser-TGA	GtsRNA-Ser-TGA-2	Genomic tRNA-Ser-TGA-2	0.037	9.9E-01	0.184	8.4E-03		0.217	4.4E-03	*
GtsRNA-Ser-TGA	GtsRNA-Ser-TGA-3	Genomic tRNA-Ser-TGA-3	0.045	9.9E-01	0.181	7.5E-03		0.221	5.1E-03	*
GtsRNA-Ser-TGA	GtsRNA-Ser-TGA-4	Genomic tRNA-Ser-TGA-4	0.066	9.9E-01	0.163	2.7E-01		0.104	5.1E-01	
GtsRNA-Thr-AGT	GtsRNA-Thr-AGT-1	Genomic tRNA-Thr-AGT-1	0.033	9.6E-01	0.037	2.3E-01		0.065	9.5E-03	
GtsRNA-Thr-AGT	GtsRNA-Thr-AGT-2	Genomic tRNA-Thr-AGT-2	0.035	9.6E-01	0.031	4.8E-01		0.059	2.1E-02	
GtsRNA-Thr-AGT	GtsRNA-Thr-AGT-5	Genomic tRNA-Thr-AGT-5	0.035	9.6E-01	0.038	2.7E-01		0.065	9.8E-03	
GtsRNA-Thr-AGT	GtsRNA-Thr-AGT-6	Genomic tRNA-Thr-AGT-6	0.031	9.6E-01	0.029	3.8E-01		0.059	1.9E-02	
GtsRNA-Thr-CGT	GtsRNA-Thr-CGT-3	Genomic tRNA-Thr-CGT-3	0.033	9.6E-01	0.029	4.2E-01		0.059	1.6E-02	
GtsRNA-Trp-CCA	GtsRNA-Trp-CCA-1	Genomic tRNA-Trp-CCA-1	0.037	9.9E-01	0.034	5.4E-01		0.058	4.2E-02	
GtsRNA-Trp-CCA	GtsRNA-Trp-CCA-2	Genomic tRNA-Trp-CCA-2	0.034	9.9E-01	0.021	9.0E-01		0.045	1.6E-01	
GtsRNA-Trp-CCA	GtsRNA-Trp-CCA-3	Genomic tRNA-Trp-CCA-3	0.037	9.9E-01	0.032	6.5E-01		0.055	5.8E-02	

			ES controls *vs.* MS controls		ES controls *vs.* MS patients			MS controls *vs.* MS patients		
sncRNA family	sncRNA subfamily	Parental RNA	*CD*	Adjusted *P*	*CD*	Adjusted *P*		*CD*	Adjusted *P*	
GtsRNA-Trp-CCA	GtsRNA-Trp-CCA-4	Genomic tRNA-Trp-CCA-4	0.038	9.9E-01	0.018	9.6E-01		0.046	1.3E-01	
GtsRNA-Trp-CCA	GtsRNA-Trp-CCA-5	Genomic tRNA-Trp-CCA-5	0.035	9.9E-01	0.024	8.2E-01		0.042	1.6E-01	
GtsRNA-Tyr-GTA	GtsRNA-Tyr-GTA-1	Genomic tRNA-Tyr-GTA-1	0.096	9.6E-01	0.087	2.1E-01		0.057	5.5E-01	
GtsRNA-Tyr-GTA	GtsRNA-Tyr-GTA-2	Genomic tRNA-Tyr-GTA-2	0.096	9.6E-01	0.072	2.5E-01		0.066	3.8E-01	
GtsRNA-Tyr-GTA	GtsRNA-Tyr-GTA-5	Genomic tRNA-Tyr-GTA-5	0.073	9.6E-01	0.064	2.7E-01		0.055	4.9E-01	
GtsRNA-Tyr-GTA	GtsRNA-Tyr-GTA-8	Genomic tRNA-Tyr-GTA-8	0.078	9.6E-01	0.088	4.9E-02		0.063	3.3E-01	
GtsRNA-Val-AAC	GtsRNA-Val-AAC-1	Genomic tRNA-Val-AAC-1	0.025	9.9E-01	0.013	9.0E-01		0.025	7.6E-01	
GtsRNA-Val-AAC	GtsRNA-Val-AAC-2	Genomic tRNA-Val-AAC-2	0.025	9.9E-01	0.015	8.9E-01		0.034	6.4E-01	
GtsRNA-Val-AAC	GtsRNA-Val-AAC-3	Genomic tRNA-Val-AAC-3	0.028	9.9E-01	0.010	9.2E-01		0.038	7.0E-01	
GtsRNA-Val-AAC	GtsRNA-Val-AAC-4	Genomic tRNA-Val-AAC-4	0.025	9.9E-01	0.014	9.0E-01		0.026	7.6E-01	
GtsRNA-Val-AAC	GtsRNA-Val-AAC-5	Genomic tRNA-Val-AAC-5	0.023	9.9E-01	0.017	8.3E-01		0.034	6.2E-01	
GtsRNA-Val-CAC	GtsRNA-Val-CAC-1	Genomic tRNA-Val-CAC-1	0.024	9.9E-01	0.013	9.0E-01		0.025	7.6E-01	
GtsRNA-Val-CAC	GtsRNA-Val-CAC-2	Genomic tRNA-Val-CAC-2	0.083	9.9E-01	0.262	8.8E-02	*	0.340	2.1E-02	*
GtsRNA-Val-CAC	GtsRNA-Val-CAC-3	Genomic tRNA-Val-CAC-3	0.005	9.9E-01	0.015	5.4E-01		0.013	7.6E-01	
GtsRNA-Val-CAC	GtsRNA-Val-CAC-4	Genomic tRNA-Val-CAC-4	0.025	9.9E-01	0.017	8.9E-01		0.038	6.9E-01	
GtsRNA-Val-CAC	GtsRNA-Val-CAC-5	Genomic tRNA-Val-CAC-5	0.024	9.9E-01	0.012	9.1E-01		0.026	7.6E-01	
GtsRNA-Val-CAC	GtsRNA-Val-CAC-6	Genomic tRNA-Val-CAC-6	0.027	9.9E-01	0.007	9.6E-01		0.029	7.3E-01	
GtsRNA-Val-TAC	GtsRNA-Val-TAC-1	Genomic tRNA-Val-TAC-1	0.029	9.9E-01	0.062	2.7E-01		0.062	2.5E-01	
GtsRNA-Val-TAC	GtsRNA-Val-TAC-2	Genomic tRNA-Val-TAC-2	0.030	9.9E-01	0.071	2.0E-01		0.077	1.1E-01	
GtsRNA-Val-TAC	GtsRNA-Val-TAC-3	Genomic tRNA-Val-TAC-3	0.024	9.9E-01	0.088	1.2E-01		0.073	2.5E-01	
GtsRNA-Val-TAC	GtsRNA-Val-TAC-4	Genomic tRNA-Val-TAC-4	0.014	9.9E-01	0.015	6.4E-01		0.014	6.4E-01	
MtsRNA-Met-CAT	MtsRNA-Met-CAT-1	Mitochondrial tRNA-Met-CAT-1	0.034	9.9E-01	0.116	8.0E-02		0.142	5.9E-03	
MtsRNA-Val-TAC	MtsRNA-Val-TAC-1	Mitochondrial tRNA-Val-TAC-1	0.059	9.9E-01	0.041	8.7E-01		0.087	4.3E-01	
rsRNA-5S	rsRNA-5S	5S rRNA	0.033	9.9E-01	0.125	1.7E-01		0.105	1.2E-01	
rsRNA-5.8S	rsRNA-5.8S	5.8S rRNA	0.092	9.9E-01	0.115	5.6E-01		0.052	7.5E-01	
rsRNA-16S	rsRNA-16S	16S rRNA	0.112	9.9E-01	0.173	4.5E-01		0.202	1.9E-01	
rsRNA-18S	rsRNA-18S	18S rRNA	0.218	9.6E-01	0.390	1.8E-02	*	0.274	4.4E-02	*
rsRNA-28S	rsRNA-28S	28S rRNA	0.101	9.9E-01	0.246	1.0E-01		0.288	1.4E-02	*
rsRNA-45S	rsRNA-45S	45S rRNA	0.106	9.9E-01	0.211	1.5E-01		0.299	1.6E-02	*

Note - Differential fragmentation analysis was performed using *qMAP* Model 2. *: differentially fragmented parental RNAs; *CD*: coverage difference.
