# Supplementary material for: qMAP decodes RNA fragmentation dynamics in development and disease
Source: Mol Syst Biol. 2026 Jul 20;22(10):1626–49. doi: 10.1038/s44320-026-00237-2 (PMC13638987; doi:10.1038/s44320-026-00237-2)
Supplement: Supplementary file 5 [file 44320_2026_237_MOESM4_ESM.pdf]

Table EV4. RNA fragmentation signature of RIF

	ES controls *vs.* MS patients		MS controls *vs.* MS patients	
Parental RNA	Coverage difference	Adjusted *P*	Coverage difference	Adjusted *P*
Genomic tRNA-Asp-GTC-1	0.218	2.8E-02	0.268	5.1E-03
Genomic tRNA-Asp-GTC-2	0.216	2.8E-02	0.275	5.1E-03
Genomic tRNA-His-GTG-1	0.329	0.0E+00	0.305	9.8E-03
Genomic tRNA-His-GTG-2	0.314	3.3E-03	0.270	2.0E-02
Genomic tRNA-SeC-TCA-1	0.536	5.6E-03	0.406	2.0E-02
Genomic tRNA-Val-CAC-2	0.262	8.8E-02	0.340	2.1E-02
18S rRNA	0.390	1.8E-02	0.274	4.4E-02

Note - Differential fragmentation analysis was performed using *qMAP* Model 2.
