# Supplementary material for: qMAP decodes RNA fragmentation dynamics in development and disease
Source: Mol Syst Biol. 2026 Jul 20;22(10):1626–49. doi: 10.1038/s44320-026-00237-2 (PMC13638987; doi:10.1038/s44320-026-00237-2)
Supplement: Supplementary file 6 [file 44320_2026_237_MOESM5_ESM.pdf]

Table EV5. Differential fragmentation analysis between sperm heads from young and old mice

sncRNA family	sncRNA subfamily	Parental RNA	*CD*	Adjusted *P*	
GtsRNA-Ala-AGC	GtsRNA-Ala-AGC-1	Genomic tRNA-Ala-AGC-1	0.227	4.0E-81	*
GtsRNA-Ala-AGC	GtsRNA-Ala-AGC-10	Genomic tRNA-Ala-AGC-10	0.171	2.1E-01	
GtsRNA-Ala-AGC	GtsRNA-Ala-AGC-2	Genomic tRNA-Ala-AGC-2	0.387	4.2E-96	*
GtsRNA-Ala-AGC	GtsRNA-Ala-AGC-3	Genomic tRNA-Ala-AGC-3	1.060	7.4E-163	*
GtsRNA-Ala-AGC	GtsRNA-Ala-AGC-4	Genomic tRNA-Ala-AGC-4	0.274	9.0E-13	*
GtsRNA-Ala-AGC	GtsRNA-Ala-AGC-5	Genomic tRNA-Ala-AGC-5	0.258	8.3E-07	*
GtsRNA-Ala-AGC	GtsRNA-Ala-AGC-6	Genomic tRNA-Ala-AGC-6	0.193	1.7E-03	
GtsRNA-Ala-AGC	GtsRNA-Ala-AGC-7	Genomic tRNA-Ala-AGC-7	0.228	8.0E-02	*
GtsRNA-Ala-AGC	GtsRNA-Ala-AGC-8	Genomic tRNA-Ala-AGC-8	0.030	7.3E-02	
GtsRNA-Ala-CGC	GtsRNA-Ala-CGC-1	Genomic tRNA-Ala-CGC-1	1.047	7.5E-168	*
GtsRNA-Ala-CGC	GtsRNA-Ala-CGC-2	Genomic tRNA-Ala-CGC-2	1.033	1.8E-158	*
GtsRNA-Ala-CGC	GtsRNA-Ala-CGC-3	Genomic tRNA-Ala-CGC-3	0.281	1.6E-69	*
GtsRNA-Ala-CGC	GtsRNA-Ala-CGC-4	Genomic tRNA-Ala-CGC-4	0.501	3.8E-88	*
GtsRNA-Ala-CGC	GtsRNA-Ala-CGC-5	Genomic tRNA-Ala-CGC-5	0.019	1.0E+00	
GtsRNA-Ala-CGC	GtsRNA-Ala-CGC-6	Genomic tRNA-Ala-CGC-6	0.270	1.2E-58	*
GtsRNA-Ala-CGC	GtsRNA-Ala-CGC-7	Genomic tRNA-Ala-CGC-7	1.110	8.5E-160	*
GtsRNA-Ala-TGC	GtsRNA-Ala-TGC-1	Genomic tRNA-Ala-TGC-1	1.010	1.1E-162	*
GtsRNA-Ala-TGC	GtsRNA-Ala-TGC-2	Genomic tRNA-Ala-TGC-2	1.043	9.3E-162	*
GtsRNA-Ala-TGC	GtsRNA-Ala-TGC-4	Genomic tRNA-Ala-TGC-4	1.018	3.7E-151	*
GtsRNA-Ala-TGC	GtsRNA-Ala-TGC-5	Genomic tRNA-Ala-TGC-5	0.948	1.8E-172	*
GtsRNA-Ala-TGC	GtsRNA-Ala-TGC-6	Genomic tRNA-Ala-TGC-6	1.044	3.0E-172	*
GtsRNA-Ala-TGC	GtsRNA-Ala-TGC-7	Genomic tRNA-Ala-TGC-7	0.946	4.3E-170	*
GtsRNA-Ala-TGC	GtsRNA-Ala-TGC-8	Genomic tRNA-Ala-TGC-8	0.315	6.0E-123	*
GtsRNA-Arg-ACG	GtsRNA-Arg-ACG-1	Genomic tRNA-Arg-ACG-1	0.021	1.0E+00	
GtsRNA-Arg-ACG	GtsRNA-Arg-ACG-2	Genomic tRNA-Arg-ACG-2	0.130	2.2E-02	
GtsRNA-Arg-ACG	GtsRNA-Arg-ACG-3	Genomic tRNA-Arg-ACG-3	0.023	1.0E+00	
GtsRNA-Arg-CCG	GtsRNA-Arg-CCG-1	Genomic tRNA-Arg-CCG-1	0.033	8.7E-02	
GtsRNA-Arg-CCG	GtsRNA-Arg-CCG-2	Genomic tRNA-Arg-CCG-2	0.026	9.8E-01	
GtsRNA-Arg-CCG	GtsRNA-Arg-CCG-3	Genomic tRNA-Arg-CCG-3	0.200	4.3E-107	*
GtsRNA-Arg-CCT	GtsRNA-Arg-CCT-1	Genomic tRNA-Arg-CCT-1	0.128	2.1E-64	
GtsRNA-Arg-CCT	GtsRNA-Arg-CCT-2	Genomic tRNA-Arg-CCT-2	0.340	3.5E-114	*
GtsRNA-Arg-CCT	GtsRNA-Arg-CCT-3	Genomic tRNA-Arg-CCT-3	0.045	8.9E-24	
GtsRNA-Arg-CCT	GtsRNA-Arg-CCT-4	Genomic tRNA-Arg-CCT-4	0.295	9.3E-67	*
GtsRNA-Arg-TCG	GtsRNA-Arg-TCG-1	Genomic tRNA-Arg-TCG-1	0.014	1.0E+00	

sncRNA family	sncRNA subfamily	Parental RNA	*CD*	Adjusted *P*	
GtsRNA-Arg-TCG	GtsRNA-Arg-TCG-2	Genomic tRNA-Arg-TCG-2	0.047	2.1E-11	
GtsRNA-Arg-TCG	GtsRNA-Arg-TCG-3	Genomic tRNA-Arg-TCG-3	0.137	4.0E-16	
GtsRNA-Arg-TCG	GtsRNA-Arg-TCG-4	Genomic tRNA-Arg-TCG-4	0.138	1.3E-13	
GtsRNA-Arg-TCT	GtsRNA-Arg-TCT-1	Genomic tRNA-Arg-TCT-1	0.448	6.2E-130	*
GtsRNA-Arg-TCT	GtsRNA-Arg-TCT-2	Genomic tRNA-Arg-TCT-2	0.110	5.4E-41	
GtsRNA-Arg-TCT	GtsRNA-Arg-TCT-3	Genomic tRNA-Arg-TCT-3	0.112	1.0E-23	
GtsRNA-Arg-TCT	GtsRNA-Arg-TCT-4	Genomic tRNA-Arg-TCT-4	0.308	2.8E-01	
GtsRNA-Arg-TCT	GtsRNA-Arg-TCT-5	Genomic tRNA-Arg-TCT-5	0.048	1.0E+00	
GtsRNA-Asn-GTT	GtsRNA-Asn-GTT-1	Genomic tRNA-Asn-GTT-1	0.062	2.1E-01	
GtsRNA-Asn-GTT	GtsRNA-Asn-GTT-2	Genomic tRNA-Asn-GTT-2	0.063	2.5E-01	
GtsRNA-Asn-GTT	GtsRNA-Asn-GTT-3	Genomic tRNA-Asn-GTT-3	0.062	2.6E-01	
GtsRNA-Asn-GTT	GtsRNA-Asn-GTT-4	Genomic tRNA-Asn-GTT-4	0.471	2.9E-21	*
GtsRNA-Asn-GTT	GtsRNA-Asn-GTT-5	Genomic tRNA-Asn-GTT-5	0.716	1.5E-04	*
GtsRNA-Asp-GTC	GtsRNA-Asp-GTC-1	Genomic tRNA-Asp-GTC-1	0.221	5.0E-137	*
GtsRNA-Asp-GTC	GtsRNA-Asp-GTC-2	Genomic tRNA-Asp-GTC-2	0.231	1.5E-149	*
GtsRNA-Asp-GTC	GtsRNA-Asp-GTC-3	Genomic tRNA-Asp-GTC-3	0.015	1.0E+00	
GtsRNA-Asp-GTC	GtsRNA-Asp-GTC-4	Genomic tRNA-Asp-GTC-4	0.146	8.8E-69	
GtsRNA-Cys-GCA	GtsRNA-Cys-GCA-1	Genomic tRNA-Cys-GCA-1	0.126	2.2E-54	
GtsRNA-Cys-GCA	GtsRNA-Cys-GCA-10	Genomic tRNA-Cys-GCA-10	0.097	1.3E-68	
GtsRNA-Cys-GCA	GtsRNA-Cys-GCA-11	Genomic tRNA-Cys-GCA-11	0.425	4.6E-12	*
GtsRNA-Cys-GCA	GtsRNA-Cys-GCA-12	Genomic tRNA-Cys-GCA-12	0.086	1.1E-47	
GtsRNA-Cys-GCA	GtsRNA-Cys-GCA-13	Genomic tRNA-Cys-GCA-13	0.097	5.8E-10	
GtsRNA-Cys-GCA	GtsRNA-Cys-GCA-14	Genomic tRNA-Cys-GCA-14	0.112	8.2E-40	
GtsRNA-Cys-GCA	GtsRNA-Cys-GCA-15	Genomic tRNA-Cys-GCA-15	0.084	1.7E-28	
GtsRNA-Cys-GCA	GtsRNA-Cys-GCA-16	Genomic tRNA-Cys-GCA-16	0.187	8.1E-60	
GtsRNA-Cys-GCA	GtsRNA-Cys-GCA-17	Genomic tRNA-Cys-GCA-17	0.145	6.1E-49	
GtsRNA-Cys-GCA	GtsRNA-Cys-GCA-18	Genomic tRNA-Cys-GCA-18	0.514	2.9E-35	*
GtsRNA-Cys-GCA	GtsRNA-Cys-GCA-19	Genomic tRNA-Cys-GCA-19	0.086	1.3E-37	
GtsRNA-Cys-GCA	GtsRNA-Cys-GCA-2	Genomic tRNA-Cys-GCA-2	0.153	2.9E-71	
GtsRNA-Cys-GCA	GtsRNA-Cys-GCA-21	Genomic tRNA-Cys-GCA-21	0.295	1.9E-22	*
GtsRNA-Cys-GCA	GtsRNA-Cys-GCA-24	Genomic tRNA-Cys-GCA-24	0.066	2.0E-43	
GtsRNA-Cys-GCA	GtsRNA-Cys-GCA-25	Genomic tRNA-Cys-GCA-25	0.059	4.1E-01	
GtsRNA-Cys-GCA	GtsRNA-Cys-GCA-3	Genomic tRNA-Cys-GCA-3	0.155	9.8E-73	
GtsRNA-Cys-GCA	GtsRNA-Cys-GCA-4	Genomic tRNA-Cys-GCA-4	0.133	8.2E-56	
GtsRNA-Cys-GCA	GtsRNA-Cys-GCA-5	Genomic tRNA-Cys-GCA-5	0.162	3.8E-74	
GtsRNA-Cys-GCA	GtsRNA-Cys-GCA-6	Genomic tRNA-Cys-GCA-6	0.099	6.1E-71	

sncRNA family	sncRNA subfamily	Parental RNA	*CD*	Adjusted *P*	
GtsRNA-Cys-GCA	GtsRNA-Cys-GCA-7	Genomic tRNA-Cys-GCA-7	0.163	1.7E-75	
GtsRNA-Cys-GCA	GtsRNA-Cys-GCA-8	Genomic tRNA-Cys-GCA-8	0.180	3.0E-66	
GtsRNA-Cys-GCA	GtsRNA-Cys-GCA-9	Genomic tRNA-Cys-GCA-9	0.092	2.8E-27	
GtsRNA-Gln-CTG	GtsRNA-Gln-CTG-1	Genomic tRNA-Gln-CTG-1	0.580	2.0E-152	*
GtsRNA-Gln-CTG	GtsRNA-Gln-CTG-2	Genomic tRNA-Gln-CTG-2	0.509	5.3E-144	*
GtsRNA-Gln-CTG	GtsRNA-Gln-CTG-3	Genomic tRNA-Gln-CTG-3	0.508	2.4E-143	*
GtsRNA-Gln-CTG	GtsRNA-Gln-CTG-4	Genomic tRNA-Gln-CTG-4	0.543	1.1E-141	*
GtsRNA-Gln-CTG	GtsRNA-Gln-CTG-5	Genomic tRNA-Gln-CTG-5	0.037	2.1E-06	
GtsRNA-Gln-TTG	GtsRNA-Gln-TTG-1	Genomic tRNA-Gln-TTG-1	0.568	7.3E-137	*
GtsRNA-Gln-TTG	GtsRNA-Gln-TTG-2	Genomic tRNA-Gln-TTG-2	0.573	8.3E-182	*
GtsRNA-Gln-TTG	GtsRNA-Gln-TTG-3	Genomic tRNA-Gln-TTG-3	0.569	1.7E-118	*
GtsRNA-Gln-TTG	GtsRNA-Gln-TTG-4	Genomic tRNA-Gln-TTG-4	0.030	1.0E+00	
GtsRNA-Gln-TTG	GtsRNA-Gln-TTG-5	Genomic tRNA-Gln-TTG-5	0.579	3.1E-169	*
GtsRNA-Glu-CTC	GtsRNA-Glu-CTC-1	Genomic tRNA-Glu-CTC-1	0.012	1.0E-17	
GtsRNA-Glu-CTC	GtsRNA-Glu-CTC-2	Genomic tRNA-Glu-CTC-2	0.012	2.0E-19	
GtsRNA-Glu-CTC	GtsRNA-Glu-CTC-5	Genomic tRNA-Glu-CTC-5	0.007	8.2E-01	
GtsRNA-Glu-TTC	GtsRNA-Glu-TTC-1	Genomic tRNA-Glu-TTC-1	0.350	2.6E-66	*
GtsRNA-Glu-TTC	GtsRNA-Glu-TTC-2	Genomic tRNA-Glu-TTC-2	0.362	6.5E-68	*
GtsRNA-Glu-TTC	GtsRNA-Glu-TTC-3	Genomic tRNA-Glu-TTC-3	0.051	8.0E-37	
GtsRNA-Gly-ACC	GtsRNA-Gly-ACC-1	Genomic tRNA-Gly-ACC-1	0.225	3.8E-15	*
GtsRNA-Gly-CCC	GtsRNA-Gly-CCC-1	Genomic tRNA-Gly-CCC-1	0.422	3.0E-45	*
GtsRNA-Gly-CCC	GtsRNA-Gly-CCC-2	Genomic tRNA-Gly-CCC-2	0.379	5.8E-33	*
GtsRNA-Gly-CCC	GtsRNA-Gly-CCC-3	Genomic tRNA-Gly-CCC-3	0.008	2.0E-01	
GtsRNA-Gly-CCC	GtsRNA-Gly-CCC-4	Genomic tRNA-Gly-CCC-4	0.008	2.4E-01	
GtsRNA-Gly-GCC	GtsRNA-Gly-GCC-1	Genomic tRNA-Gly-GCC-1	0.015	1.0E+00	
GtsRNA-Gly-GCC	GtsRNA-Gly-GCC-2	Genomic tRNA-Gly-GCC-2	0.014	1.0E+00	
GtsRNA-Gly-GCC	GtsRNA-Gly-GCC-3	Genomic tRNA-Gly-GCC-3	0.013	1.0E+00	
GtsRNA-Gly-GCC	GtsRNA-Gly-GCC-4	Genomic tRNA-Gly-GCC-4	0.013	1.0E+00	
GtsRNA-Gly-TCC	GtsRNA-Gly-TCC-1	Genomic tRNA-Gly-TCC-1	0.220	9.5E-07	*
GtsRNA-His-GTG	GtsRNA-His-GTG-1	Genomic tRNA-His-GTG-1	0.084	4.6E-06	
GtsRNA-His-GTG	GtsRNA-His-GTG-2	Genomic tRNA-His-GTG-2	0.046	5.1E-31	
GtsRNA-His-GTG	GtsRNA-His-GTG-3	Genomic tRNA-His-GTG-3	0.051	2.3E-31	
GtsRNA-Ile-AAT	GtsRNA-Ile-AAT-1	Genomic tRNA-Ile-AAT-1	0.060	1.0E+00	
GtsRNA-Ile-AAT	GtsRNA-Ile-AAT-2	Genomic tRNA-Ile-AAT-2	0.438	4.9E-10	*
GtsRNA-Ile-AAT	GtsRNA-Ile-AAT-3	Genomic tRNA-Ile-AAT-3	0.057	1.0E+00	
GtsRNA-Ile-AAT	GtsRNA-Ile-AAT-4	Genomic tRNA-Ile-AAT-4	0.826	1.8E-06	*

sncRNA family	sncRNA subfamily	Parental RNA	*CD*	Adjusted *P*	
GtsRNA-Ile-TAT	GtsRNA-Ile-TAT-1	Genomic tRNA-Ile-TAT-1	0.156	8.9E-01	
GtsRNA-Ile-TAT	GtsRNA-Ile-TAT-2	Genomic tRNA-Ile-TAT-2	0.139	1.0E+00	
GtsRNA-Ile-TAT	GtsRNA-Ile-TAT-3	Genomic tRNA-Ile-TAT-3	0.423	2.2E-07	*
GtsRNA-iMet-CA	GtsRNA-iMet-CAT-1	Genomic tRNA-iMet-CAT-1	0.204	2.8E-88	*
GtsRNA-iMet-CA	GtsRNA-iMet-CAT-2	Genomic tRNA-iMet-CAT-2	0.213	1.7E-88	*
GtsRNA-iMet-CA	GtsRNA-iMet-CAT-3	Genomic tRNA-iMet-CAT-3	0.211	8.9E-78	*
GtsRNA-Leu-AAG	GtsRNA-Leu-AAG-1	Genomic tRNA-Leu-AAG-1	0.159	3.7E-36	
GtsRNA-Leu-AAG	GtsRNA-Leu-AAG-2	Genomic tRNA-Leu-AAG-2	0.157	1.2E-35	
GtsRNA-Leu-AAG	GtsRNA-Leu-AAG-3	Genomic tRNA-Leu-AAG-3	0.162	1.7E-36	
GtsRNA-Leu-CAA	GtsRNA-Leu-CAA-1	Genomic tRNA-Leu-CAA-1	0.265	6.0E-166	*
GtsRNA-Leu-CAA	GtsRNA-Leu-CAA-2	Genomic tRNA-Leu-CAA-2	0.397	6.7E-159	*
GtsRNA-Leu-CAA	GtsRNA-Leu-CAA-3	Genomic tRNA-Leu-CAA-3	0.400	5.8E-157	*
GtsRNA-Leu-CAA	GtsRNA-Leu-CAA-4	Genomic tRNA-Leu-CAA-4	0.442	2.0E-161	*
GtsRNA-Leu-CAG	GtsRNA-Leu-CAG-1	Genomic tRNA-Leu-CAG-1	0.327	4.4E-133	*
GtsRNA-Leu-CAG	GtsRNA-Leu-CAG-2	Genomic tRNA-Leu-CAG-2	0.263	1.1E-128	*
GtsRNA-Leu-CAG	GtsRNA-Leu-CAG-3	Genomic tRNA-Leu-CAG-3	0.319	5.9E-125	*
GtsRNA-Leu-CAG	GtsRNA-Leu-CAG-4	Genomic tRNA-Leu-CAG-4	0.400	2.2E-65	*
GtsRNA-Leu-TAA	GtsRNA-Leu-TAA-1	Genomic tRNA-Leu-TAA-1	0.184	1.0E+00	
GtsRNA-Leu-TAA	GtsRNA-Leu-TAA-2	Genomic tRNA-Leu-TAA-2	0.164	1.2E-38	
GtsRNA-Leu-TAA	GtsRNA-Leu-TAA-3	Genomic tRNA-Leu-TAA-3	0.458	5.3E-05	*
GtsRNA-Leu-TAA	GtsRNA-Leu-TAA-4	Genomic tRNA-Leu-TAA-4	0.131	8.2E-01	
GtsRNA-Leu-TAG	GtsRNA-Leu-TAG-1	Genomic tRNA-Leu-TAG-1	0.170	9.6E-50	
GtsRNA-Leu-TAG	GtsRNA-Leu-TAG-2	Genomic tRNA-Leu-TAG-2	0.144	2.2E-39	
GtsRNA-Leu-TAG	GtsRNA-Leu-TAG-3	Genomic tRNA-Leu-TAG-3	0.347	2.2E-15	*
GtsRNA-Lys-CTT	GtsRNA-Lys-CTT-1	Genomic tRNA-Lys-CTT-1	0.013	1.0E+00	
GtsRNA-Lys-CTT	GtsRNA-Lys-CTT-12	Genomic tRNA-Lys-CTT-12	0.106	7.2E-01	
GtsRNA-Lys-CTT	GtsRNA-Lys-CTT-13	Genomic tRNA-Lys-CTT-13	0.086	7.4E-21	
GtsRNA-Lys-CTT	GtsRNA-Lys-CTT-14	Genomic tRNA-Lys-CTT-14	0.013	1.0E+00	
GtsRNA-Lys-CTT	GtsRNA-Lys-CTT-2	Genomic tRNA-Lys-CTT-2	0.219	1.8E-23	*
GtsRNA-Lys-CTT	GtsRNA-Lys-CTT-3	Genomic tRNA-Lys-CTT-3	0.244	3.1E-43	*
GtsRNA-Lys-CTT	GtsRNA-Lys-CTT-4	Genomic tRNA-Lys-CTT-4	0.087	5.2E-06	
GtsRNA-Lys-CTT	GtsRNA-Lys-CTT-5	Genomic tRNA-Lys-CTT-5	0.064	2.0E-01	
GtsRNA-Lys-CTT	GtsRNA-Lys-CTT-6	Genomic tRNA-Lys-CTT-6	0.041	1.5E-04	
GtsRNA-Lys-CTT	GtsRNA-Lys-CTT-7	Genomic tRNA-Lys-CTT-7	0.043	2.1E-03	
GtsRNA-Lys-CTT	GtsRNA-Lys-CTT-8	Genomic tRNA-Lys-CTT-8	0.120	1.7E-25	
GtsRNA-Lys-CTT	GtsRNA-Lys-CTT-9	Genomic tRNA-Lys-CTT-9	0.125	4.6E-07	

sncRNA family	sncRNA subfamily	Parental RNA	*CD*	Adjusted *P*	
GtsRNA-Lys-TTT	GtsRNA-Lys-TTT-1	Genomic tRNA-Lys-TTT-1	0.068	1.8E-38	
GtsRNA-Lys-TTT	GtsRNA-Lys-TTT-2	Genomic tRNA-Lys-TTT-2	0.060	4.1E-12	
GtsRNA-Lys-TTT	GtsRNA-Lys-TTT-3	Genomic tRNA-Lys-TTT-3	0.030	2.9E-25	
GtsRNA-Lys-TTT	GtsRNA-Lys-TTT-4	Genomic tRNA-Lys-TTT-4	0.142	1.0E+00	
GtsRNA-Lys-TTT	GtsRNA-Lys-TTT-5	Genomic tRNA-Lys-TTT-5	0.269	2.9E-17	*
GtsRNA-Met-CAT	GtsRNA-Met-CAT-1	Genomic tRNA-Met-CAT-1	0.010	1.0E+00	
GtsRNA-Met-CAT	GtsRNA-Met-CAT-2	Genomic tRNA-Met-CAT-2	0.020	7.4E-05	
GtsRNA-Met-CAT	GtsRNA-Met-CAT-3	Genomic tRNA-Met-CAT-3	0.020	8.9E-04	
GtsRNA-Met-CAT	GtsRNA-Met-CAT-4	Genomic tRNA-Met-CAT-4	0.068	1.6E-03	
GtsRNA-Met-CAT	GtsRNA-Met-CAT-5	Genomic tRNA-Met-CAT-5	0.021	1.7E-02	
GtsRNA-Met-CAT	GtsRNA-Met-CAT-6	Genomic tRNA-Met-CAT-6	0.054	7.1E-01	
GtsRNA-Phe-GAA	GtsRNA-Phe-GAA-1	Genomic tRNA-Phe-GAA-1	0.196	5.1E-06	
GtsRNA-Phe-GAA	GtsRNA-Phe-GAA-2	Genomic tRNA-Phe-GAA-2	0.211	1.7E-05	*
GtsRNA-Phe-GAA	GtsRNA-Phe-GAA-3	Genomic tRNA-Phe-GAA-3	0.203	4.5E-05	*
GtsRNA-Pro-AGG	GtsRNA-Pro-AGG-1	Genomic tRNA-Pro-AGG-1	0.122	7.4E-05	
GtsRNA-Pro-CGG	GtsRNA-Pro-CGG-1	Genomic tRNA-Pro-CGG-1	0.121	7.4E-05	
GtsRNA-Pro-TGG	GtsRNA-Pro-TGG-1	Genomic tRNA-Pro-TGG-1	0.115	4.1E-13	
GtsRNA-Pro-TGG	GtsRNA-Pro-TGG-2	Genomic tRNA-Pro-TGG-2	0.121	1.4E-04	
GtsRNA-Pro-TGG	GtsRNA-Pro-TGG-3	Genomic tRNA-Pro-TGG-3	0.066	6.4E-33	
GtsRNA-Pro-TGG	GtsRNA-Pro-TGG-4	Genomic tRNA-Pro-TGG-4	0.019	1.9E-32	
GtsRNA-SeC-TCA	GtsRNA-SeC-TCA-1	Genomic tRNA-SeC-TCA-1	0.179	1.4E-17	
GtsRNA-Ser-AGA	GtsRNA-Ser-AGA-1	Genomic tRNA-Ser-AGA-1	0.318	4.3E-60	*
GtsRNA-Ser-AGA	GtsRNA-Ser-AGA-2	Genomic tRNA-Ser-AGA-2	0.336	6.5E-64	*
GtsRNA-Ser-AGA	GtsRNA-Ser-AGA-3	Genomic tRNA-Ser-AGA-3	0.012	1.0E+00	
GtsRNA-Ser-CGA	GtsRNA-Ser-CGA-1	Genomic tRNA-Ser-CGA-1	0.457	2.0E-64	*
GtsRNA-Ser-CGA	GtsRNA-Ser-CGA-2	Genomic tRNA-Ser-CGA-2	0.597	4.0E-10	*
GtsRNA-Ser-CGA	GtsRNA-Ser-CGA-3	Genomic tRNA-Ser-CGA-3	0.180	5.1E-01	
GtsRNA-Ser-GCT	GtsRNA-Ser-GCT-1	Genomic tRNA-Ser-GCT-1	0.342	7.2E-89	*
GtsRNA-Ser-GCT	GtsRNA-Ser-GCT-2	Genomic tRNA-Ser-GCT-2	0.309	6.6E-107	*
GtsRNA-Ser-GCT	GtsRNA-Ser-GCT-3	Genomic tRNA-Ser-GCT-3	0.181	5.6E-54	
GtsRNA-Ser-GCT	GtsRNA-Ser-GCT-4	Genomic tRNA-Ser-GCT-4	0.248	2.5E-89	*
GtsRNA-Ser-GCT	GtsRNA-Ser-GCT-5	Genomic tRNA-Ser-GCT-5	0.268	1.2E-79	*
GtsRNA-Ser-GGA	GtsRNA-Ser-GGA-1	Genomic tRNA-Ser-GGA-1	0.091	6.2E-04	
GtsRNA-Ser-TGA	GtsRNA-Ser-TGA-1	Genomic tRNA-Ser-TGA-1	0.192	1.3E-06	
GtsRNA-Ser-TGA	GtsRNA-Ser-TGA-2	Genomic tRNA-Ser-TGA-2	0.316	4.6E-53	*
GtsRNA-Sup-TCA	GtsRNA-Sup-TCA-1	Genomic tRNA-Sup-TCA-1	0.235	2.0E-33	*

sncRNA family	sncRNA subfamily	Parental RNA	*CD*	Adjusted *P*	
GtsRNA-Sup-TTA	GtsRNA-Sup-TTA-1	Genomic tRNA-Sup-TTA-1	0.353	5.4E-01	
GtsRNA-Thr-AGT	GtsRNA-Thr-AGT-1	Genomic tRNA-Thr-AGT-1	0.135	1.9E-03	
GtsRNA-Thr-AGT	GtsRNA-Thr-AGT-2	Genomic tRNA-Thr-AGT-2	0.176	2.5E-01	
GtsRNA-Thr-AGT	GtsRNA-Thr-AGT-3	Genomic tRNA-Thr-AGT-3	0.135	2.7E-01	
GtsRNA-Thr-AGT	GtsRNA-Thr-AGT-4	Genomic tRNA-Thr-AGT-4	0.373	1.0E-01	
GtsRNA-Thr-AGT	GtsRNA-Thr-AGT-5	Genomic tRNA-Thr-AGT-5	0.225	2.9E-09	*
GtsRNA-Thr-AGT	GtsRNA-Thr-AGT-6	Genomic tRNA-Thr-AGT-6	0.383	3.6E-03	*
GtsRNA-Thr-AGT	GtsRNA-Thr-AGT-7	Genomic tRNA-Thr-AGT-7	0.202	4.1E-05	*
GtsRNA-Thr-CGT	GtsRNA-Thr-CGT-1	Genomic tRNA-Thr-CGT-1	0.242	2.8E-39	*
GtsRNA-Thr-CGT	GtsRNA-Thr-CGT-2	Genomic tRNA-Thr-CGT-2	0.234	4.0E-35	*
GtsRNA-Thr-CGT	GtsRNA-Thr-CGT-3	Genomic tRNA-Thr-CGT-3	0.386	1.6E-07	*
GtsRNA-Thr-CGT	GtsRNA-Thr-CGT-4	Genomic tRNA-Thr-CGT-4	0.309	5.6E-05	*
GtsRNA-Thr-TGT	GtsRNA-Thr-TGT-1	Genomic tRNA-Thr-TGT-1	0.303	5.1E-06	*
GtsRNA-Thr-TGT	GtsRNA-Thr-TGT-2	Genomic tRNA-Thr-TGT-2	0.053	3.2E-02	
GtsRNA-Thr-TGT	GtsRNA-Thr-TGT-3	Genomic tRNA-Thr-TGT-3	0.053	3.8E-02	
GtsRNA-Trp-CCA	GtsRNA-Trp-CCA-1	Genomic tRNA-Trp-CCA-1	0.070	7.4E-05	
GtsRNA-Trp-CCA	GtsRNA-Trp-CCA-2	Genomic tRNA-Trp-CCA-2	0.044	1.0E+00	
GtsRNA-Trp-CCA	GtsRNA-Trp-CCA-3	Genomic tRNA-Trp-CCA-3	0.070	3.9E-03	
GtsRNA-Trp-CCA	GtsRNA-Trp-CCA-4	Genomic tRNA-Trp-CCA-4	0.070	3.5E-03	
GtsRNA-Trp-CCA	GtsRNA-Trp-CCA-5	Genomic tRNA-Trp-CCA-5	0.081	1.7E-05	
GtsRNA-Trp-CCA	GtsRNA-Trp-CCA-6	Genomic tRNA-Trp-CCA-6	0.070	5.0E-09	
GtsRNA-Tyr-GTA	GtsRNA-Tyr-GTA-1	Genomic tRNA-Tyr-GTA-1	0.267	7.2E-19	*
GtsRNA-Tyr-GTA	GtsRNA-Tyr-GTA-2	Genomic tRNA-Tyr-GTA-2	0.255	8.1E-20	*
GtsRNA-Tyr-GTA	GtsRNA-Tyr-GTA-3	Genomic tRNA-Tyr-GTA-3	0.256	2.5E-22	*
GtsRNA-Tyr-GTA	GtsRNA-Tyr-GTA-4	Genomic tRNA-Tyr-GTA-4	0.272	2.4E-21	*
GtsRNA-Tyr-GTA	GtsRNA-Tyr-GTA-5	Genomic tRNA-Tyr-GTA-5	0.185	4.6E-29	
GtsRNA-Val-AAC	GtsRNA-Val-AAC-1	Genomic tRNA-Val-AAC-1	0.074	1.2E-71	
GtsRNA-Val-AAC	GtsRNA-Val-AAC-2	Genomic tRNA-Val-AAC-2	0.163	5.9E-18	
GtsRNA-Val-AAC	GtsRNA-Val-AAC-3	Genomic tRNA-Val-AAC-3	0.171	7.9E-19	
GtsRNA-Val-AAC	GtsRNA-Val-AAC-4	Genomic tRNA-Val-AAC-4	0.233	1.5E-39	*
GtsRNA-Val-AAC	GtsRNA-Val-AAC-5	Genomic tRNA-Val-AAC-5	0.105	3.6E-70	
GtsRNA-Val-CAC	GtsRNA-Val-CAC-1	Genomic tRNA-Val-CAC-1	0.091	1.4E-71	
GtsRNA-Val-CAC	GtsRNA-Val-CAC-2	Genomic tRNA-Val-CAC-2	0.073	2.3E-71	
GtsRNA-Val-CAC	GtsRNA-Val-CAC-3	Genomic tRNA-Val-CAC-3	0.171	4.6E-20	
GtsRNA-Val-CAC	GtsRNA-Val-CAC-4	Genomic tRNA-Val-CAC-4	0.084	2.0E-71	
GtsRNA-Val-CAC	GtsRNA-Val-CAC-5	Genomic tRNA-Val-CAC-5	0.233	1.0E+00	

sncRNA family	sncRNA subfamily	Parental RNA	*CD*	Adjusted *P*	
GtsRNA-Val-CAC	GtsRNA-Val-CAC-7	Genomic tRNA-Val-CAC-7	0.595	1.3E-17	*
GtsRNA-Val-TAC	GtsRNA-Val-TAC-1	Genomic tRNA-Val-TAC-1	0.038	1.0E+00	
MtsRNA-Ala-TGC	MtsRNA-Ala-TGC-1	Mitochondrial tRNA-Ala-TGC-1	0.327	1.2E-03	*
MtsRNA-Arg-TCG	MtsRNA-Arg-TCG-1	Mitochondrial tRNA-Arg-TCG-1	0.532	1.8E-53	*
MtsRNA-Arg-TCG	MtsRNA-Arg-TCG-2	Mitochondrial tRNA-Arg-TCG-2	0.549	2.5E-45	*
MtsRNA-Arg-TCG	MtsRNA-Arg-TCG-3	Mitochondrial tRNA-Arg-TCG-3	0.423	3.5E-09	*
MtsRNA-Arg-TCG	MtsRNA-Arg-TCG-4	Mitochondrial tRNA-Arg-TCG-4	0.399	3.8E-04	*
MtsRNA-Asn-GTT	MtsRNA-Asn-GTT-1	Mitochondrial tRNA-Asn-GTT-1	0.518	5.5E-08	*
MtsRNA-Cys-GCA	MtsRNA-Cys-GCA-1	Mitochondrial tRNA-Cys-GCA-1	0.274	1.5E-07	*
MtsRNA-Cys-GCA	MtsRNA-Cys-GCA-2	Mitochondrial tRNA-Cys-GCA-2	0.375	1.0E+00	
MtsRNA-Cys-GCA	MtsRNA-Cys-GCA-3	Mitochondrial tRNA-Cys-GCA-3	0.298	1.0E+00	
MtsRNA-Gln-TTG	MtsRNA-Gln-TTG-1	Mitochondrial tRNA-Gln-TTG-1	0.127	1.0E+00	
MtsRNA-Gln-TTG	MtsRNA-Gln-TTG-2	Mitochondrial tRNA-Gln-TTG-2	0.208	2.6E-03	*
MtsRNA-Gln-TTG	MtsRNA-Gln-TTG-3	Mitochondrial tRNA-Gln-TTG-3	0.153	6.5E-01	
MtsRNA-Glu-TTC	MtsRNA-Glu-TTC-1	Mitochondrial tRNA-Glu-TTC-1	0.241	5.2E-80	*
MtsRNA-Glu-TTC	MtsRNA-Glu-TTC-2	Mitochondrial tRNA-Glu-TTC-2	0.257	2.6E-77	*
MtsRNA-Glu-TTC	MtsRNA-Glu-TTC-3	Mitochondrial tRNA-Glu-TTC-3	0.233	8.7E-33	*
MtsRNA-Gly-TCC	MtsRNA-Gly-TCC-1	Mitochondrial tRNA-Gly-TCC-1	0.129	1.6E-12	
MtsRNA-Gly-TCC	MtsRNA-Gly-TCC-2	Mitochondrial tRNA-Gly-TCC-2	0.134	1.2E-13	
MtsRNA-His-GTG	MtsRNA-His-GTG-1	Mitochondrial tRNA-His-GTG-1	0.045	8.5E-49	
MtsRNA-His-GTG	MtsRNA-His-GTG-2	Mitochondrial tRNA-His-GTG-2	0.050	7.7E-46	
MtsRNA-Ile-GAT	MtsRNA-Ile-GAT-1	Mitochondrial tRNA-Ile-GAT-1	0.248	1.0E+00	
MtsRNA-Ile-GAT	MtsRNA-Ile-GAT-2	Mitochondrial tRNA-Ile-GAT-2	0.239	1.0E+00	
MtsRNA-Leu-TAA	MtsRNA-Leu-TAA-1	Mitochondrial tRNA-Leu-TAA-1	0.263	4.1E-14	*
MtsRNA-Leu-TAG	MtsRNA-Leu-TAG-1	Mitochondrial tRNA-Leu-TAG-1	0.693	7.2E-01	
MtsRNA-Lys-TTT	MtsRNA-Lys-TTT-1	Mitochondrial tRNA-Lys-TTT-1	0.245	7.1E-01	
MtsRNA-Met-CAT	MtsRNA-Met-CAT-1	Mitochondrial tRNA-Met-CAT-1	0.284	1.8E-26	*
MtsRNA-Phe-GAA	MtsRNA-Phe-GAA-1	Mitochondrial tRNA-Phe-GAA-1	1.000	1.6E-02	*
MtsRNA-Phe-GAA	MtsRNA-Phe-GAA-2	Mitochondrial tRNA-Phe-GAA-2	1.000	1.6E-02	*
MtsRNA-Pro-TGG	MtsRNA-Pro-TGG-1	Mitochondrial tRNA-Pro-TGG-1	0.506	1.0E-69	*
MtsRNA-Pro-TGG	MtsRNA-Pro-TGG-2	Mitochondrial tRNA-Pro-TGG-2	0.564	1.4E-73	*
MtsRNA-Pro-TGG	MtsRNA-Pro-TGG-3	Mitochondrial tRNA-Pro-TGG-3	0.544	3.1E-61	*
MtsRNA-Ser-GCT	MtsRNA-Ser-GCT-1	Mitochondrial tRNA-Ser-GCT-1	0.054	1.0E+00	
MtsRNA-Ser-GCT	MtsRNA-Ser-GCT-2	Mitochondrial tRNA-Ser-GCT-2	0.064	1.0E+00	
MtsRNA-Ser-TGA	MtsRNA-Ser-TGA-1	Mitochondrial tRNA-Ser-TGA-1	0.145	3.4E-01	
MtsRNA-Thr-TGT	MtsRNA-Thr-TGT-1	Mitochondrial tRNA-Thr-TGT-1	0.060	1.8E-01	

sncRNA family	sncRNA subfamily	Parental RNA	*CD*	Adjusted *P*	
MtsRNA-Thr-TGT	MtsRNA-Thr-TGT-2	Mitochondrial tRNA-Thr-TGT-2	0.064	4.0E-02	
MtsRNA-Thr-TGT	MtsRNA-Thr-TGT-3	Mitochondrial tRNA-Thr-TGT-3	0.068	1.0E+00	
MtsRNA-Trp-TCA	MtsRNA-Trp-TCA-1	Mitochondrial tRNA-Trp-TCA-1	0.539	1.2E-30	*
MtsRNA-Trp-TCA	MtsRNA-Trp-TCA-2	Mitochondrial tRNA-Trp-TCA-2	0.539	1.2E-30	*
MtsRNA-Tyr-GTA	MtsRNA-Tyr-GTA-1	Mitochondrial tRNA-Tyr-GTA-1	0.213	8.9E-01	
MtsRNA-Val-TAC	MtsRNA-Val-TAC-1	Mitochondrial tRNA-Val-TAC-1	0.464	3.5E-110	*
MtsRNA-Val-TAC	MtsRNA-Val-TAC-2	Mitochondrial tRNA-Val-TAC-2	0.463	4.9E-20	*
MtsRNA-Val-TAC	MtsRNA-Val-TAC-3	Mitochondrial tRNA-Val-TAC-3	0.480	3.1E-132	*
rsRNA-4.5S	rsRNA-4.5S	4.5S rRNA	0.146	1.8E-02	
rsRNA-5S	rsRNA-5S	5S rRNA	0.415	3.6E-219	*
rsRNA-5.8S	rsRNA-5.8S	5.8S rRNA	0.195	2.9E-55	
rsRNA-12S	rsRNA-12S	12S rRNA	0.301	2.9E-02	*
rsRNA-16S	rsRNA-16S	16S rRNA	0.196	1.4E-10	
rsRNA-18S	rsRNA-18S	18S rRNA	0.182	2.6E-77	
rsRNA-28S	rsRNA-28S	28S rRNA	0.290	2.7E-262	*
rsRNA-45S	rsRNA-45S	45S rRNA	0.524	6.2E-58	*

Note - Differential fragmentation analysis was performed using *qMAP* Model 1. *: differentially fragmented parental RNAs; *CD*: coverage difference.
