# Supplementary material for: qMAP decodes RNA fragmentation dynamics in development and disease
Source: Mol Syst Biol. 2026 Jul 20;22(10):1626–49. doi: 10.1038/s44320-026-00237-2 (PMC13638987; doi:10.1038/s44320-026-00237-2)
Supplement: Supplementary file 7 [file 44320_2026_237_MOESM6_ESM.pdf]

molecular systems biology

qMAP decodes RNA fragmentation dynamics in development and disease

Hukam Rawal, Jiancheng Yu, Xudong Zhang, Chen Cai, Qi Chen, and Tong Zhou

Corresponding author(s): Tong Zhou (tongz@med.unr.edu) , Qi Chen (qi.chen@hsc.utah.edu)

Review Timeline:

Submission Date:	25th Feb 26
Editorial Decision:	13th Apr 26
Revision Received:	14th May 26
Editorial Decision:	16th Jun 26
Revision Received:	30th Jun 26
Accepted:	7th Jul 26

Editor: Poonam Bheda

Transaction Report:

(Note: With the exception of the correction of typographical or spelling errors that could be a source of ambiguity, letters and reports are not edited. Depending on transfer agreements, referee reports obtained elsewhere may or may not be included in this compilation. Referee reports are anonymous unless the Referee chooses to sign their reports.)

13th Apr 2026

Manuscript Number: MSB-2026-13661
Title: qMAP decodes RNA fragmentation dynamics in development and disease

Dear Dr. Zhou,

Thank you again for submitting your work to Molecular Systems Biology. We have now heard back from the three reviewers who agreed to evaluate your study. As you will see below, the reviewers appreciate that the proposed analysis of RNA fragmentation patterns is interesting. However, they raise a series of concerns, which we would ask you to address in a major revision.

Without repeating all the comments listed below, some of the more fundamental issues raised are the following:
- small tRNA-derived fragments are ambiguous in terms of which tRNA they are derived
- RNA modification-related sequencing bias not addressed
- Descriptive due to lack of validation that RNA fragmentation patterns are biologically informative

These issues would need to be addressed in full in order for a successful revision at Molecular Systems Biology. All other issues raised would need to be satisfactorily addressed. Please let me know in case you would like to discuss in further detail any of the comments, I would be happy to schedule a call.

When submitting your revised manuscript, please carefully review the instructions that follow below. We perform an initial quality control of all revised manuscripts before re-review; failure to include requested items will delay the evaluation of your revision.

We require:

1) A .docx formatted version of the manuscript text (including legends for main figures, EV figures and tables). Please make sure that the changes are highlighted to be clearly visible. Alternatively you may choose to submit your manuscript as a LaTeX file.

2) Individual production quality figure files as .eps, .tif, .jpg (one file per figure). For guidance, download the 'Figure Guide PDF' (https://media.springernature.com/original/springer-cms/rest/v1/content/27825798/data/v1).

3) At EMBO Press we ask authors to provide source data for the main figures. Our source data coordinator will contact you to discuss which figure panels we would need source data for and will also provide you with helpful tips on how to upload and organize the files.

4) A .docx formatted letter INCLUDING the reviewers' reports and your detailed point-by-point responses to their comments. As part of the EMBO Press transparent editorial process, the point-by-point response is part of the Peer Review File (PRF), which will be published alongside your paper.

5) A complete author checklist, which you can download from our author guidelines (https://link.springer.com/journal/44320/submission-guidelines#cms). Please insert information in the checklist that is also reflected in the manuscript. The completed author checklist will also be part of the PRF.

6) Please note that all corresponding authors are required to supply an ORCID ID for their name upon submission of a revised manuscript.

7) It is mandatory to include a 'Data Availability' section after the Materials and Methods. Before submitting your revision, primary datasets produced in this study need to be deposited in an appropriate public database, and the accession numbers and database listed under 'Data Availability'. Please remember to provide a reviewer password if the datasets are not yet public (see https://link.springer.com/partners/embo-press/editorial-policies#Data%20availability%20statement).

In case you have no data that requires deposition in a public database, please state so in this section as follows: "This study includes no data deposited in external repositories". Note that the Data Availability Section is restricted to new primary data that are part of this study.

8) All Materials and Methods need to be described in the main text using our 'Structured Methods' format, which is required for all research articles. According to this format, the Methods section includes a Reagents and Tools Table (listing key reagents, experimental models, software and relevant equipment and including their sources and relevant identifiers) followed by a Methods and Protocols section describing the methods using a step-by-step protocol format. The aim is to facilitate adoption of the methodologies across labs. Please upload the Reagents and Tools table as a separate document when submitting your revised manuscript. More information on how to adhere to this format as well as a downloadable template (.docx) for the Reagents and Tools Table can be found in our author guidelines: https://link.springer.com/journal/44320/submission-guidelines#cms

9) For data quantification: please specify the name of the statistical test used to generate error bars and p-values, the number (n) of independent experiments (specify technical or biological replicates) underlying each data point and the test used to calculate p-values in each figure legend. The figure legends should contain a basic description of n, p-values and the test applied. Graphs must include a description of the bars and the error bars (s.d., s.e.m.). Please provide exact p-values (in either the figure or figure legend).

10) Our journal encourages inclusion of *data citations in the reference list* to directly cite datasets that were re-used and obtained from public databases. Data citations in the article text are distinct from normal bibliographical citations and should directly link to the database records from which the data can be accessed. In the main text, data citations are formatted as follows: "Data ref: Smith et al, 2001" or "Data ref: NCBI Sequence Read Archive PRJNA342805, 2017". In the Reference list, data citations must be labeled with "[DATASET]". A data reference must provide the database name, accession number/identifiers and a resolvable link to the landing page from which the data can be accessed at the end of the reference. Further instructions are available at .

11) We replaced Supplementary Information with Expanded View (EV) Figures and Tables that are collapsible/expandable online. EV Figures should be cited as 'Figure EV1, Figure EV2" etc... in the text and their respective legends should be included in the main text after the legends of regular figures.
- For the figures that you do NOT wish to display as Expanded View figures, they should be bundled together with their legends in a single PDF file called *Appendix*, which should start with a short Table of Content. Appendix figures should be referred to in the main text as: "Appendix Figure S1, Appendix Figure S2" etc.
- Additional Tables/Datasets should be labeled and referred to as Table EV1, Dataset EV1, etc. Legends should be provided in a separate tab in case of .xls files. Alternatively, the legend can be supplied as a separate text file (README) and zipped together with the Table/Dataset file.
See detailed instructions here:
https://link.springer.com/journal/44320/submission-guidelines#cms

12) Author contributions: CRediT has replaced the traditional author contributions section because it offers a systematic machine-readable author contributions format that allows for more effective research assessment. Please remove the Authors Contributions from the manuscript and use the free text boxes beneath each contributing author's name in our system to add specific details on the author's contribution. More information is available in our guide to authors.

13) Disclosure statement and competing interests: We updated our journal's competing interests policy in January 2022 and request authors to consider both actual and perceived competing interests. Please review the policy https://link.springer.com/partners/embo-press/editorial-policies#Competing%20interest%20disclosures and update your competing interests if necessary.

14) Every published paper now includes a 'Synopsis' to further enhance discoverability. Synopses are displayed on the journal webpage and are freely accessible to all readers. They include a short stand first (maximum of 300 characters, including space) as well as 2-5 one-sentences bullet points that summarizes the paper. Please write the bullet points to summarize the key NEW findings. They should be designed to be complementary to the abstract - i.e. not repeat the same text. We encourage inclusion of key acronyms and quantitative information (maximum of 30 words / bullet point). Please use the passive voice. Please attach these in a separate file or send them by email, we will incorporate them accordingly.

Please also suggest a striking image or visual abstract to illustrate your article as a PNG file 550 px wide x 300 px high.

Please note that these would be the final versions and changes during proofing are usually not allowed.

15) As part of the EMBO Publications transparent editorial process initiative (see our policy here: https://link.springer.com/partners/embo-press/editorial-policies#Peer%20review), Molecular Systems Biology will publish online a Peer Review File (PRF) to accompany accepted manuscripts.
In the event of acceptance, this file will be published in conjunction with your paper and will include the anonymous referee reports, your point-by-point response and all pertinent correspondence relating to the manuscript. Let us know whether you agree with the publication of the PRF and as here, if you want to remove or not any figures from it prior to publication.
Please note that the Author checklist will be published at the end of the PRF.

Molecular Systems Biology has a "scooping protection" policy, whereby similar findings that are published by others during review or revision are not a criterion for rejection. Should you decide to submit a revised version, I do ask that you get in touch after three months if you have not completed it, to update us on the status.

Yours sincerely,

Poonam Bheda, PhD
Scientific Editor

Reviewer #1:

The authors develop qMAP, a computational approach that identifies RNA fragmentation patterns from small RNA-seq data. The method maps small RNA reads to parental RNAs (e.g., tRF reads to tRNAs) and calculates normalized coverage along the parental RNA, allowing RNA fragmentation patterns to be compared across conditions. The motivation is clear - in small RNA field, novel small RNA fragments from tRNAs, rRNAs and more are being reported and often times their fragmentation pattern (not the expression level) are the key to drive the specificity. Using publicly available data, the authors show that fragmentation patterns change during mouse embryonic fibroblast (MEF) reprogramming, distinguish ulcerative colitis (UC) disease states, and produce a blood-based RNA fragmentation signature for recurrent implantation failure (RIF). These results support the concept of an "RNA fragmentome," where RNA fragmentation patterns represent a regulatory layer that captures clinical and biological variation beyond RNA abundance alone.
Major points:
(1) For the tRNA fragments examples, the authors focus on mapping to mature tRNAs, but tRNA fragments could also come from pre-tRNAs (tRF-1s). Can qMAP be extended to calculate fragmentation pattern from pre-tRNAs, or identify difference in mature vs pre-tRNA portions? That will greatly expand the tools applications.
(2) Many tRFs map equally well to multiple tRNA isodecoders that share identical sequences across portions of the tRNA molecule. Because qMAP quantifies fragmentation patterns along specific parental tRNAs, ambiguous assignment of multi-annotated reads could influence the inferred coverage profiles. In many cases, it is not possible to definitively determine which parental tRNA generated a fragment if the isodecoders share identical sequences in that region. The manuscript does not describe how such multi-annotated reads are handled. Clarifying whether these reads are discarded, randomly assigned, or fractionally distributed across potential parental tRNAs would strengthen confidence in the reported fragmentation patterns.
(3) tRNAs contain numerous nucleotide modifications that can disrupt base-pairing and interfere with reverse transcription during library preparation, leading to incomplete detection of many small RNA fragments. Because qMAP infers fragmentation patterns based on relative coverage along parental RNAs, such sequencing biases could influence the fragmentation profiles. While the authors acknowledge this issue and note that methods such as PANDORA-seq can mitigate modification-related bias, only the MEF reprogramming dataset was generated using PANDORA-seq. The UC and RIF datasets were generated using conventional small RNA-seq library preparation methods, which may fail to capture heavily modified small RNAs. Clarifying that qMAP itself does not correct for modification-related sequencing bias, and that optimal application may require modification-aware sequencing methods, would help readers interpret these results appropriately.

Minor points:
(1) As a computational pipeline, it will be helpful to include an example dataset input and output in the github page. It is unclear if the output file contains the coverage difference at each position.
(2) In the section describing the RIF cohort, it was unclear whether the samples were collected from endometrial/menstrual blood or peripheral blood, and the clinical context of the implantation was not immediately clear (i.e., what exactly was being implanted?). While consulting the GEO dataset description helped determine this information, explicitly stating that the samples are peripheral blood and that the implantation refers to embryo implants would improve clarity.

Reviewer #2:

In general terms, I think this is an important manuscript because it shows, in different systems, that looking at how noncoding RNAs such as tRNAs and rRNAs are fragmented can be more informative than abundance-based estimations of the expression of the parental RNA based on the number of reads aligning to that RNA.

My main concern, and I would like to hear the author's thoughts and whether they see what I will explain next as a limitation of their study, is that small tRNA-derived fragments are ambiguous in terms of which tRNA are they derived from. And this affects both abundance-based estimations, and the author's proposed fragmentation index.

For example, let say that the authors see a shift in the fragmentation pattern of tRNA-X between condition A and condition B, with condition A mostly expressing 5' fragments, and condition B mostly expressing 3' fragments. However, the same results would be obtained if condition B produces massive amounts of tRNA-Y, whose 3' fragments look exactly the same as the 3' fragments from tRNA-X. In this situation, a change in tRNA abundance (upregulation of tRNA-Y in the context of constant levels of tRNA-X) would be interpreted as a change in tRNA-X's fragmentation profile.

I don't expect the authors to solve this problem in this paper, but I suggest adding a cautionary note in how difficult it can be to infer RNA fragmentation profiles using small RNA sequencing in the context of highly conserved and highly fragmented noncoding RNAs.

Juan Pablo Tosar

Reviewer #3:

The manuscript introduces qMAP, a computational framework to quantify RNA fragmentation patterns and advances the concept of an "RNA fragmentome" as a regulatory layer. The idea is interesting and potentially impactful, particularly in moving beyond abundance-based analyses of small RNA sequencing data. However, in its current form, the manuscript does not yet provide sufficient support for its central claims.

While the computational framework appears technically sound and is demonstrated on several datasets, the evidence remains largely descriptive. The analyses focus on identifying differential fragmentation patterns, but there is limited demonstration that these patterns correspond to functional or mechanistic consequences. As a result, the broader biological interpretation, that fragmentation encodes regulatory information comparable to alternative splicing, feels premature.

Relatedly, the manuscript makes several strong claims regarding biological relevance and potential clinical applications, including biomarker discovery. These claims would require more direct validation. At present, the study relies entirely on computational analysis of existing datasets, and the absence of experimental validation (or orthogonal validation on independent datasets) makes it difficult to assess the robustness and generality of the conclusions. The identification of candidate fragments is promising, but their functional significance remains to be demonstrated.

The clarity of the manuscript could also be improved. Some aspects of the framework, particularly the definition and interpretation of fragmentation patterns and the statistical methodology, are not always presented in a sufficiently transparent way. In addition, the narrative occasionally blends current results with forward-looking statements, which can make it difficult to distinguish demonstrated findings from speculative implications.

Overall, the work presents an appealing idea and a potentially useful tool, but additional validation and clearer presentation would be needed to support the broader claims and make the study suitable for publication.

Response to the Editor's highlighted comments

- small tRNA-derived fragments are ambiguous in terms of which tRNA they are derived

Response: Regarding the ambiguous parental assignment of short tRNA-derived fragments**,** we now clarify that this is an inherent limitation of short-read sncRNA-seq for highly conserved tRNA families. *qMAP* does not claim unique locus/isodecoder assignment for fragments that map equally well to multiple parental templates. We acknowledged this limitation in the Discussion/Limitations section. Full details are provided in our responses to **Reviewer 1's tRNA multi-mapping comment** and **Reviewer 2's main concern**.

- RNA modification-related sequencing bias not addressed

Response: Regarding RNA modification-related sequencing bias**,** we now explicitly state that *qMAP* is a computational framework and does not correct for fragments missed or underrepresented because of RNA modifications or library-preparation bias. We clarify that RNA fragmentation patterns profiled from conventional small RNA-seq datasets represent the detectable fragment population under that protocol, and that modification-aware approaches, such as PANDORA-seq, are preferable for comprehensive analysis of highly modified sncRNAs. Full details are provided in our response to **Reviewer 1 (Comment 3)** and the revised Methods/Discussion section.

- Descriptive due to lack of validation that RNA fragmentation patterns are biologically informative

Response: In this revision, we have included an additional investigation regarding sperm aging. *qMAP* identified an aging-associated fragmentation remodeling and prioritized one 17-nt rsRNA and one 44-nt rsRNA from the same 28S rRNA locus for functional test. We transfected these two rsRNAs into mESCs and performed mRNA-seq, revealing distinct transcriptomic responses between the 17-nt and 44-nt rsRNA transfections. These new data are now included in the revised Results/Methods sections as well as **Fig. 6** and **Fig. EV6**. Full details are provided in our response to **Reviewer 3** and **Reviewer 1 (Comment 3)**.

Together, these revisions clarify the methodological limitations, improve interpretation of *qMAP* results across sequencing platforms, and provide proof-of-principle functional evidence that *qMAP*-prioritized RNA fragments exhibit distinct biological effects. Please find more details in our **Response to the Reviewers' comments** below.

Point-to-point responses to the Reviewers' comments

Reviewer #1:

The authors develop qMAP, a computational approach that identifies RNA fragmentation patterns from small RNA-seq data. The method maps small RNA reads to parental RNAs (e.g., tRF reads to tRNAs) and calculates normalized coverage along the parental RNA, allowing RNA fragmentation patterns to be compared across conditions. The motivation is clear - in small RNA field, novel small RNA fragments from tRNAs, rRNAs and more are being reported and often times their fragmentation pattern (not the expression level) are the key to drive the specificity. Using publicly available data, the authors show that fragmentation patterns change during mouse embryonic fibroblast (MEF) reprogramming, distinguish ulcerative colitis (UC) disease states, and produce a blood-based RNA fragmentation signature for recurrent implantation failure (RIF). These results support the concept of an "RNA fragmentome," where RNA fragmentation patterns represent a regulatory layer that captures clinical and biological variation beyond RNA abundance alone.

Major points:

(1) For the tRNA fragments examples, the authors focus on mapping to mature tRNAs, but tRNA fragments could also come from pre-tRNAs (tRF-1s). Can qMAP be extended to calculate fragmentation pattern from pre-tRNAs, or identify difference in mature vs pre-tRNA portions? That will greatly expand the tools applications.

Response: We thank the reviewer for this important suggestion. We agree with the reviewer that tRNA-derived fragments can originate not only from mature tRNAs but also from pre-tRNAs, including 5' leaders, 3' trailers, introns, and other precursor-specific regions. *qMAP* is not restricted to mature tRNA or rRNA annotations. Because *qMAP* calculates normalized coverage along user-defined parental RNA templates, it can be applied to pre-tRNA templates as long as the corresponding precursor sequences and annotations are provided. In this revision, we have added an Expanded View Figure (**Fig. EV7**) to demonstrate this capability, showing the examples of differential fragmentation of pre-tRNAs during cell reprogramming from MEFs to intermediates and finally to iPSCs. The relevant details have been included in the Methods section. The discussion and description for **Fig. EV7** is also included in the Discussion section as *"Because qMAP operates on user-defined parental RNA templates, it is also able to identify the difference in fragmentation for the reads derived from the precursors of parental RNAs. Some tsRNAs (named as tRFs or fitRNAs in these original studies) have been reported to be derived from 3' trailers and introns of pre-tRNAs with function (Lee et al, 2009; Nostramo et al, 2025). As an example, qMAP detected differential fragmentation of pre-GtsRNA-His-GTG-2-2, pre-GtsRNA-Leu-CAA-1-1, and pre-GtsRNA-Tyr-GTA-2-1 during cell reprogramming from MEFs to intermediates, and iPSCs (Fig. EV7)."*

(2) Many tRFs map equally well to multiple tRNA isodecoders that share identical sequences across portions of the tRNA molecule. Because qMAP quantifies fragmentation patterns along specific parental tRNAs, ambiguous assignment of multi-annotated reads could influence the inferred coverage profiles. In many cases, it is not possible to definitively determine which parental tRNA generated a fragment if the isodecoders share identical sequences in that region. The manuscript does not describe how such multi-annotated reads are handled. Clarifying whether these reads are discarded, randomly assigned, or fractionally distributed across potential parental tRNAs would strengthen confidence in the reported fragmentation patterns.

Response: We thank the reviewer for this important and constructive comment. We agree with the reviewer that some tRNA-derived fragments can be mapped to multiple parental tRNAs or isodecoders, particularly when the fragment lies within a sequence region shared among related tRNAs. This issue is intrinsic to short-read sncRNA-seq analysis: if a short fragment sequence is identical among several possible parental tRNA templates, the sequence alone does not contain sufficient information to determine its true parental origin. In *qMAP*, the coverage pattern was calculated based on all the sncRNA species that can be mapped to a given RNA template. Therefore, a sncRNA species that maps equally well to multiple parental tRNAs is retained and contributes to the normalized coverage of each compatible parental template (we did not randomly assign reads to one template or fractionally divided among templates). We chose this strategy because each alternative also introduces a different bias. Discarding multi-mapped fragments would remove a substantial and biologically meaningful portion of the tRF/tsRNA population, especially fragments derived from conserved tRNA regions. Random assignment would introduce stochastic variation and reduce reproducibility. Fractional assignment would require the assumption that each compatible parental tRNA contributed equally, which cannot be inferred from the short fragment sequence itself and may artificially dilute condition-specific signals. Thus, no strategy can uniquely resolve parental origin for identical short sequences without additional orthogonal information. On the other hand, because the same annotation and mapping rules are applied consistently across all samples and conditions, we believe *qMAP* remains suitable for detecting reproducible condition-associated changes in fragmentation patterns under a defined annotation framework. Nonetheless, we now explicitly discuss this limitation in the Discussion section to remind readers on the potential caveats (which is also advised by reviewer 2), which reads as *"One limitation shared by qMAP and other short-read sncRNA-seq analyses is the ambiguity of parental-RNA assignment for fragments derived from highly conserved or repetitive RNA families, especially tRNAs. Many tRNA-derived fragments are identical across multiple tRNA genes or isodecoders, making their true parental origin impossible to determine from the fragment sequence alone. qMAP does not attempt to assign such fragments to a single unique parental origin. Instead, for each parental RNA template, normalized coverage was calculated using all annotated sncRNA species compatible with that template, thus these profiles should be interpreted as template-compatible fragmentation patterns rather than definitive locus- or isodecoder-specific cleavage maps. Alternative strategies, such as discarding multi-mapped reads, randomly assigning them, or fractionally distributing them across compatible templates, also introduce*

distinct biases. This ambiguity is therefore an inherent limitation of short-read fragment mapping, not specific to qMAP. Future integration of longer reads, genetic variants, tRNA modification profiles, precursor-specific features, or orthogonal perturbation experiments may help resolve parental origin in selected cases."

(3) tRNAs contain numerous nucleotide modifications that can disrupt base-pairing and interfere with reverse transcription during library preparation, leading to incomplete detection of many small RNA fragments. Because qMAP infers fragmentation patterns based on relative coverage along parental RNAs, such sequencing biases could influence the fragmentation profiles. While the authors acknowledge this issue and note that methods such as PANDORA-seq can mitigate modification-related bias, only the MEF reprogramming dataset was generated using PANDORA-seq. The UC and RIF datasets were generated using conventional small RNA-seq library preparation methods, which may fail to capture heavily modified small RNAs. Clarifying that qMAP itself does not correct for modification-related sequencing bias, and that optimal application may require modification-aware sequencing methods, would help readers interpret these results appropriately.

Response: We thank the reviewer for raising this important point. We agree with the reviewer that RNA modifications, particularly those enriched in tRNAs and tRNA-derived fragments, can interfere with adapter ligation, reverse transcription, and library construction, leading to incomplete or biased detection of small RNA fragments, that was our motive to develop PANDORA-seq to overcome these biases (Nat Cell Biol 2021 PMID: 33820973). We also agree that *qMAP* itself does not correct for such modification-related sequencing bias. *qMAP* is a computational framework that quantifies fragmentation patterns from the mapped sncRNA species detected in a given sncRNA-seq dataset. Therefore, if a fragment is not captured or is underrepresented because of library-preparation bias, *qMAP* cannot computationally recover that missing signal. We have now clarified this point in the revised manuscript, stating that *qMAP* profiles generated from conventional sncRNA-seq datasets should be interpreted as fragmentation patterns of the detectable fragment population under the corresponding library-preparation protocol, rather than as a complete representation of the entire RNA fragmentome *in vivo*. At the same time, *qMAP* can still be applied to conventional small RNA-seq datasets, provided that the results are interpreted appropriately. In the UC and RIF analyses, all samples within each dataset were generated using the same library-preparation strategy, allowing *qMAP* to identify condition-associated fragmentation differences within a consistent technical framework. We have added a paragraph in the method section "*Differential fragmentation analysis using qMAP*" to clarify this to avoid misunderstanding: *"To be noted, qMAP quantifies fragmentation patterns from the sncRNA species detected and annotated in each sequencing dataset. It does not model or correct for library-preparation biases caused by RNA modifications, adapter-ligation efficiency, reverse-transcription arrest, or amplification efficiency. Thus, reported fragmentation profiles represent the detectable sncRNA population under the sequencing protocol being used."* For heavily modified tRNA-derived or rRNA-derived fragments, modification-aware approaches such as

PANDORA-seq would be optimal for comprehensive *qMAP* analysis of tsRNAs and rsRNAs. In the revised manuscript, we have included one more PANDORA-seq dataset to illustrate the power of *qMAP* in identifying differential fragmentation during sperm aging. This dataset is from our newly published paper (EMBO J 2026, PMID: 41559200). *qMAP* recapitulated the previously observed rsRNA length shift from the perspective of rRNA-centered fragmentation remodeling and prioritized short and long rsRNAs with distinct effects on programming mESC gene expression. These data have been included in the Results section along with a new figure (**Fig. 6**) in the main text and one more Expanded View Figure (**Fig. EV6**).

Minor points:

(1) As a computational pipeline, it will be helpful to include an example dataset input and output in the github page. It is unclear if the output file contains the coverage difference at each position.

Response: Thanks for the suggestion. The example input/output datasets are included in the "/inst/extdata" folder at GitHub (https://github.com/cozyrna/qMAP/tree/main/inst/extdata).

(2) In the section describing the RIF cohort, it was unclear whether the samples were collected from endometrial/menstrual blood or peripheral blood, and the clinical context of the implantation was not immediately clear (i.e., what exactly was being implanted?). While consulting the GEO dataset description helped determine this information, explicitly stating that the samples are peripheral blood and that the implantation refers to embryo implants would improve clarity.

Response: Thanks for the careful reading. In the context of RIF, we have now clarified that "implantation" refers to "embryo implantation". And for blood, it refers to peripheral whole blood samples, not endometrial or menstrual blood. We have clarified "peripheral whole blood" in the manuscript context. We have also updated the result section to explain the patient situation in more details: *"The RIF patients were characterized by having undergone at least three unsuccessful in vitro fertilization cycles with embryo transfers (Rekker et al, 2018)."*

Reviewer #2:

In general terms, I think this is an important manuscript because it shows, in different systems, that looking at how noncoding RNAs such as tRNAs and rRNAs are fragmented can be more informative than abundance-based estimations of the expression of the parental RNA based on the number of reads aligning to that RNA.

Response: Many thanks, Juan. We very much appreciate your positive comments and the constructive suggestions.

My main concern, and I would like to hear the author's thoughts and whether they see what I will explain next as a limitation of their study, is that small tRNA-derived fragments are ambiguous in terms of which tRNA are they derived from. And this affects both abundance-based estimations, and the author's proposed fragmentation index.

For example, let say that the authors see a shift in the fragmentation pattern of tRNA-X between condition A and condition B, with condition A mostly expressing 5' fragments, and condition B mostly expressing 3' fragments. However, the same results would be obtained if condition B produces massive amounts of tRNA-Y, whose 3' fragments look exactly the same as the 3' fragments from tRNA-X. In this situation, a change in tRNA abundance (upregulation of tRNA-Y in the context of constant levels of tRNA-X) would be interpreted as a change in tRNA-X's fragmentation profile.

I don't expect the authors to solve this problem in this paper, but I suggest adding a cautionary note in how difficult it can be to infer RNA fragmentation profiles using small RNA sequencing in the context of highly conserved and highly fragmented noncoding RNAs.

Response: We thank the reviewer for this important comment (which is similarly mentioned by Reviewer 1, comment 2). We agree with the reviewer that some tRNA-derived fragments can be mapped to multiple parental tRNA genes or isodecoders, particularly when the fragment lies within a sequence region shared among related tRNAs. This ambiguity affects both abundance-based and fragmentation-based analyses of tRNA-derived small RNAs and is not fully resolvable from short-read sncRNA-seq data alone. In *qMAP*, the coverage pattern was calculated based on all the sncRNA species that can be mapped to a given RNA template. Therefore, a tsRNA species that maps equally well to multiple parental tRNAs is retained and contributes to the normalized coverage of each compatible parental template (we did not randomly assign tsRNA to one template or fractionally divided among templates). We chose this strategy because each alternative also introduces a different bias. Discarding multi-mapped fragments would remove a substantial and biologically meaningful portion of the tRF/tsRNA population, especially for the fragments derived from conserved tRNA regions. Random assignment would introduce stochastic variation and reduce reproducibility. Fractional assignments require the assumption that each compatible parental tRNA contributed equally, which cannot be inferred from the short fragment sequence itself and may artificially dilute condition-specific signals. Thus, no strategy can uniquely resolve parental origin for identical short sequences without additional orthogonal information. To acknowledge this intrinsic limitation, we have now explicitly discussed this in the Discussion section to remind readers on this potential caveats, which reads as *"One limitation shared by qMAP and other short-read sncRNA-seq analyses is the ambiguity of parental-RNA assignment for fragments derived from highly conserved or repetitive RNA families, especially tRNAs. Many tRNA-derived fragments are identical across multiple tRNA genes or isodecoders, making their true parental origin impossible to determine from the fragment sequence alone. qMAP does not attempt to assign such fragments to a single unique parental origin. Instead, for each parental RNA template, normalized coverage was calculated using all annotated sncRNA species compatible with that template, thus these profiles should be interpreted as template-compatible fragmentation patterns rather than definitive locus- or isodecoder-specific cleavage maps. Alternative strategies, such as discarding multi-mapped reads, randomly assigning them, or fractionally distributing them across compatible templates, also introduce distinct biases. This ambiguity is therefore an inherent limitation of short-read fragment mapping, not specific to*

qMAP. Future integration of longer reads, genetic variants, tRNA modification profiles, precursor-specific features, or orthogonal perturbation experiments may help resolve parental origin in selected cases."

Reviewer #3:

The manuscript introduces qMAP, a computational framework to quantify RNA fragmentation patterns and advances the concept of an "RNA fragmentome" as a regulatory layer. The idea is interesting and potentially impactful, particularly in moving beyond abundance-based analyses of small RNA sequencing data. However, in its current form, the manuscript does not yet provide sufficient support for its central claims.

While the computational framework appears technically sound and is demonstrated on several datasets, the evidence remains largely descriptive. The analyses focus on identifying differential fragmentation patterns, but there is limited demonstration that these patterns correspond to functional or mechanistic consequences. As a result, the broader biological interpretation, that fragmentation encodes regulatory information comparable to alternative splicing, feels premature.

Relatedly, the manuscript makes several strong claims regarding biological relevance and potential clinical applications, including biomarker discovery. These claims would require more direct validation. At present, the study relies entirely on computational analysis of existing datasets, and the absence of experimental validation (or orthogonal validation on independent datasets) makes it difficult to assess the robustness and generality of the conclusions. The identification of candidate fragments is promising, but their functional significance remains to be demonstrated.

The clarity of the manuscript could also be improved. Some aspects of the framework, particularly the definition and interpretation of fragmentation patterns and the statistical methodology, are not always presented in a sufficiently transparent way. In addition, the narrative occasionally blends current results with forward-looking statements, which can make it difficult to distinguish demonstrated findings from speculative implications.

Overall, the work presents an appealing idea and a potentially useful tool, but additional validation and clearer presentation would be needed to support the broader claims and make the study suitable for publication.

Response: We thank the reviewer for the overall positive comments and constructive suggestions. We agree that the previous manuscript was primarily computational and that direct functional evidence was not provided to support the biological relevance of *qMAP*-prioritized fragments. To address this concern, we have added a new sperm-aging application with experimental validation, where *qMAP* Model 1 identified 28S rRNA as the most significantly differentially fragmented parental RNA between young and old mouse sperm heads, and *qMAP_mh* further identified two prominent sequences for functional validation: one 17-nt rsRNA and one 44-nt rsRNA. The 44-nt rsRNA contains the 17-nt sequence at its 3' end, allowing comparison of nested fragments from the same parental rRNA locus. These two sequences are transfected into mESCs followed by transcriptomic analyses. As a result, genes upregulated in the 44-nt rsRNA group relative to the

17-nt group were enriched for DNA damage response, cell division, telomere maintenance, and apoptosis-related biological processes. These results provide proof-of-principle evidence that *qMAP*-prioritized fragments can have distinct cellular effects, as shown in **Fig. 6** and **Fig. EV6** in the revised manuscript. We have also added more methodological details regarding the statistical interpretation of differential fragmentation and the mapping strategy and discussed potential limitations in the Discussion section. In addition, we have tuned down our statements and modified the wording to distinguish experimentally supported conclusions from future implications. For the clinical analyses for recurrent implantation failure (RIF), we have added a statement as "*Prospective studies with larger cohorts, standardized sample processing, and predefined decision thresholds will be required before diagnostic application.*"

16th Jun 2026

Manuscript Number: MSB-2026-13661R
Title: qMAP decodes RNA fragmentation dynamics in development and disease

Dear Dr. Zhou,

Thank you for the submission of your revised manuscript to Molecular Systems Biology. We have now received the enclosed reports from the referees that were asked to re-assess it. As you will see the reviewers are now globally supportive and I am pleased to inform you that we will be able to accept your manuscript pending the following final amendments:

1) In the main manuscript file, please include keywords to max. 5.
2) Please format the Data availability section according to the example below.
"The datasets and computer code produced in this study are available in the following databases:
- Chip-Seq data: Gene Expression Omnibus GSE46748 (https://www.ncbi.nlm.nih.gov/geo/query/acc.cgi?acc=GSE46748)
- Modeling computer scripts: GitHub (https://github.com/SysBioChalmers/GECKO/releases/tag/v1.0)
- [data type]: [full name of the resource] [accession number/identifier] ([doi or URL or identifiers.org/DATABASE:ACCESSION])"
3) Please rename "Conflict of Interest" to "Disclosure and competing interests statement". In addition, any employment in a biotech company should be stated in this statement. We updated our journal's competing interests policy in January 2022 and request authors to consider both actual and perceived competing interests. Please review the policy https://link.springer.com/partners/embo-press/editorial-policies#Competing%20interest%20disclosures and update your competing interests if necessary.
4) In the Methods, please take care of the following:
- Please be sure to include a sentence in the Methods as to whether or not the cell lines were recently authenticated and whether they have been tested for mycoplasma contamination. Please also be sure to update the Author Checklist with this information and where it can be found in the manuscript.
- Please ensure that a statement on whether or not blinding was done is included in the Methods even if no blinding was done. Please also be sure to update the Author Checklist with this information and where it can be found in the manuscript.
5) Please place individual sections of the manuscript in the following order: Title page - Abstract & Keywords - Introduction - Results - Discussion - Methods - Data Availability - Acknowledgements - Disclosure and Competing Interests Statement - References - Figure Legends - (Main Tables with legends if applicable) - Expanded View Figure Legends.
6) For the figures and figure legends, please take care of the following:
- Please make sure to update the callouts of all figures in the main manuscript text. Currently callouts are missing for the individual panels for EV figures.
- EV figures should be uploaded as individual, high-resolution Figure files with the legends placed below the main figure legends. Please check "Author Guidelines" for more information: https://link.springer.com/journal/44320/submission-guidelines#cms-Expanded-View-data
- Please note that the exact p values are not provided in the legends of 3A; 5C; 6B,C; EV-6.
- Please indicate the statistical test used for data analysis in the legends of figure 2D; 3D; 6C,E; EV-2A; EV-3A; EV-5; EV-7.
- Please note that the box plots need to be defined in terms of minima, maxima, centre, bounds of box and whiskers, and percentile in the legends of figure 3C; 5B,E.
- Please note that information related to n is missing in the legends of 2C,E; 3C; 5B,E; EV-1A; EV-2A; EV-3A; EV-6.
7) EV tables should be uploaded as individual files and the EV table legends should be removed from the manuscript. Alternatively EV tables should be renamed to Appendix Tables compiled into a single Appendix PDF (no individual figure files needed for an Appendix). In the latter case, the title page of the Appendix should contain "Appendix for + manuscript title" (author and affiliation list not necessary) and a table of contents with the page numbers for the listed items. The nomenclature should be Appendix Figure Sx throughout the manuscript and Appendix PDF.
8) Please edit the synopsis image to fit to our required dimensions of 550 pixels wide x 300 pixels high.
9) As part of the EMBO Publications transparent editorial process initiative (see our policy here: https://www.embopress.org/transparent-process#Review_Process), Molecular Systems Biology will publish online a Peer Review File (PRF) to accompany accepted manuscripts. This file will be published in conjunction with your paper and will include the anonymous referee reports, your point-by-point response and all pertinent correspondence relating to the manuscript. Let us know whether you agree with the publication of the PRF and as here, if you want to remove or not any figures from it prior to publication. Please note that the Authors checklist will be published at the end of the PRF.
10) After your paper is published, we may promote it on social media. If you have any handles or hashtags for Bluesky you would like included, please let us know.
11) Please provide a point-by-point letter INCLUDING my comments and your detailed responses (as Word file).

I look forward to reading a new revised version of your manuscript as soon as possible.

Yours sincerely,

Poonam Bheda, PhD
Scientific Editor
Molecular Systems Biology

Reviewer #1:

The manuscript now addresses my prior critiques well, especially by clarifying how ambiguous and multi-mapped reads are handled, acknowledging that qMAP does not correct for RNA modification-related bias, and clarifying the recurrent implantation failure terminology.

Reviewer #2:

The authors have correctly addressed my earlier comments and also strengthened the manuscript by the inclusion of new original datasets. I have no further comments to make and recommend publication.

Reviewer #3:

The current revision significantly improves upon the original manuscript and adequately addresses my concerns.

Response to the Editor's comments

1) In the main manuscript file, please include keywords to max. 5.
Response: We thank the editor for this instruction. We have included the keywords in this revised manuscript.

2) Please format the Data availability section according to the example below.
"The datasets and computer code produced in this study are available in the following databases:
- Chip-Seq data: Gene Expression Omnibus GSE46748 (https://www.ncbi.nlm.nih.gov/geo/query/acc.cgi?acc=GSE46748)
- Modeling computer scripts: GitHub (https://github.com/SysBioChalmers/GECKO/releases/tag/v1.0)
- [data type]: [full name of the resource] [accession number/identifier] ([doi or URL or identifiers.org/DATABASE:ACCESSION])"

Response: We thank the editor for the example. We have re-formatted the Data Availability section accordingly.

3) Please rename "Conflict of Interest" to "Disclosure and competing interests statement". In addition, any employment in a biotech company should be stated in this statement. We updated our journal's competing interests policy in January 2022 and request authors to consider both actual and perceived competing interests. Please review the policy https://link.springer.com/partners/embo-press/editorial-policies#Competing%20interest%20disclosures and update your competing interests if necessary.
Response: We have renamed "Conflict of Interest" as "Disclosure and competing interests statement".

4) In the Methods, please take care of the following:
- Please be sure to include a sentence in the Methods as to whether or not the cell lines were recently authenticated and whether they have been tested for mycoplasma contamination. Please also be sure to update the Author Checklist with this information and where it can be found in the manuscript.
- Please ensure that a statement on whether or not blinding was done is included in the Methods even if no blinding was done. Please also be sure to update the Author Checklist with this information and where it can be found in the manuscript.

Response: In this revision, we have included a sentence in the Methods indicating "The mESCs were routinely tested for mycoplasma contamination, which have been confirmed to be negative." We have also included a statement regarding blinding in the Methods "No blinding was performed in this study." The Author Checklist was updated accordingly.

5) Please place individual sections of the manuscript in the following order: Title page - Abstract & Keywords - Introduction - Results - Discussion - Methods - Data Availability - Acknowledgements - Disclosure and Competing Interests Statement - References - Figure Legends - (Main Tables with legends if applicable) - Expanded View Figure Legends.
Response: We have re-ordered the individual sections according to the instructions in this revision.

6) For the figures and figure legends, please take care of the following:
- Please make sure to update the callouts of all figures in the main manuscript text. Currently callouts are missing for the individual panels for EV figures.
Response: We have updated the callouts of the EV figures.
- EV figures should be uploaded as individual, high-resolution Figure files with the legends placed below the main figure legends. Please check "Author Guidelines" for more information: https://link.springer.com/journal/44320/submission-guidelines#cms-Expanded-View-data
Response: The EV figures have been provided as individual files.
- Please note that the exact p values are not provided in the legends of 3A; 5C; 6B,C; EV-6.
Response: We have listed the exact *P*-values in the legends of Fig. 3A, 5C, 6B, and EV6. We don't have statistical *P*-value to report for Fig. 6C. The *P*-value information of the dots in Fig. 6C has been included in the Source Data.
- Please indicate the statistical test used for data analysis in the legends of figure 2D; 3D; 6C,E; EV-2A; EV-3A; EV-5; EV-7.
Response: The statistical approaches, *e.g.*, *qMAP* model 1/model 2, *qMAP_mh*, *David* tool (modified one-tailed *Fisher*'s exact test), were indicated in the legends.
- Please note that the box plots need to be defined in terms of minima, maxima, centre, bounds of box and whiskers, and percentile in the legends of figure 3C; 5B,E.
Response: We have included the definition of the box in the legend of Fig. 3 and 5 in this revision.
- Please note that information related to n is missing in the legends of 2C,E; 3C; 5B,E; EV-1A; EV-2A; EV-3A; EV-6.
Response: We have included the *n* information in Fig. 2, 3, 5, EV1, EV2, EV3, and EV6 in this revision.

7) EV tables should be uploaded as individual files and the EV table legends should be removed from the manuscript. Alternatively EV tables should be renamed to Appendix Tables compiled into a single Appendix PDF (no individual figure files needed for an Appendix). In the latter case, the title page of the Appendix should contain "Appendix for + manuscript title" (author and affiliation list not necessary) and a table of contents with the page numbers for the listed items. The nomenclature should be Appendix Figure Sx throughout the manuscript and Appendix PDF.
Response: The EV tables have been provided as individual files.

8) Please edit the synopsis image to fit to our required dimensions of 550 pixels wide x 300 pixels high.
Response: We thank the editor for the instruction. We have edited the synopsis image accordingly.

9) As part of the EMBO Publications transparent editorial process initiative (see our policy here: https://www.embopress.org/transparent-process#Review_Process), Molecular Systems Biology will publish online a Peer Review File (PRF) to accompany accepted manuscripts. This file will be published in conjunction with your paper and will include the anonymous referee reports, your point-by-point response and all pertinent correspondence relating to the manuscript. Let us know whether you agree with the publication of the PRF and as here, if you want to remove or not any figures from it prior to publication. Please note that the Authors checklist will be published at the end of the PRF.
Response: We thank the editor for the information. We agree with the publication of the PRF.

10) After your paper is published, we may promote it on social media. If you have any handles or hashtags for Bluesky you would like included, please let us know.
Response: We thank the editor for letting us know about this.

11) Please provide a point-by-point letter INCLUDING my comments and your detailed responses (as Word file).
Response: We have included a point-by-point response in this submission.

7th Jul 2026
Manuscript number: MSB-2026-13661RR
Title: qMAP decodes RNA fragmentation dynamics in development and disease

Dear Dr. Zhou,

Thank you again for sending us your revised manuscript. We are now satisfied with the modifications made and I am pleased to inform you that your paper has been accepted for publication.

Your manuscript will be processed for publication by EMBO Press. It will be copy edited and you will receive page proofs prior to publication. Please note that you will be contacted by Springer Nature Author Services to complete licensing and payment information.

You may qualify for financial assistance for your publication charges - either via a Springer Nature fully open access agreement or an EMBO initiative. Check your eligibility: https://link.springer.com/journal/44320/how-to-publish-with-us

Should you be planning a Press Release on your article, please notify the production team via this form in order to coordinate publication and release dates: https://forms.cloud.microsoft/e/eH4A8JVP6g

If you have any questions, please do not hesitate to contact the Editorial Office. Thank you for your contribution to Molecular Systems Biology.

Yours sincerely,

Poonam Bheda, PhD
Scientific Editor
Molecular Systems Biology

>>> Please note that it is Molecular Systems Biology policy for the transcript of the editorial process (containing referee reports and your response letter) to be published as an online supplement to each paper. If you do NOT want this, you will need to inform the Editorial Office via email immediately. More information is available here: https://link.springer.com/partners/embo-press/editorial-policies#Peer%20review

EMBO Press Author Checklist

Corresponding Author Name: Qi Chen, Tong Zhou
Journal Submitted to: Molecular Systems Biology
Manuscript Number: MSB-2026-13661

USEFUL LINKS FOR COMPLETING THIS FORM
The EMBO Journal - Author Guidelines
EMBO Reports - Author Guidelines
Molecular Systems Biology - Author Guidelines
EMBO Molecular Medicine - Author Guidelines

Reporting Checklist for Life Science Articles (updated January
This checklist is adapted from Materials Design Analysis Reporting (MDAR) Checklist for Authors. MDAR establishes a minimum set of requirements in transparent reporting in the life sciences (see Statement of Task: 10.31222/osf.io/9sm4x). Please follow the journal's guidelines in preparing your
Please note that a copy of this checklist will be published alongside your article.

Abridged guidelines for figures
1. Data
The data shown in figures should satisfy the following conditions:
- the data were obtained and processed according to the field's best practice and are presented to reflect the results of the experiments in an accurate and unbiased manner.
- ideally, figure panels should include only measurements that are directly comparable to each other and obtained with the same assay.
- plots include clearly labeled error bars for independent experiments and sample sizes. Unless justified, error bars should not be shown for technical
- if n<5, the individual data points from each experiment should be plotted. Any statistical test employed should be justified.
- Source Data should be included to report the data underlying figures according to the guidelines set out in the authorship guidelines on Data

2. Captions
Each figure caption should contain the following information, for each panel where they are relevant:
- a specification of the experimental system investigated (eg cell line, species name).
- the assay(s) and method(s) used to carry out the reported observations and measurements.
- an explicit mention of the biological and chemical entity(ies) that are being measured.
- an explicit mention of the biological and chemical entity(ies) that are altered/varied/perturbed in a controlled manner.
- the exact sample size (n) for each experimental group/condition, given as a number, not a range;
- a description of the sample collection allowing the reader to understand whether the samples represent technical or biological replicates (including how many animals, litters, cultures, etc.).
- a statement of how many times the experiment shown was independently replicated in the laboratory.
- definitions of statistical methods and measures:
  - common tests, such as t-test (please specify whether paired vs. unpaired), simple χ2 tests, Wilcoxon and Mann-Whitney tests, can be unambiguously identified by name only, but more complex techniques should be described in the methods section;
  - are tests one-sided or two-sided?
  - are there adjustments for multiple comparisons?
  - exact statistical test results, e.g., P values = x but not P values < x;
  - definition of 'center values' as median or average;
  - definition of error bars as s.d. or s.e.m.

Please complete ALL of the questions below.
Select "Not Applicable" only when the requested information is not relevant for your study.

Materials

Newly Created Materials	Information included in the manuscript?	In which section is the information available? (Reagents and Tools Table, Materials and Methods, Figures, Data Availability Section)
New materials and reagents need to be available; do any restrictions apply?	Yes	Reagents and Tools Table

Antibodies	Information included in the manuscript?	In which section is the information available? (Reagents and Tools Table, Materials and Methods, Figures, Data Availability Section)
For **antibodies** provide the following information:<br>- Commercial antibodies: RRID (if possible) or supplier name, catalogue number and or/clone number<br>- Non-commercial: RRID or citation	Not Applicable	

DNA and RNA sequences	Information included in the manuscript?	In which section is the information available? (Reagents and Tools Table, Materials and Methods, Figures, Data Availability Section)
**Short novel DNA or RNA including primers, probes**: provide the sequences.	Yes	Reagents and Tools Table

Cell materials	Information included in the manuscript?	In which section is the information available? (Reagents and Tools Table, Materials and Methods, Figures, Data Availability Section)
**Cell lines:** Provide species information, strain. Provide accession number in repository **OR** supplier name, catalog number, clone number, and/**OR** RRID.	Yes	Methods
**Primary cultures:** Provide species, strain, sex of origin, genetic modification status.	Yes	Methods
Report if the cell lines were recently **authenticated** (e.g., by STR profiling) and tested for mycoplasma contamination.	Yes	Methods

Experimental animals	Information included in the manuscript?	In which section is the information available? (Reagents and Tools Table, Materials and Methods, Figures, Data Availability Section)
**Laboratory animals or Model organisms:** Provide species, strain, sex, age, genetic modification status. Provide accession number in repository **OR** supplier name, catalog number, clone number, **OR** RRID.	Not Applicable	
**Animal observed in or captured from the field:** Provide species, sex, and age where possible.	Not Applicable	
Please detail **housing and husbandry conditions**.	Not Applicable	

Plants and microbes	Information included in the manuscript?	In which section is the information available? (Reagents and Tools Table, Materials and Methods, Figures, Data Availability Section)
**Plants:** provide species and strain, ecotype and cultivar where relevant, unique accession number if available, and source (including location for collected wild specimens).	Not Applicable	
**Microbes:** provide species and strain, unique accession number if available, and source.	Not Applicable	

Human research participants	Information included in the manuscript?	In which section is the information available? (Reagents and Tools Table, Materials and Methods, Figures, Data Availability Section)
If collected and within the bounds of privacy constraints report on age, sex and gender or ethnicity for all study participants.	Not Applicable	

Core facilities	Information included in the manuscript?	In which section is the information available? (Reagents and Tools Table, Materials and Methods, Figures, Data Availability Section)
If your work benefited from core facilities, was their service mentioned in the acknowledgments section?	Not Applicable	

Design

Study protocol	Information included in the manuscript?	In which section is the information available? (Reagents and Tools Table, Materials and Methods, Figures, Data Availability Section)
If study protocol has been **pre-registered, provide DOI in the manuscript**. For clinical trials, provide the trial registration number **OR** cite DOI.	Not Applicable	
Report the **clinical trial registration number** (at ClinicalTrials.gov or equivalent), where applicable.	Not Applicable	

Laboratory protocol	Information included in the manuscript?	In which section is the information available? (Reagents and Tools Table, Materials and Methods, Figures, Data Availability Section)
Provide DOI OR other citation details if **external detailed step-by-step protocols** are available.	Yes	Methods

Experimental study design and statistics	Information included in the manuscript?	In which section is the information available? (Reagents and Tools Table, Materials and Methods, Figures, Data Availability Section)
Include a statement about **sample size** estimate even if no statistical methods were used.	Yes	Methods
Were any steps taken to minimize the effects of subjective bias when allocating animals/samples to treatment (e.g. **randomization procedure**)? If yes, have they been described?	Not Applicable	
Include a statement about **blinding** even if no blinding was done.	Yes	Methods
Describe **inclusion/exclusion criteria** if samples or animals were excluded from the analysis. Were the criteria pre-established?<br><br>If sample or data points were omitted from analysis, report if this was due to attrition or intentional exclusion and provide justification.	Not Applicable	
For every figure, are **statistical tests** justified as appropriate? Do the data meet the assumptions of the tests (e.g., normal distribution)? Describe any methods used to assess it. Is there an estimate of variation within each group of data? Is the variance similar between the groups that are being statistically compared?	Yes	Methods

Sample definition and in-laboratory replication	Information included in the manuscript?	In which section is the information available? (Reagents and Tools Table, Materials and Methods, Figures, Data Availability Section)
In the figure legends: state number of times the experiment was **replicated** in laboratory.	Not Applicable	
In the figure legends: define whether data describe **technical or biological replicates**.	Yes	Figures

Ethics

Ethics	Information included in the manuscript?	In which section is the information available? (Reagents and Tools Table, Materials and Methods, Figures, Data Availability Section)
Studies involving **human participants**: State details of **authority granting ethics approval** (IRB or equivalent committee(s), provide reference number for approval.	Not Applicable	
Studies involving **human participants**: Include a statement confirming that **informed consent** was obtained from all subjects and that the experiments conformed to the principles set out in the WMA Declaration of Helsinki and the Department of Health and Human Services Belmont Report.	Not Applicable	
Studies involving **human participants:** For publication of **patient photos**, include a statement confirming that consent to publish was obtained.	Not Applicable	
Studies involving experimental **animals**: State details of **authority granting ethics approval** (IRB or equivalent committee(s), provide reference number for approval. Include a statement of compliance with ethical regulations.	Not Applicable	
Studies involving **specimen and field samples:** State if relevant **permits** obtained, provide details of authority approving study; if none were required, explain why.	Not Applicable	

Dual Use Research of Concern (DURC)	Information included in the manuscript?	In which section is the information available? (Reagents and Tools Table, Materials and Methods, Figures, Data Availability Section)
Could your study fall under dual use research restrictions? Please check biosecurity documents and list of **select agents and toxins** (CDC): https://www.selectagents.gov/sat/list.htm	Not Applicable	
If you used a select agent, is the security level of the lab appropriate and reported in the manuscript?	Not Applicable	
If a study is subject to dual use research of concern regulations, is the name of the **authority granting approval and reference number** for the regulatory approval provided in the manuscript?	Not Applicable	

Reporting

The MDAR framework recommends adoption of discipline-specific guidelines, established and endorsed through community initiatives. Journals have their own policy about requiring specific guidelines and recommendations to complement MDAR.

Adherence to community standards	Information included in the manuscript?	In which section is the information available? (Reagents and Tools Table, Materials and Methods, Figures, Data Availability Section)
State if relevant guidelines or checklists (e.g., **ICMJE, MIBBI, ARRIVE, PRISMA**) have been followed or provided.	Not Applicable	
For **tumor marker prognostic studies,** we recommend that you follow the **REMARK** reporting guidelines (see link list at top right). See author guidelines, under 'Reporting Guidelines'. Please confirm you have followed these guidelines.	Not Applicable	
For **phase II and III randomized controlled trials**, please refer to the **CONSORT** flow diagram (see link list at top right) and submit the CONSORT checklist (see link list at top right) with your submission. See author guidelines, under 'Reporting Guidelines'. Please confirm you have submitted this list.	Not Applicable	

Data Availability

Data availability	Information included in the manuscript?	In which section is the information available? (Reagents and Tools Table, Materials and Methods, Figures, Data Availability Section)
Have **primary datasets** been deposited according to the journal's guidelines (see 'Data Deposition' section) and the respective accession numbers provided in the Data Availability Section?	Yes	Data Availabity
Were **human clinical and genomic datasets** deposited in a public access-controlled repository in accordance to ethical obligations to the patients and to the applicable consent agreement?	Not Applicable	
Are **computational models** that are central and integral to a study available without restrictions in a machine-readable form? Were the relevant accession numbers or links provided?	Yes	Data Availability
If publicly available data were reused, provide the respective **data citations in the reference list**.	Yes	References
